# Cognitive behavioural therapy for adults with dissociative seizures (CODES): a pragmatic, multicentre, randomised controlled trial

**DOI:** 10.1016/S2215-0366(20)30128-0

**Published:** 2020-06

**Authors:** Laura H Goldstein, Emily J Robinson, John D C Mellers, Jon Stone, Alan Carson, Markus Reuber, Nick Medford, Paul McCrone, Joanna Murray, Mark P Richardson, Izabela Pilecka, Carole Eastwood, Michele Moore, Iris Mosweu, Iain Perdue, Sabine Landau, Trudie Chalder, CODES study group, A-M Abe, N Adab, N Agrawal, H Allroggen, D Alvares, T Andrews, H Angus-Leppan, J Aram, R Armstrong, A Atalaia, M Bagary, M Baldellou Lopez, M Bennett, T Black, D Blackburn, M Bodani, M Broadhurst, A Brockington, E Bruno, M Buckley, C Burness, H Callaghan, R Chalmers, S Chong, M Chowdhury, F Chowdury, K Cikurel, G Cocco, H Cock, S Cooper, S Cope, A Copping, E Day, R Delamont, G Dennis, C Derry, R Devlin, J.M. Dickson, B Diehl, C Donnelly, S Duncan, M Edwards, S Ellawella, C Ellis, J Elvish, R Elwes, S Eriemo, S Eriksson, K Evans, R Faruqui, S Feehan, G Finnerty, L Flores, N Firth, R Fung, P Gardiner, C Graham, Z Green-Thompson, R Grunewald, R Hadden, K Hamandi, R Harding, S Harikrishnan, S Harrison, H Healy, C Hewamadduma, S Higgins, S Howell, H Hunt, A Hussain, M Innocente, G Jensch, M Johnson, H Jordan, J Karlsson, A Kelso, S Kemp, J Knibb, N Kock, M Koutroumanidis, S Kovac, G Kumar, A Laker, G Leschziner, R Liu, D Lozsadi, L Ludwig, B MacDonald, L MacGregor, M Maguire, M Manford, D Martino, D McCorry, A McGorlick, K McKeown, F McKevitt, A Meadow, S Memon, A Miorelli, C Mitchell, T.N. Mitchell, V Moffitt, N Moran, A Morgan-Boon, J Moriarty, M Mula, N Mullatti, L Nashef, D O'Hara, L Oakley, S O'Sullivan, L Page, D Patel, P Petrochilos, D Phoenix, W Pickerell, T Pieters, N Poole, G Price, D Protheroe, P Pullicino, J Purnell, J Quirk, S Rajakulendran, J Read, B Ridha, C Rockliffe-Fidler, C Rowbottom, F Rugg-Gunn, A Sachar, R Saha, G Saldanha, S Samarasekera, V Sanchez Sanchez, A Santhouse, K Scholes, A Shetty, P Shotbolt, R Simkiss, J Singh, J Sivagnanasundaram, S Slaght, P Smith, D Sokhi, B Stanton, L Suvorova, T Tahir, R Taylor, L Teare, L Tedesco, J Teo, J Thorpe, L Toplis, M Tsakopoulou, I Tylova, T Vick, J Vinnicombe, M Walker, C Walsh, G Watson, T Webb, T Wehner, K Welch, K Weyrich, M Whittaker, M Wickremaratchi, L Wicks, M Yogarajah

**Affiliations:** a Department of Psychology, King's College London, London, UK; b Department of Biostatistics and Health Informatics, King's College London, London, UK; c King's Health Economics, King's College London, London, UK; d Department of Health Services and Population Research, King's College London, London, UK; e Department of Basic and Clinical Neuroscience, King's College London, London, UK; f Department of Psychological Medicine, King's College London, London, UK; g Institute of Psychiatry, Psychology and Neuroscience, and School of Population Health and Environmental Sciences, King's College London, London, UK; h Department of Neuropsychiatry, South London and Maudsley NHS Foundation Trust, London, UK; i Department of Clinical Neuroscience, Centre for Clinical Brain Sciences, University of Edinburgh, Edinburgh, UK; j Academic Neurology Unit, Royal Hallamshire Hospital, University of Sheffield, Sheffield, UK; k Centre for Social Justice and Global Responsibility, School of Law and Social Sciences, London South Bank University, London, UK

## Abstract

**Background:**

Dissociative seizures are paroxysmal events resembling epilepsy or syncope with characteristic features that allow them to be distinguished from other medical conditions. We aimed to compare the effectiveness of cognitive behavioural therapy (CBT) plus standardised medical care with standardised medical care alone for the reduction of dissociative seizure frequency.

**Methods:**

In this pragmatic, parallel-arm, multicentre randomised controlled trial, we initially recruited participants at 27 neurology or epilepsy services in England, Scotland, and Wales. Adults (≥18 years) who had dissociative seizures in the previous 8 weeks and no epileptic seizures in the previous 12 months were subsequently randomly assigned (1:1) from 17 liaison or neuropsychiatry services following psychiatric assessment, to receive standardised medical care or CBT plus standardised medical care, using a web-based system. Randomisation was stratified by neuropsychiatry or liaison psychiatry recruitment site. The trial manager, chief investigator, all treating clinicians, and patients were aware of treatment allocation, but outcome data collectors and trial statisticians were unaware of treatment allocation. Patients were followed up 6 months and 12 months after randomisation. The primary outcome was monthly dissociative seizure frequency (ie, frequency in the previous 4 weeks) assessed at 12 months. Secondary outcomes assessed at 12 months were: seizure severity (intensity) and bothersomeness; longest period of seizure freedom in the previous 6 months; complete seizure freedom in the previous 3 months; a greater than 50% reduction in seizure frequency relative to baseline; changes in dissociative seizures (rated by others); health-related quality of life; psychosocial functioning; psychiatric symptoms, psychological distress, and somatic symptom burden; and clinical impression of improvement and satisfaction. p values and statistical significance for outcomes were reported without correction for multiple comparisons as per our protocol. Primary and secondary outcomes were assessed in the intention-to-treat population with multiple imputation for missing observations. This trial is registered with the International Standard Randomised Controlled Trial registry, ISRCTN05681227, and ClinicalTrials.gov, NCT02325544.

**Findings:**

Between Jan 16, 2015, and May 31, 2017, we randomly assigned 368 patients to receive CBT plus standardised medical care (n=186) or standardised medical care alone (n=182); of whom 313 had primary outcome data at 12 months (156 [84%] of 186 patients in the CBT plus standardised medical care group and 157 [86%] of 182 patients in the standardised medical care group). At 12 months, no significant difference in monthly dissociative seizure frequency was identified between the groups (median 4 seizures [IQR 0–20] in the CBT plus standardised medical care group *vs* 7 seizures [1–35] in the standardised medical care group; estimated incidence rate ratio [IRR] 0·78 [95% CI 0·56–1·09]; p=0·144). Dissociative seizures were rated as less bothersome in the CBT plus standardised medical care group than the standardised medical care group (estimated mean difference −0·53 [95% CI −0·97 to −0·08]; p=0·020). The CBT plus standardised medical care group had a longer period of dissociative seizure freedom in the previous 6 months (estimated IRR 1·64 [95% CI 1·22 to 2·20]; p=0·001), reported better health-related quality of life on the EuroQoL-5 Dimensions-5 Level Health Today visual analogue scale (estimated mean difference 6·16 [95% CI 1·48 to 10·84]; p=0·010), less impairment in psychosocial functioning on the Work and Social Adjustment Scale (estimated mean difference −4·12 [95% CI −6·35 to −1·89]; p<0·001), less overall psychological distress than the standardised medical care group on the Clinical Outcomes in Routine Evaluation-10 scale (estimated mean difference −1·65 [95% CI −2·96 to −0·35]; p=0·013), and fewer somatic symptoms on the modified Patient Health Questionnaire-15 scale (estimated mean difference −1·67 [95% CI −2·90 to −0·44]; p=0·008). Clinical improvement at 12 months was greater in the CBT plus standardised medical care group than the standardised medical care alone group as reported by patients (estimated mean difference 0·66 [95% CI 0·26 to 1·04]; p=0·001) and by clinicians (estimated mean difference 0·47 [95% CI 0·21 to 0·73]; p<0·001), and the CBT plus standardised medical care group had greater satisfaction with treatment than did the standardised medical care group (estimated mean difference 0·90 [95% CI 0·48 to 1·31]; p<0·001). No significant differences in patient-reported seizure severity (estimated mean difference −0·11 [95% CI −0·50 to 0·29]; p=0·593) or seizure freedom in the last 3 months of the study (estimated odds ratio [OR] 1·77 [95% CI 0·93 to 3·37]; p=0·083) were identified between the groups. Furthermore, no significant differences were identified in the proportion of patients who had a more than 50% reduction in dissociative seizure frequency compared with baseline (OR 1·27 [95% CI 0·80 to 2·02]; p=0·313). Additionally, the 12-item Short Form survey–version 2 scores (estimated mean difference for the Physical Component Summary score 1·78 [95% CI −0·37 to 3·92]; p=0·105; estimated mean difference for the Mental Component Summary score 2·22 [95% CI −0·30 to 4·75]; p=0·084), the Generalised Anxiety Disorder-7 scale score (estimated mean difference −1·09 [95% CI −2·27 to 0·09]; p=0·069), and the Patient Health Questionnaire-9 scale depression score (estimated mean difference −1·10 [95% CI −2·41 to 0·21]; p=0·099) did not differ significantly between groups. Changes in dissociative seizures (rated by others) could not be assessed due to insufficient data. During the 12-month period, the number of adverse events was similar between the groups: 57 (31%) of 186 participants in the CBT plus standardised medical care group reported 97 adverse events and 53 (29%) of 182 participants in the standardised medical care group reported 79 adverse events.

**Interpretation:**

CBT plus standardised medical care had no statistically significant advantage compared with standardised medical care alone for the reduction of monthly seizures. However, improvements were observed in a number of clinically relevant secondary outcomes following CBT plus standardised medical care when compared with standardised medical care alone. Thus, adults with dissociative seizures might benefit from the addition of dissociative seizure-specific CBT to specialist care from neurologists and psychiatrists. Future work is needed to identify patients who would benefit most from a dissociative seizure-specific CBT approach.

**Funding:**

National Institute for Health Research, Health Technology Assessment programme.

## Introduction

Dissociative seizures are paroxysmal episodes of altered awareness, resembling epileptic seizures or syncope, which are not explained by these or other medical disorders and usually have distinctive clinical features.^1^ Between 12% and 20% of adults presenting in epilepsy clinics have dissociative seizures.^2^ Prevalence estimates range between 2 and 50 cases per 100 000 individuals;^3^ a UK-based study observed an annual incidence of around 4·9 cases per 100 000 individuals.^4^ We have adopted the term dissociative seizures in preference to others (eg, psychogenic non-epileptic seizures, non-epileptic attack disorder) because it is consistent with widely used classifications (ie, ICD-10),^5^ highlights that the diagnosis is not solely a process of exclusion, and conveys a mechanism that can be discussed with patients.

Around 75% of adults with dissociative seizures are women.^6^ Modal onset of dissociative seizures is between the late teenage years and early twenties, but dissociative seizures can develop at any point across the lifespan.^6^ Most adults with dissociative seizures present with a range of comorbid psychiatric and psychological difficulties. Outcome is generally poor,^6^ quality of life is worse than for people with epilepsy,^7^ and health service use among individuals with dissociative seizures is high.^8^

The most consistent predictive factor of poor outcome for people with dissociative seizures seems to be the duration of symptoms, whereby a longer duration of the disorder is associated with negative outcomes.^9^, ^10^, ^11^ Additional factors that negatively predict outcomes include the receipt of state financial benefits,^12^ having previous psychiatric diagnoses,^12^ and other evidence of psychopathology^13^ and interpersonal difficulties.^14^ Employment,^4^, ^15^ better educational attainment and intelligence quotient,^16^, ^17^ and less evidence of somatisation^18^ have been found to predict better outcome.

In the UK, no standardised care pathway exists for people with dissociative seizures. Although psychological treatment has been considered the treatment of choice, the availability of such treatment is variable,^19^ despite guidance^20^ that when dissociative seizures are suspected, referral should be made to psychiatric or psychological services for evaluation and intervention.

Psychological interventions might be associated with a reduction in dissociative seizures,^21^ although seizures might also remit spontaneously^9^ or after information provision.^22^ However, no adequately powered randomised controlled trials have been done to assess the use of psychotherapeutic treatments.^23^, ^24^ A small, four-arm pilot randomised controlled trial in 34 patients^24^ evaluated outcome in terms of dissociative seizure frequency and a range of secondary outcomes following 12 sessions of cognitive behavioural therapy (CBT)-informed psychotherapy with or without flexible-dose sertraline, sertraline alone, or treatment as usual. Within-group analyses showed that both CBT-informed psychothotherapy groups had a significant reduction in dissociative seizure frequency during the treatment period (CBT-informed psychothotherapy (n=9) 51·4% reduction, p=0·01; CBT-informed psychothotherapy plus sertraline (n=9) 59·3% reduction, p=0·008) over 16 weeks, and improvement in a range of secondary outcomes, including global functioning. No significant differences in dissociative seizure frequency were identified in the sertraline-alone group (n=9; p=0·08) or the treatment as usual group (n=7; p=0·19). Our 2010 proof-of-concept randomised controlled trial^23^ including 66 patients indicated that dissociative seizure-specific CBT plus neuropsychiatric care could lead to a reduction in dissociative seizure frequency at the end of treatment compared with neuropsychiatric treatment alone; however, between-group differences were not significant 6 months after the end of treatment. These findings informed the design of this randomised controlled trial. We aimed to assess the effectiveness of dissociative seizure-specific CBT in reducing the frequency of dissociative seizures and improving functioning compared with standardised medical care after 12 months of follow-up.

**Research in context**

**Evidence before this study**

For patients with dissociative seizures, the general consensus is that careful explanation of the diagnosis followed by psychotherapy is the treatment of choice, although to date, no standardised treatment pathways have been established globally. A 2014 Cochrane review highlighted the paucity of adequate randomised controlled trials in this field. Existing studies were small or did not include dissociative seizure occurrence as an outcome. The best evidence was considered to be from a 2010 pilot randomised controlled trial study, which evaluated the benefit of dissociative seizure-specific cognitive behavioural therapy (CBT) for adults with dissociative seizures plus standard medical (neuropsychiatric) care, compared with standard medical care alone. That proof-of-concept study, which followed on from an uncontrolled study and a single case study, indicated that a dissociative seizure-specific CBT package plus standard medical care led to a greater reduction in dissociative seizures at the end of treatment than did standard care alone, although at 6-month follow-up, the between-group difference was non-significant.

We searched PubMed from database inception to Oct 16, 2019, without language restrictions, using the search terms “([therapy and dissociative seizures)]” OR “([CBT and dissociative seizures])” OR “([therapy and psychogenic nonepileptic seizures])” OR “([CBT and psychogenic nonepileptic seizures])” OR “([therapy and PNES])” OR “([CBT and PNES])” OR “([therapy and nonepileptic attack disorder])” OR “([CBT and nonepileptic attack disorder])” OR “([therapy and NEAD])” OR “([CBT and NEAD])”. Our search yielded one additional underpowered pilot randomised controlled trial that assessed CBT-informed psychotherapy alone, CBT-informed psychotherapy with pharmacotherapy, pharmacotherapy, and treatment as usual, but no adequately powered multi-site effectiveness studies.

**Added value of this study**

Our study compared standardised medical care alone with dissociative seizure-specific CBT plus standardised medical care. Standardised medical care aimed to provide an optimised clinical pathway involving neurologists and psychiatrists with standardised communication guidelines and materials. Our randomised controlled trial of adults with dissociative seizures is the largest to date to assess a psychological intervention in this patient group. Although no statistically significant difference in dissociative seizure frequency at 12 months was identified between the CBT plus standardised medical care and standardised medical care alone groups, significant improvements were identified for a number of clinically relevant secondary outcomes (highest number of consecutive dissociative seizure-free days in the past 6 months, functional status, self-rated and clinician-rated change in global impression scores, and satisfaction with treatment) among the CBT plus standardised medical care group when compared with the standardised medical care alone group. This pragmatic study shows that although dissociative seizure-specific CBT did not result in a statistically significant reduction in the primary outcome of monthly dissociative seizures when compared with standardised medical care, the combined treatment approach might improve a number of relevant clinical outcome measures.

**Implications of all the available evidence**

Although no significant difference in monthly dissociative seizure frequency was identified between the treatment groups, this study expands on previous evidence suggesting that CBT specific to dissociative seizures might be beneficial for adults with dissociative seizures and demonstrates that the application of such an intervention does not have to be limited to highly specialised centres. The additional implementation of dissociative seizure-specific, manualised, and individualised CBT can be delivered by clinical psychologists or cognitive behavioural psychotherapists. Therapy resulted in improvements in a range of dissociative seizure-related and psychosocial secondary outcomes in the CBT plus standardised medical care group compared with standardised medical care alone.

## Methods

### Study design and participants

In this pragmatic, parallel-group, multicentre randomised controlled trial, we initially recruited participants from 27 neurology or epilepsy services in England, Scotland, and Wales. Eligible participants were adults (≥18 years) who had dissociative seizures in the previous 8 weeks (diagnosis preferably confirmed by video-electroencephalogram [EEG] or, if not available, clinical consensus provided by two consultants involved in the patient's care or by expert review of the clinical records and relevant investigations by one of two neurologists in the research team), without a documented history of intellectual disabilities, who were able to complete seizure diaries and questionnaires, and were willing to have a psychiatric assessment at 3 months after diagnosis.^25^, ^26^ Due to the uncertainty of being able to provide interpreters for CBT sessions, only patients with sufficient English proficiency to enable completion of therapy and questionnaires without an interpreter were included. Patients with comorbid epilepsy could only participate if they had been free of epileptic seizures for 12 months, and patients who met DSM-IV criteria for current drug or alcohol dependence were excluded. Around 3 months after enrolment and consent for the initial screening phase of the study was obtained, participants were assessed in one of 17 neuropsychiatry or liaison psychiatry services. The 3-month delay was implemented to avoid recruitment of patients whose dissociative seizures might remit quickly after delivery of a diagnosis and information provision alone^22^ and to allow time for appointments to be arranged. Participants who met further eligibility criteria^25^, ^26^ and provided consent were recruited. The main approved modification implemented after publication of the study protocol and analysis plan was to extend the number of participants screened in the neurology and epilepsy settings and numbers randomly assigned to ensure adequate participants at follow-up. The study was approved by the London-Camberwell St Giles Research Ethics Committee. The study protocol and statistical analysis plan have been published previously.^25^, ^26^ All participants provided separate written informed consent for the 3-month observation period and the randomised controlled trial.

### Randomisation and masking

Participants were randomly assigned (1:1) to standardised medical care or CBT plus standardised medical care, by either a designated researcher or the trial manager using a web-based system maintained by the King's Clinical Trials Unit (London, UK). Randomisation was stratified by neuropsychiatry or liaison psychiatry recruitment site (randomly varying block sizes within strata). In view of the nature of the interventions, the trial manager, chief investigator, all treating clinicians, and patients were aware of treatment allocation, but outcome data collectors and trial statisticians were unaware of treatment allocation.

### Procedures

Patients were informed of the dissociative seizures diagnosis by an epilepsy specialist or neurologist using suggested diagnosis communication guidelines.^25^ Patients were provided with a neurology trial-specific information booklet about dissociative seizures. After patient consent was obtained for the 3-month observation period, we collected demographic information, study participants were instructed in seizure diary completion, and seizure data were collected every 2 weeks by a researcher. The neurologist who had explained the dissociative seizures diagnosis referred the participant to a psychiatrist in a specified National Health Service (NHS) clinic for an appointment around 3 months later. At this appointment, the psychiatrist reviewed the diagnosis and discussed it with the patient using suggested guidelines, did a clinical psychiatric assessment, provided the patient with a further psychiatry trial-specific information booklet, and assessed the patient's eligibility for the randomised controlled trial. Eligible patients who were willing to be contacted about the trial were seen by a researcher who confirmed eligibility, obtained consent to participate in the randomised controlled trial, and did baseline assessments before randomisation. The interventions are described in detail in the appendix (pp 4–7) and have been published previously.^25^

To assess the fidelity of CBT delivery, after obtaining patient consent we audio-recorded therapy sessions. Two experienced CBT therapists who had not provided therapy in the trial blindly rated one session (chosen at random from one of two preselected sessions) from each of 36 therapists for whom we had usable session recordings (39 therapists overall). The raters evaluated therapists' adherence to the treatment manual, whether they could be considered to be delivering CBT, the quality of the therapeutic alliance, and the extent to which dissociative seizure-specific CBT skills were used in sessions, on the basis of identified competencies.^27^ Items were scored on a 0–7 scale. Scores were standardised; a score of 100 indicated best possible performance.

All outcomes were assessed at 12 months after randomisation (appendix pp 8–53).^26^ Outcome measures were also obtained at baseline and at 6 months after randomisation to maintain participant involvement and inform modelling of the 12-month trial outcomes (appendix pp 8–53). Patients were asked to complete seizure diaries for each week of the study, and were contacted every 2 weeks for these data or, where diary data were not available, data were obtained from a single self-report question about seizures over the previous 4 weeks. For patients randomly assigned to receive CBT, compliance with treatment was defined as attendance at nine or more CBT sessions.^26^

Further clinical and demographic data were collected at recruitment into the screening phase or immediately before randomisation: a locally devised measure of the patients' level of agreement with the diagnosis of dissociative seizures (collected at screening); pre-randomisation administration of the Mini-International Neuropsychiatric Interview (MINI) version 6.0;^28^ the Self-report Standardised Assessment of Personality-abbreviated Scale (SAPAS-SR) for maladaptive personality traits (0=no traits, 8=all traits),^29^ and the Index of Multiple Deprivation.^30^, ^31^, ^32^

### Outcomes

The primary outcome was monthly seizure frequency (ie, dissociative seizure frequency in the previous 4 weeks) assessed at 12 months. Construction of the primary outcome measure is explained in the appendix (pp 75–76).

Secondary outcomes assessed at 12 months were seizure severity (intensity) and bothersomeness measured using the Seizure Severity Scale; longest period of seizure freedom in the previous 6 months; the proportion of patients with complete seizure freedom during the final 3 months of the study; the proportion of patients who had more than 50% reduction in seizure frequency relative to baseline; informants' (ie, carers or others nominated by patients) ratings of changes in the patients' dissociative seizures; health-related quality of life (12-item Short Form 12 Survey–version 2 [SF-12v2] scores comprising Physical Component Summary and Mental Component Summary scores; and the EQ-5D-5L visual analogue scale); psychosocial functioning (Work and Social Adjustment Scale [WSAS]); psychiatric symptoms and psychological distress (Generalised Anxiety Disorder-7 scale, Patient Health Questionnaire [PHQ]-9 depression measure, Clinical Outcomes in Routine Evaluation-10 [CORE-10]); somatic symptom burden (modified PHQ-15,^33^ which measured self-reported presence or absence of 30 symptoms [15 common symptoms seen in primary care, ten neurological symptoms, and five psychological symptoms]); and clinical impression of improvement (Clinical Global Impression score [rated by the patient and by the psychiatrist or neurologist]) and satisfaction with treatment. All secondary outcomes are listed in the appendix (pp 8–53).^25^

Additionally, CBT therapists measured improvement on the Clinical Global Impression score for patients receiving CBT at the end of session 12.

The study protocol^25^ (appendix pp 8–53) also included a health economics evaluation (using the Client Service Receipt Inventory, EQ-5D-5L, Short-Form six-dimension utility index derived from the SF-12v2, and hospital episode statistics) and a process evaluation, which will be reported elsewhere. Further secondary data analyses to investigate mediator and moderator hypotheses (avoidance of people, places and situations; 12-item Beliefs About Emotions Scale; belief in diagnosis of dissociative seizures; and belief in being given the correct treatment)^25^ are planned and will be reported elsewhere.

We defined serious adverse events as any adverse event that resulted in death; was life-threatening; required hospital admission or prolongation of existing hospital stay; resulted in a new persistent or new significant disability or incapacity; was any other important medical condition that might jeopardise the participant and might require medical or surgical intervention to prevent one of the outcomes listed; or any new episode of deliberate self-harm. We also recorded reports of suicidal ideation as serious adverse events. All adverse events and serious adverse events were reviewed by three independent raters. Adverse events were recorded for the entire 12-month follow-up period. Participants were specifically asked about illnesses and hospital admissions at the 6-month and 12-month follow-up timepoints, but they were also reported by clinicians and research workers whenever they became aware of them.

### Statistical analysis

Sample size and power calculations have been described previously (appendix pp 8–53).^25^, ^26^ Briefly, we calculated that 149 participants per group were needed to provide 92·6% power at the 5% significance level. Our target randomised controlled trial sample size was changed from 298 to 356 to accommodate lower than anticipated follow-up rates (appendix p 54).

The analyses followed the agreed statistical analysis plan,^26^ published before database lock. All analyses were done using Stata (version 15.0). Descriptive statistics summarising pre-randomisation variables and outcome measures at 6 and 12 months were reported by treatment group and overall.

Primary and secondary outcomes were assessed in the intention-to-treat population with multiple imputation used to facilitate the inclusion of all randomised participants in formal analyses. The effectiveness of CBT plus standardised medical care intervention was determined by assessing the between-group differences in primary or secondary outcome measures at 12 months.^26^ To ensure that the trial statisticians remained unaware of treatment allocation for as long as possible, an independent statistician determined that treatment compliance within the intervention group (attendance at nine or more CBT sessions) was predictive of observed 12-month primary outcome values (Fisher's exact test p<0·001). Therefore, we used multiple imputation with 100 imputations to produce inferences that are valid under such a missing at random data-generating process.^34^ Multiple imputation requires the specification of an analysis and an imputation model; details of these models are provided in the appendix (pp 76–79). Briefly, for overdispersed count variables (seizure frequency and seizure freedom), both imputation and analysis models assumed a negative binomial distribution. For continuous and discrete outcome variables, such as seizure severity or bothersomeness, modelling was based on a normal distribution. Logistic regression models were used for binary outcomes. We report original inferences (p values or CIs were not corrected for multiple secondary outcome comparisons).

We did two sensitivity analyses. First, agreement between the two different methods of recording the primary outcome (diary or questionnaire item) was assessed by calculating an intraclass correlation coefficient on the log scale, and the sensitivity of the finding to the recording method was assessed by repeating the multiple imputation analysis after treating patients who did not provide the diary measure (considered the gold standard) as missing. Second, all 17 outcomes were re-analysed on a complete case basis (ie, without imputation or adjustment for baseline predictors of missingness). To assess the efficacy of CBT in the presence of non-compliance, the complier average causal effect (CACE) was estimated for the primary outcome; log-transformed seizure frequency was considered the dependent variable and treatment receipt as the explanatory variable and its effect estimated by a two-stage least squares estimator. Multiple imputation was used as described previously.

The trial was overseen by a Trial Management Group, Trial Steering Committee, and Data Monitoring and Ethics Committee. This trial is registered with International Standard Randomised Controlled Trial registry, ISRCTN05681227, and ClinicalTrials.gov, NCT02325544.

### Role of the funding source

The funder had no role in study design, data collection, data analysis, data interpretation, or writing of the report. The corresponding author had access to all study data and had final responsibility for the decision to submit for publication.

## Results

**Figure 1:**
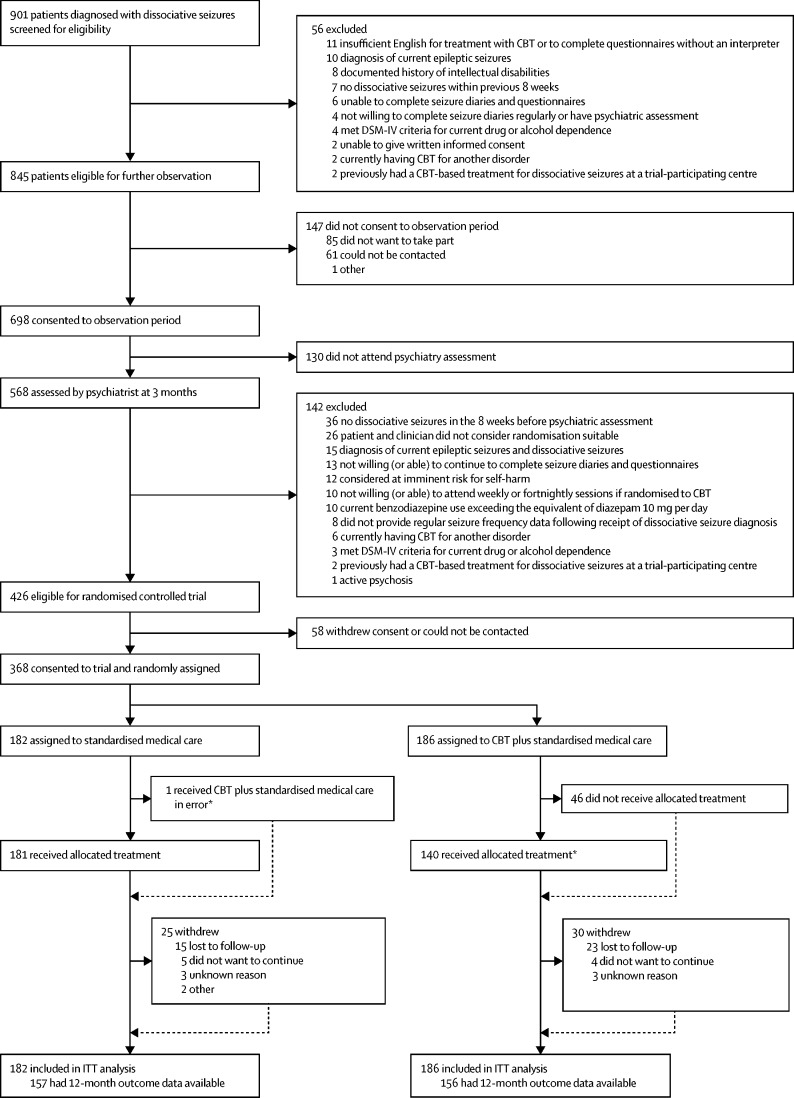
Trial profile The number of standardised medical care sessions offered and attended per group, and the number of CBT sessions attended in the CBT plus standardised medical care group are presented in the appendix (p 68). Multiple imputation was used to facilitate an ITT analysis; all randomised participants contributed to the statistical analysis. CBT=cognitive behavioural therapy. ITT=intention-to-treat analysis. *Treatment receipt of CBT was defined as attendance at nine or more sessions (as per definition of compliance).

Between Jan 16, 2015, and May 31, 2017, from the 698 people who were intially recruited to the study,^35^ we randomly assigned 368 patients to standardised medical care alone (n=182) or CBT plus standardised medical care (n=186; figure 1). Recruitment by site is shown in the appendix (pp 55–57). Demographic data for the 368 participants who participated in the trial and the 58 patients who were eligible for the randomised controlled trial but could not be recruited are shown in the appendix (pp 58–59). No significant differences in key characteristics measured during the 3-month observation period were identified between the two groups.

Baseline characteristics were balanced between the treatment groups (table 1; appendix pp 60–61). Most participants were women, white, unemployed, living in areas of high deprivation, and had at least one comorbid psychiatric diagnosis (appendix pp 62–64) with a median of two current MINI diagnoses (range 0–8). 211 (58%) of 363 patients had SAPAS-SR scores suggestive of maladaptive personality traits. Age at onset was variable (range 1–76 years) with a peak at 19 years (median 29 years [IQR 19–42]). The range of the duration of the dissociative seizure disorder was also variable (range 0–65 years) with a median duration of 3 years (IQR 1–8; table 1).

**Table 1:** Baseline demographic and clinical characteristics of the intention-to-treat population (n=368)

		**Standardised medical care (n=182)**	**CBT plus standardised medical care (n=186)**	**Total (n=368)**
Age, years				
	Mean (SD)	37·7 (14·5)	37·3 (14·2)	37·5 (14·3)
	Median (IQR; range)	35 (25–49; 18–77)	35 (25– 47; 18–78)	35 (25–48; 18–78)
Gender*				
	Female	126 (69%)	140 (75%)	266 (72%)
	Male	56 (31%)	46 (25%)	102 (28%)
Ethnicity*				
	White	163 (90%)	167 (90%)	330 (90%)
	Asian	4 (2%)	2 (1%)	6 (2%)
	Black	1 (1%)	5 (3%)	6 (2%)
	Mixed	9 (5%)	8 (4%)	17 (5%)
	Other	5 (3%)	4 (2%)	9 (2%)
Relationship status				
	Single, separated, or widowed	85 (47%)	88 (47%)	173 (47%)
	Married or living with partner	97 (53%)	98 (53%)	195 (53%)
Living with others		158 (87%)	158 (85%)	316 (86%)
Has dependants		57 (31%)	64 (34%)	121 (33%)
Has a carer		69 (38%)	80 (43%)	149 (40%)
Qualifications†				
	None	21/181 (12%)	22/186 (12%)	43/367 (12%)
	Secondary	41/181 (23%)	48/186 (26%)	89/367 (24%)
	Vocational	66/181 (36%)	54/186 (29%)	120/367 (33%)
	Further (A-level or equivalent)	28/181 (15%)	28/186 (15%)	56/367 (15%)
	Higher (BSc or equivalent and higher)	25/181 (14%)	34/186 (18%)	59/367 (16%)
Currently employed or in education*		58/180 (32%)	65/185 (35%)	123/365 (34%)
Receiving disability benefits if working age (<65 years)*				
	Yes (unemployed)	86/115 (75%)	79/118 (67%)	165/233 (71%)
	Yes (employed)	13/58 (22%)	5/52 (10%)	18/110 (16%)
Dissociative seizures diagnosed by EEG and video*		94 (52%)	101 (54%)	195 (53%)
Age at first dissociative seizure, years*‡				
	Mean (SD)	30·9 (14·6)	31·0 (13·5)	30·9 (14·1)
	Median (IQR; range)	29 (19–42; 5–76)	29 (19–41·5; 1–67)	29 (19–42; 1–76)
Duration of dissociative seizure symptoms, years*‡				
	Mean (SD)	6·5 (9·7)	5·9 (7·8)	6·2 (8·8)
	Median (IQR; range)	3 (1–8; 0–65)	3 (1–7·5; 0–44)	3 (1–8; 0–65)
Predominant dissociative seizure type*				
	Hypokinetic	60/181 (33%)	70/185 (38%)	130/366 (36%)
	Hyperkinetic	121/181 (67%)	115/185 (62%)	236/366 (64%)
Mean belief in diagnosis score (SD; range)*§		8·0 (2·2; 0–10)	8·0 (2·2; 0–10)	8·0 (2·2; 0–10)
Previous diagnosis of epilepsy (patient reported)		52 (29%)	49 (26%)	101 (27%)
Previous diagnosis of epilepsy* (doctor reported)		53 (29%)	36 (19%)	89 (24%)
Currently prescribed any anti-epileptic drugs (patient reported)		36 (20%)	40 (22%)	76 (21%)
Ever sought medical help for a mental health problem		116 (64%)	125 (67%)	241 (65%)
Comorbid medical conditions		131/181 (72%)	130/184 (71%)	261/365 (72%)
Screening tool for maladaptive personality traits (SAPAS-SR)				
	Mean (SD; range)	4·0 (2·0; 0–8)	3·9 (1·9; 0–8)	3·9 (2·0; 0–8)
	Patients with ≥4 traits	108/181 (60%)	103/182 (57%)	211/363 (58%)
Any current DSM-IV diagnosis¶		125 (69%)	130 (70%)	255 (69%)
Any previous DSM-IV diagnosis¶		112 (62%)	135 (73%)	247 (67%)

Data are n (%) or n/N (%), unless otherwise specified. CBT=cognitive behavioural therapy. EEG=electroencephalogram. SAPAS-SR=Standardised Assessment of Personality Abbreviated Scale Self Report.

* Recorded after consent was obtained for the 3-month screening period.

† Qualifications based on UK educational system.

‡ Data available for 181 patients in the standardised medical care group and 184 patients in the CBT plus standardised medical care group.

§ Data available for 181 patients in the standardised medical care group and 185 patients in the CBT plus standardised medical care group.

¶ Based on the Mini-International Neuropsychiatric Interview.

The demographic characteristics of the clinicians treating study participants, the number of standardised medical care sessions attended in both treatment groups, and the number of CBT sessions attended are shown in the appendix (pp 65–69). The number of standardised medical care appointments offered and attended were similar across treatment groups. One patient allocated to standardised medical care alone received our dissociative seizure-specific CBT in error. 104 (56%) of 186 participants in the CBT plus standardised medical care group attended all 12 CBT sessions (plus the booster session) and 140 (75%) of 186 patients were compliant according to our criterion^26^ (attending nine sessions or more). The median time between randomisation and first CBT session was 38·5 days (IQR 26–59). Ten (5%) of 186 patients formally withdrew from CBT. Therapists' ratings of patients' acceptance of the therapy model and adherence to treatment between sessions are shown in the appendix (pp 68–69). Therapy fidelity ratings for the 36 rated sessions across the rated categories show good therapeutic alliance, adherence to the therapy manual, and delivery of CBT (appendix p 70).

Primary outcome data were available for 313 (85%) of 368 patients: 157 (86%) of 182 patients in the standardised medical care group alone versus 156 (84%) of 186 in the CBT plus standardised medical care group. No study withdrawals or losses to follow-up were deemed to be associated with reported adverse events or death.

**Figure 2:**
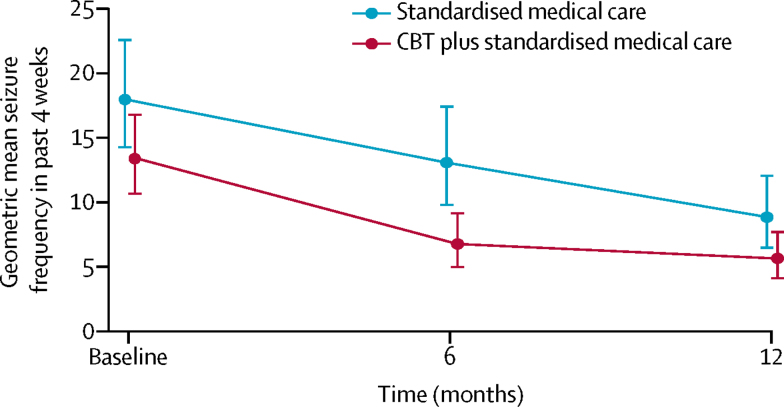
Changes in geometric mean seizure frequency over time Raw data plot of geometric mean seizure frequency in the past 4 weeks. Baseline seizure frequency was recorded before randomisation. The primary outcome was measured at 12 months; 6-month measures were not formally assessed in this study, but are included here to illustrate observed data. Error bars show 95% CIs. CBT=cognitive behavioural therapy.

Primary and secondary outcome measures at all timepoints are summarised in table 2. For the primary outcome, no significant differences in monthly dissociative seizure frequency were identified between the groups at 12 months (median 4 seizures [IQR 0–20] in the CBT plus standardised medical care group *vs* 7 seizures [1–35] in the standardised medical care group; estimated incidence rate ratio [IRR] 0·78 [95% CI 0·56–1·09]; p=0·144; table 3, figure 2). Although dissociative seizure frequency seemed to decrease with time in both groups, dissociative seizure frequency seemed to decrease at an earlier timepoint in the CBT plus standardised medical care group than in the standardised medical care group (table 2, figure 2). At 12 months, participants in the CBT plus standardised medical care group rated their seizures as less bothersome than the standardised medical care group (estimated mean difference −0·53 [95% CI −0·97 to −0·08]; p=0·020), but no significant differences were identified in patient-reported seizure severity between the groups (p=0·593; table 3). No significant differences were identified in the proportion of patients with seizure freedom in the last 3 months of the study (p=0·083) or the proportion of patients who had a more than 50% reduction in dissociative seizure frequency between the two groups (p=0·313); however, at 12 months participants in the CBT plus standardised medical care group had nearly two-thirds longer periods of seizure freedom in the previous 6 months than did patients in the standardised medical care group (IRR 1·64 [95% CI 1·22–2·20; p=0·001; table 3). Only 27 carers or others nominated by patients provided a rating of changes in the patients' seizures; thus, this outcome could not be analysed.

**Table 2:** Descriptive summaries of primary and secondary outcome measures

	**Baseline**			**6 months**			**12 months**		
	Standardised medical care (n=182)	CBT plus standardised medical care (n=186)	Overall (n=368)	Standardised medical care (n=182)	CBT plus standardised medical care (n=186)	Overall (n=368)	Standardised medical care (n=182)	CBT plus standardised medical care (n=186)	Overall (n=368)
**Monthly seizure frequency**									
Patients with available data, n (%)	182 (100%)	186 (100%)	368 (100%)	162 (89%)	161 (87%)	323 (88%)	157 (86%)	156 (84%)	313 (85%)
Median seizure frequency (IQR; range)	19 (5–49;0–649)	12·5 (4–41; 0–535)	15 (4–47;0–649)	18 (3–48;0–640)	6 (0–24;0–849)	9 (1–38;0–849)	7 (1–35;0–994)	4 (0–20;0–571)	5 (0–27;0–994)
Seizure severity*									
Patients with available data, n (%)	179 (98%)	182 (98%)	361 (98%)	135 (74%)	125 (67%)	260 (71%)	130 (71%)	129 (69%)	259 (70%)
Mean score (SD; range)	4·8 (1·6;1–7)	4·7 (1·6;1–7)	4·7 (1·6;1–7)	4·4 (1·6;1–7)	3·9 (1·9;1–7)	4·1 (1·8;1–7)	4·1 (1·8;1–7)	3·8 (1·8;1–7)	4·0 (1·8;1–7)
**Seizure bothersomeness**†									
Patients with available data, n (%)	180 (99%)	182 (98%)	362 (98%)	143 (79%)	134 (72%)	277 (75%)	132 (73%)	131 (70%)	263 (71%)
Mean score (SD; range)	5·4 (1·7;1–7)	5·2 (1·7;1 −7)	5·3 (1·7;1–7)	4·7 (2·0;1–7)	3·9 (2·1;1–7)	4·3 (2·1;1–7)	4·6 (2·1;1–7)	3·9 (2·0;1–7)	4·2 (2·1;1–7)
**Longest period of seizure freedom in past 6 months**									
Patients with available data, n (%)	181 (99%)	186 (100%)	367 (100%)	NA	NA	NA	143 (79%)	140 (75%)	283 (77%)
Median number of seizure-free days* (IQR; range)	7 (2–21;0–84)	7 (2–21;0 −119)	7 (2–21;0–119)	NA	NA	NA	12 (3–42;0–343)	21 (5–97·5;0–357)	14 (3–70;0–357)
**Seizure freedom in past 3 months**									
Patients with available data, n (%)	NA	NA	NA	NA	NA	NA	145 (80%)	148 (80%)	293 (80%)
Yes, n (%)	NA	NA	NA	NA	NA	NA	18 (12%)	29 (20%)	47 (16%)
No, n (%)	NA	NA	NA	NA	NA	NA	127 (88%)	119 (80%)	246 (84%)
**>50% reduction in monthly seizure frequency relative to baseline**									
Patients with available data, n (%)	NA	NA	NA	157 (86%)	153 (82%)	310 (84%)	152 (84%)	149 (80%)	301 (82%)
Yes, n (%)	NA	NA	NA	43 (27%)	65 (42%)	108 (35%)	60 (39%)	68 (46%)	128 (43%)
No, n (%)	NA	NA	NA	114 (73%)	88 (58%)	202 (65%)	92 (61%)	81 (54%)	173 (57%)
**SF-12v2**‡									
Patients with available data, n (%)	181 (99%)	185 (99%)	366 (99%)	142 (78%)	134 (72%)	276 (75%)	145 (80%)	148 (80%)	293 (80%)
Mean Physical Component Summary score (SD; range)	38·8 (11·9;13·9–65·6)	40·5 (12·4;13·4–65·9)	39·7 (12·2;13·4–66·0)	38·8 (11·4;13·1–59·5)	41·5 (13·4;15·9–66·7)	40·1 (12·4;13·1–66·7)	38·0 (12·6;10·4–63·7)	41·5 (13·4;12·2–67·3)	39·8 (13·1;10·4–67·3)
Mean Mental Component Summary score (SD; range)	37·9 (11·4;16·9–68·1)	37·7 (12·2;13·4–67·6)	37·8 (11·8;13·4–68·1)	37·5 (12·1;10·5–63·0)	40·3 (11·7;17·4–67·5)	38·8 (12·0;10·5–67·5)	39·5 (11·8;11·3–62·9)	41·5 (12·8;13·9–65·7)	40·5 (12·4;11·3–65·7)
**EQ-5D-5L visual analogue scale**‡									
Patients with available data, n (%)	181 (99%)	182 (98%)	363 (99%)	143 (79%)	135 (73%)	278 (76%)	145 (80%)	148 (80%)	293 (80%)
Mean score (SD; range)	54·9 (21·9;10–100)	56·2 (24·1;1–100)	55·5 (23·0;1–100)	50·9 (23·1;0–100)	58·8 (24·4;0–100)	54·7 (24·0;0–100)	53·4 (22·6;5–100)	61·1 (24·0;5–100)	57·3 (23·6;5–100)
**WSAS**									
Patients with available data, n (%)	181 (99%)	185 (99%)	366 (99%)	143 (79%)	135 (73%)	278 (76%)	145 (80%)	148 (80%)	293 (80%)
Mean score§ (SD; range)	22·9 (10·5;0–40)	22·5 (10·5;0–40)	22·7 (10·5;0–40)	22·7 (11·9;0–40)	17·8 (13·1;0–40)	20·3 (12·7;0–40)	21·1 (12·7;0–40)	16·4 (13·1;0–40)	18·7 (13·1;0–40)
**GAD-7 scale**									
Patients with available data, n (%)	182 (100%)	186 (100%)	368 (100%)	143 (79%)	135 (73%)	278 (76%)	145 (80%)	148 (80%)	293 (80%)
Mean score¶ (SD; range)	10·0 (6·2;0–21)	9·6 (6·2;0–21)	9·8 (6·2;0–21)	10·5 (6·3;0–21)	8·1 (6·5;0–21)	9·4 (6·5;0–21)	9·3 (6·1;0–21)	8·2 (6·0; 0–21)	8·8 (6·1;0–21)
**PHQ-9**									
Patients with available data, n (%)	181 (99%)	186 (100%)	367 (100%)	142 (78%)	135 (73%)	277 (75%)	145 (80%)	148 (80%)	293 (80%)
Mean score‖ (SD; range)	12·6 (6·5;0–26)	12·3 (6·7;0–27)	12·4 (6·6;0–27)	12·9 (7·0;0–27)	11·2 (7·4;0–27)	12·1 (7·2;0–27)	11·7 (6·7;0–26)	10·5 (7·5;0–26)	11·1 (7·1;0–26)
**CORE-10**									
Patients with available data, n (%)	182 (100%)	186 (100%)	368 (100%)	142 (78%)	135 (73%)	277 (75%)	145 (80%)	148 (80%)	293 (80%)
Mean score§ (SD; range)	18·2 (6·3;4–34)	18·2 (6·7;4–32)	18·2 (6·5;4–34)	18·6 (6·6;2–34)	17·2 (7·1;0–39)	17·9 (6·9;0–39)	18·1 (6·6;3–33)	16·6 (6·8;1–38)	17·3 (6·7;1–38)
**Modified PHQ-15**									
Patients with available data, n (%)	181 (99%)	183 (98%)	364 (99%)	140 (77%)	135 (73%)	275 (75%)	145 (80%)	147 (79%)	292 (79%)
Mean score** (SD; range)	16·7 (6·2;2–30)	16·7 (6·8;2–30)	16·7 (6·5;2–30)	16·8 (6·7;0–29)	14·9 (7·4;0–28)	15·9 (7·1;0–29)	15·9 (6·9;0–29)	14·1 (7·7;0–28)	15·0 (7·4;0–29)
**Clinical global impression of improvement**††									
Patients with available data, n (%)	NA	NA	NA	140 (77%)	135 (73%)	275 (75%)	145 (80%)	148 (80%)	293 (80%)
Mean self-reported change score (SD; range)	NA	NA	NA	3·4 (1·6;0–6)	4·2 (1·3;0–6)	3·8 (1·5;0–6)	3·6 (1·8;0–6)	4·3 (1·5;0–6)	4·0 (1·7;0–6)
Patients with available data, n (%)	NA	NA	NA	NA	NA	NA	162 (89%)	161 (87%)	323 (88%)
Mean clinician-rated change score (SD; range)	NA	NA	NA	NA	NA	NA	3·8 (1·3;0–6)	4·4 (1·2;0–6)	4·1 (1·3;0–6)
**Patient satisfaction with treatment**‡‡									
Patients with available data, n (%)	NA	NA	NA	140 (77%)	135 (73%)	275 (75%)	145 (80%)	148 (80%)	293 (80%)
Mean satisfaction score (SD; range)	NA	NA	NA	3·8 (2·0;0–6)	5·1 (1·3;0–6)	4·4 (1·8;0–6)	4·2 (2·0;0–6)	5·2 (1·4;0–6)	4·7 (1·8;0–6)

CBT=cognitive behavioural therapy. SF-12v2=12-item Short Form 12 Survey–version 2. EQ-5D-5L=EuroQoL-5 Dimensions-5 Level scale. WSAS=Work and Social Adjustment Scale. GAD-7=Generalised Anxiety Disorder seven-item. PHQ-9=Patient Health Questionnaire nine-item. CORE-10=Clinical Outcomes in Routine Evaluation-10. PHQ-15=Patient Health Questionnaire fifteen-item.

* Measured on a 7-point scale (1=very mild; 7=very severe).

† Measured on a 7-point scale (1=no bother at all; 7=very bothersome).

‡ Measured on a 100 point scale (0=worst health; 100=best health).

§ Possible range 0–40.

¶ Possible range 0–21.

‖ Possible range 0–27.

** Possible range 0–30.

†† Change scores measured on a 7-point scale (0=very much worse; 6=very much better).

‡‡ Measured on a 7-point scale (0=very dissatisfied; 6=very satisfied).

**Table 3:** Comparison of outcome measures between the CBT plus standardised medical care and standardised medical care alone groups at 12 months derived by multiple imputation (100 imputations)

	Estimated mean difference*(95% CI)	**Standardised group difference (95% CI)**	**p value**
**Primary outcome**			
Monthly seizure frequency in last 4 weeks	NA	0·78 (0·56 to 1·09)†	0·144
**Secondary outcomes**			
Seizure severity score	−0·11 (−0·50 to 0·29)	−0·07 (−0·31 to 0·18)	0·593
Seizure bothersomeness severity score	−0·53 (−0·97 to −0·08)	−0·30 (−0·56 to −0·05)	0·020‡
Longest period of seizure freedom in past 6 months (days)	NA	1·64 (1·22 to 2·20)†	0·001†
Seizure freedom in last 3 months of trial	NA	1·77 (0·93 to 3·37)§	0·083
>50% reduction in monthly seizure frequency relative to baseline	NA	1·27 (0·80 to 2·02)§	0·313
Physical Component Summary score (SF-12v2)	1·78 (−0·37 to 3·92)	0·15 (−0·03 to 0·32)	0·105
Mental Component Summary score (SF-12v2)	2·22 (−0·30 to 4·75)	0·15 (−0·03 to 0·33)	0·084
EQ-5D-5L visual analogue scale	6·16 (1·48 to 10·84)	0·27 (0·06 to 0·47)	0·010†
Impact on functioning (WSAS)	−4·12 (−6·35 to −1·89)	−0·39 (−0·61 to −0·18)	<0·001†
Anxiety (GAD-7)	−1·09 (−2·27 to 0·09)	−0·18 (−0·37 to 0·01)	0·069
Depression (PHQ-9)	−1·10 (−2·41 to 0·21)	−0·17 (−0·37 to 0·03)	0·099
Distress (CORE-10)	−1·65 (−2·96 to −0·35)	−0·25 (−0·45 to −0·05)	0·013‡
Other somatic symptoms (modified PHQ-15)	−1·67 (−2·90 to −0·44)	−0·26 (−0·45 to −0·07)	0·008‡
Self-reported change (CGI score)	0·66 (0·26 to 1·04)	0·39 (0·16 to 0·62)	0·001‡
Clinician-rated change (CGI score)	0·47 (0·21 to 0·73)	0·37 (0·17 to 0·57)	<0·001‡
Patient-reported satisfaction with treatment	0·90 (0·48 to 1·31)	0·50 (0·27 to 0·73)	<0·001‡

p values not adjusted for multiple testing. Standardised group differences between 0·35 and 0·65 were considered moderate. NA=not applicable. SF-12v2=12-item Short Form survey–version 2. EQ-5D-5L=EuroQoL-5 Dimensions-5 Level scale. WSAS=Work and Social Adjustment Scale. GAD-7=Generalised Anxiety Disorder seven-item. PHQ-9=Patient Health Questionnaire nine-item. CORE-10=Clinical Outcomes in Routine Evaluation-10. PHQ-15=Patient Health Questionnaire fifteen-item. CGI=Clinical Global Impression.

* Using original scales.

† Treatment effects for count outcomes are presented as incidence rate ratios.

‡ Statistically significant at 5% level (not accounting for multiple testing).

§ Treatment effects for binary outcomes are presented as odds ratios.

The SF-12v2 Physical Component Summary and Mental Component Summary measures of health-related quality of life did not significantly differ between groups at 12 months (p=0·105 and p=0·084, respectively; table 3). However, the CBT plus standardised medical care group reported a better health rating on the EQ-5D-5L visual analogue scale than did the standardised medical care group (estimated mean difference 6·16 [95% CI 1·48 to 10·84]; p=0·010; table 3).

Psychosocial functioning (measured by WSAS) was significantly better in the CBT plus standardised medical care group than the standardised medical care group at 12 months (estimated mean difference −4·12 [95% CI −6·35 to −1·89]; p<0·001; table 3). No significant differences in anxiety (p=0·069) and depression (p=0·099) scores were identified between the groups at 12 months (table 3); however, the more general measure of psychological distress (CORE-10) showed better psychological functioning in the CBT plus standardised medical care group than the standardised medical care group (estimated mean difference −1·65 [95% CI −2·96 to −0·35]; p=0·013; table 3). The CBT plus standardised medical care group reported fewer somatic symptoms, as measured by the modified PHQ-15, than the standardised medical care group (estimated mean difference −1·67 [95% CI −2·90 to −0·44]; p=0·008) (table 3).

The CBT plus standardised medical care group self-reported greater clinical improvement than did the standardised medical care group (estimated mean difference 0·66 [95% CI 0·26 to 1·04; p=0·001; table 3) with a similar pattern observed for the standardised medical care clinicians' ratings of patients' improvement (estimated mean difference 0·47 [95% CI 0·21 to 0·73]; p<0·001; table 3). Raw data plots to illustrate observed changes in all secondary outcomes over time and by treatment group are shown in the appendix (pp 83–90).

The CBT plus standardised medical care group also reported greater satisfaction with treatment than the standardised medical care group (estimated mean difference 0·90 [95% CI 0·48 to 1·31; p<0·001; table 3; appendix p 71).

**Figure 3:**
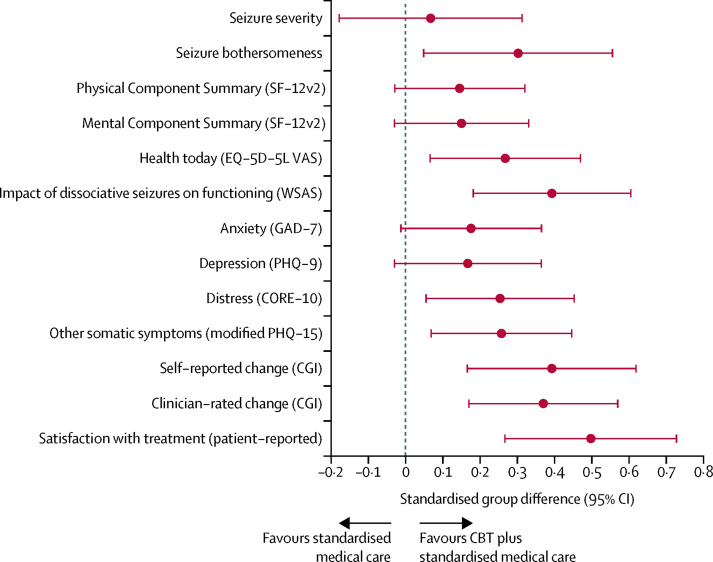
Standardised group differences for all secondary outcomes analysed on a continuous scale Forest plot of standardised group differences between CBT plus standardised medical care and standardised medical care groups for all 13 continuous secondary outcomes, whereby a standardised treatment effect higher than 0 favoured the CBT plus standardised medical care group. Error bars show 95% CIs**.** CBT=cognitive behavioural therapy. SF-12v2=12-item Short Form 12 Survey–version 2. EQ-5D-5L=EuroQoL-5 Dimensions-5 Levels scale. VAS=visual analogue scale. WSAS=Work and Social Adjustment Scale. GAD-7=Generalised Anxiety Disorder-7 scale. PHQ-9=Patient Health Questionnaire-9 scale. CORE-10=Clinical Outcomes in Routine Evaluation-10. PHQ-15=Patient Health Questionnaire-15 scale. CGI=Clinical Global Impression.

The size of the standardised treatment effects for all secondary outcomes analysed on a continuous scale are shown in figure 3. For table 3 outcomes that were interpreted as being in favour of CBT plus standardised medical care if the standardised group difference was less than 0, the estimated effect was reflected (ie, multiplied by −1) in figure 3 so that outcomes for which the standardised group difference was higher than 0 could be interpreted as being in favour of the intervention.

The p values in table 3 have not been adjusted for multiple testing.^26^ Considering conservative adjustment (eg, post-hoc Bonferroni correction of 0·05/17=0·003), five secondary outcomes suggest that CBT plus standardised medical care provided significant benefit to participants compared with standardised medical care: longest period of seizure freedom, psychosocial functioning, self-rated and clinician-rated global change, and treatment satisfaction (table 3).

CBT therapists rated 82 (54%) of 152 patients who received CBT as much better or very much better at the end of treatment (appendix p 71).

Sensitivity analyses showed high agreement between the questionnaire and diary records (intraclass correlation coefficient=0·95); exclusion of 21 participants without diary data at 12 months resulted in the same finding for the primary outcome (IRR 0·74 [95% CI 0·53–1·04]; p=0·086). Complete case analyses of the primary and secondary outcomes are shown in the appendix (p 72). Comparison with table 3 showed that use of multiple imputation to adjust for missing data had little effect on the results, with similar findings for the primary outcome and the secondary outcomes. CACE analysis modelled the effect of receiving the intervention (at least nine sessions of CBT) and estimated the efficacy of the intervention, rather than its effectiveness (estimated by ITT analysis). We found the efficacy of receiving CBT was estimated to be the same as the effectiveness of being offered CBT (IRR 0·78, 95% CI 0·53–1·16; p=0·217).

During the entire 12-month follow-up period, 110 participants had 176 adverse events: 57 (31**%**) of 186 participants in the CBT plus standardised medical care group reported 97 adverse events and 53 (29%) of 182 participants in the standardised medical care group reported 79 adverse events. Psychological events were the most common type of adverse event in the CBT plus standardised medical care group (n=24), which included deteriorations in mood and musculoskeletal events were the most common type of adverse event reported in the standardised medical care group (n=17), such as reports of injuries from dissociative seizures. All serious adverse events and adverse events are shown in the appendix (pp 73–74). No adverse events or serious adverse events were deemed to be associated with the CBT plus standardised medical care intervention by our independent raters. The incidence of serious adverse events was similar across both groups (24 [13%] of 182 participants in the standardised medical care alone group *vs* 25 [13%] of 186 patients in the CBT plus standardised medical care group; table 4). Considering other potential indices of harm, only one patient had a decline of at least 20 points (2 SD) on the SF-12v2 Physical Component Summary. At 12 months, on the Clinical Global Impression scale, 25 (17%) of 145 patients in the standardised medical care group and 13 (9%) of 148 patients in the CBT plus standardised medical care group self-reported being much worse or very much worse.

**Table 4:** Serious adverse events

	**Standardised medical care (n=182)**		**CBT plus standardised medical care (n=186)**	
	Events	Participants	Events	Participants
Self-harm or suicidal ideation	6	6 (3%)	12	9 (5%)
Related to dissociative seizures	6	6 (3%)	5	5 (3%)
Other	15	14 (8%)	14	14 (8%)
Total	27	24 (13%)	31	25 (13%)

Data are n or n (%). CBT=cognitive behavioural therapy.

## Discussion

To our knowledge, this is the largest randomised controlled trial that has been done to date, assessing a psychotherapeutic intervention for adults with dissociative seizures, in the context of a care pathway involving both neurology and psychiatry services. Our analysis showed that at 12 months after randomisation no significant differences in dissociative seizure frequency were observed between the CBT plus standardised medical care and standardised medical care groups. For nine of 16 secondary outcome measures, between-group differences were significant at the unadjusted 5% level (p≤0·05). These data suggest that compared with standardised medical care alone, CBT plus standardised medical care provided particular benefits with regard to the longest period of seizure freedom at 12 months, psychosocial functioning, self-rated and clinician-rated global change, and treatment satisfaction and all showed standardised group differences in the moderate range. Treatment compliance among patients in the intervention group was high (75%) and therapists showed good adherence to the therapy manual, and were found to be delivering CBT as intended.

By contrast with our earlier study,^23^ this trial suggests that dissociative seizure-specific CBT could contribute to improvement in clinically important aspects of psychosocial functioning and perceptions of health among patients with dissociative seizures. These improvements were observed despite the observation that at baseline more than half of the cohort self-reported maladaptive personality traits, more than two-thirds had at least one other comorbid mental health disorder, and participants did not enter the study until dissociative seizures had already been present for a median of 3 years. Before treatment, the cohort also had below average health-related quality-of-life scores on the SF-12v2.^36^ Patients also reported mean scores on the WSAS consistent with at least moderately severe functional impairment when summarising across the domains of work, home management, social and private leisure activities, and the ability to form and maintain interpersonal relationships.^37^ Furthermore, self-rated health on the EQ-5D-5L visual analogue scale was scored considerably below the national average.^38^ Increasing evidence suggests that seizure frequency alone is not the most important determinant of quality of life in patients with dissociative seizures. A 2017 study^39^ found that mood, anxiety, and illness perceptions were the factors most closely linked with quality of life in patients with dissociative seizures, emphasising the importance of the secondary outcome effects in this trial. These effects are particularly notable since therapists might have faced additional challenges because patients were not preselected by therapists for their suitability for a cognitive behavioural intervention, which might be the case in routine service delivery.

A wider issue raised in this study is that of best practice for treatment trials of dissociative seizures. This study followed a standard clinical trial approach of offering a specific intervention to a patient group and measuring outcomes. However, particular subgroups of patients might be more likely to respond to a CBT-based treatment, and others more likely to respond to other psychotherapeutic or pharmacological interventions. This idea is commonly applied in clinical practice—eg, a specific patient might be deemed unlikely to engage meaningfully with a psychotherapeutic intervention, even if that intervention is generally regarded as a treatment of choice for the condition in question. These are judgements that rely on clinical impression but to base such decisions on empirical evidence would require formal trials of treatment regimens tailored to subgroups of patients. Similar comments could be made regarding treatment studies of many other conditions, particularly those that involve a complex interplay of psychological and physical factors that might vary considerably between individuals, even if their symptoms are similar. However, it might be possible to identify factors that make individuals more likely to respond to particular interventions. This issue is outside the scope of this Article, but we hope to explore it further in subsequent statistical analysis of the impact of moderating and mediating variables on treatment outcomes in this trial, which will be reported separately.

Regarding study limitations, we did not include a waiting list or treatment-as-usual control group and, thus, are unable to determine whether the apparent reduction in dissociative seizure frequency observed in both groups at 12 months represents equal effectiveness of the treatment allocations or the natural progression of the disorder in our sample. We are not aware of data on dissociative seizure outcome with no intervention over 12 months with which to compare our findings, although an uncontrolled retrospective study done over a longer period of time, without a psychological intervention, in a different cultural context, suggests that more than 50% of patients with dissociative seizures might become seizure free in the absence of psychotherapy,^9^ but it is not clear how quickly such improvement occurs. The control condition in this study might have been an active therapeutic intervention and, with the materials provided for patients and guidance given to clinicians about diagnosis delivery and management, might be better conceptualised as standardised specialist care than standardised medical care (appendix p 82).

Regarding other study limitations, our randomised controlled trial included a cohort who were selected using several inclusion criteria. Only individuals who were initially recruited after diagnosis by neurologists or epilepsy specialists and attended their psychiatric assessment could enrol. We excluded people with active epilepsy (ie, epileptic seizures in the previous 12 months) but acknowledge that where we relied on self-report for this criterion, this could potentially have led to the inclusion of some people with active seizures^40^ despite careful consensus review and the possibility of further review at the time of the psychiatric assessment. We restricted our sample to people without documented intellectual disabilities and without concurrent comorbid epileptic seizures to make evaluation of the individualised CBT more interpretable. Due to the uncertainty of being able to provide interpreters, only patients with sufficient English proficiency to enable completion of therapy and questionnaires without an interpreter were included. Consistent with usual clinical practice in the UK, diagnoses of dissociative seizures in our sample were supported by video-EEG recordings of typical dissociative seizure events in about half of all participants.^19^ In line with previous international expert recommendations,^41^ and to ensure that the findings of the study were more readily generalisable to routine UK practice, we also sought to include patients for whom diagnosis had been reached without video-EEG confirmation. The risk of misdiagnosis, if clinical consensus had not already been obtained, was reduced in these cases by seeking expert review of the clinical records and relevant investigations and exclusion of patients with questionable diagnoses, although we did not classify levels of diagnostic certainty.^41^ Video-EEG confirmation was not compulsory in this pragmatic trial since this diagnostic method is not available or cost-effective for many in routine clinical use, and treatment might be appropriately started at less than optimum levels of diagnostic certainty.^41^

Although randomisation would be expected to have led to similar baseline levels of psychotropic medication use in the two trial groups, we did not record the prescription of antidepressant and other psychotropic medication in the two treatment groups and cannot be sure that the standardised medical care alone group did not receive more of this type of treatment as a substitute for psychotherapeutic input than the CBT plus standardised medical care group; this is unfortunate considering the interest in the use of antidepressants for dissociative seizures either alone or in combination with CBT-informed psychotherapy.^24^

Both groups were contacted frequently by researchers and were required to complete seizure diaries throughout the study, which might have acted as an intervention, not typically available in the NHS. Both patients and their treating doctors were aware of treatment allocation. Although doctors might have altered their practice depending on treatment allocation, patients received a similar number of standardised medical care sessions in the two groups. We did not, however, control for therapist time and attention. Although many patients will have come to the study with trauma histories, and the CBT intervention did address trauma, this was not specifically a trauma-focused therapy. We acknowledge that at the end of the study additional therapy might have been considered useful for some.

Although dissociative seizure frequency was our primary outcome, the usefulness of this as an outcome has been questioned.^42^ Recording this outcome consistently in a large sample poses difficulties (appendix pp 75–76). Functional status might be a more meaningful outcome, especially if it occurs in the context of better ability to tolerate persistent dissociative seizures, enabling better everyday activity. We also note that it is unfortunate that non-adherence with CBT was predictive of missing outcome data but, wherever possible, unless individuals formally withdrew from the trial, we encouraged follow-up irrespective of treatment experience, but we could not insist that people provide outcome data when they discontinued attending CBT; however, the proportion of patients who provided outcome data was balanced between treatment groups.

The strengths of this study include the pragmatic design, large sample size, high follow-up rates, outcome assessments (other than clinician ratings) done by a masked researcher, and the large number of centres and therapists involved, which removed potential bias occurring in smaller study designs. The good compliance with CBT suggests that this approach would be acceptable for implementation in routine clinical use with appropriate training and supervision. During the 12 month follow-up period, no differences in adverse events or serious adverse events or other harms were identified between the CBT plus standardised medical care and standardised medical care groups.

In conclusion, our model of CBT was not shown to confer a benefit for dissociative seizure frequency at 12 months when combined with specialist medical care involving careful explanation of the diagnosis and psychiatric care. Dissociative seizure-specific CBT was, however, associated with improvements in other secondary clinical outcome measures including psychosocial functioning, and our results indicate that dissociative seizure-specific CBT can be delivered safely to a wide range of patients with dissociative seizures.

## Data sharing

All data requests should be submitted to the corresponding author for consideration. Access to anonymised data might be granted following this, beginning 18 months and ending 36 months after Article publication.
