# Supplementary material for: Cognitive behavioural therapy for adults with dissociative seizures (CODES): a pragmatic, multicentre, randomised controlled trial
Source: Lancet Psychiatry. 2020 Jun;7(6):491–505. doi: 10.1016/S2215-0366(20)30128-0 (PMC7242906; doi:10.1016/S2215-0366(20)30128-0)
Supplement: Supplementary appendix [file mmc1.pdf]

THE LANCET
Psychiatry

Supplementary appendix

This appendix formed part of the original submission and has been peer reviewed. We post it as supplied by the authors.

Supplement to: Goldstein LH, Robinson EJ, Mellers JDC, et al. Cognitive behavioural therapy for adults with dissociative seizures (CODES): a pragmatic, multicentre, randomised controlled trial. *Lancet Psychiatry* 2020; **7:** 491–505.

Appendix for the CODES trial outcome paper

Supplement to Goldstein LH, Robinson EJ, Mellers JDC et al. Cognitive Behavioural Therapy for Adults with Dissociative Seizures: The CODES Randomised Controlled Trial.

Table of Contents

Descriptions of our interventions 4

Cognitive Behavioural Therapy (CBT) 4

SMC 4

Structure of DS-specific CBT intervention and suggestions of content for each session. 6

Protocol predating start of trial (dated 4 December 2013). 8

1. PROTOCOL FULL TITLE: COgnitive behavioural therapy vs standardised medical care for adults with Dissociative non Epileptic Seizures: A multicentre randomised controlled trial (CODES). 8

3. Study Synopsis 12

4. Revision History 15

5. Glossary of terms (Optional) 16

6. Protocol Contents (Table of contents removed from here) 17

7. Background & Rationale 17

8. Trial Objectives and Design 18

8.1 Trial Objectives 18

8.2 Trial Design 20

8.3 Therapy/Intervention Details 24

8.5 Intervention records 28

8.6 Subject Compliance. 29

8.7 Study adherence 29

8.8 Concomitant Medication 30

9. Research environment 30

10. Selection and Withdrawal of Subjects 30

10.1 Inclusion Criteria 34

10.2 Exclusion Criteria 34

10.3 Selection of Participants 35

10.4 Randomisation Procedure / Code Break 35

10.5 Withdrawal of Subjects 35

10.6 Expected Duration of Trial. 36

11. Trial Procedures 37

11.1 By Visit 37

12. Assessment of Safety 39
12.1 Specification, Timing and Recording of Safety Parameters 39
12.2 Procedures for Recording and Reporting Adverse Events 39
12.3 Stopping Rules 41
13. Statistics 41
13.1 Sample Size 41
13.2 Randomisation 42
13.3 Analysis 42
14. Trial Steering Committee 44
15. Data Monitoring Committee 44
16. Direct Access to Source Data and Documents 45
17. Ethics & Regulatory Approvals 45
18. Quality Assurance 45
19. Data Handling 46
20. Data Management 46
21. Publication Policy 46
22. Insurance / Indemnity 47
23. Financial Aspects 47
24. Signatures 47
25. References 49
Changes to protocol and registration 54
Table 1: Number (%) of participants recruited at phase 1 (screening) consent and phase 2 (trial) consent per neurology site 55
Table 2: Number (%) of participants recruited at phase 2 (trial) consent per psychiatry site*, by trial arm and overall 57
Table 3: Baseline demographic and clinical characteristics recorded after phase 1 (screening) consent of all participants who were eligible for phase 2 (N=426), split by whether they were randomised to the trial or not 58
Table 4: Other baseline demographics and clinical characteristics of the trial sample recorded after phase 2 (trial) consent (unless otherwise specified†) 60
Table 5: MINI-International Neuropsychiatric Interview (MINI) recorded after phase 2 (trial) consent 62
Table 6: Demographics of all clinicians seeing participants in the trial 65
Table 7: Participants' treatment experience 68
Table 8: Standardised fidelity rating scores across all 36 rated sessions (100 indicated optimal rating) 70
Table 9: Clinical Global Impression scores (self-, doctor- and therapist-rated), and treatment satisfaction scores at the relevant timepoints 71

Table 10: Estimated trial arm difference between CBT+SMC and SMC only at 12 months post randomisation using complete case analysis instead of multiple imputation 72

Table 11: Summary of all serious adverse events reported during the trial by body system 73

Table 12: Summary of all non-serious adverse events reported during the trial by body system....... 74

Detailed Description of Statistical Analysis... 75

Construction of the primary outcome 75

Details of the multiple imputation approach used to analyse primary and secondary outcome measures at 12 months 76

Table 13: Compliance with treatment vs. completion of 12-month monthly seizure frequency n (%)76

Table 14 Results of likelihood-ratio tests for each outcome comparing models with and without random effects for therapists in the CBT+SMC arm 78

Table 15 Variables included in the imputation step for the primary outcome 80

Extended discussion of SMC 82

Raw data plots to illustrate observed changes over time, by trial arm. 83

References for citations in the Appendix other than for the protocol document 91

CODES Study Group 92

Descriptions of our interventions

(For references see end of this document)

Cognitive Behavioural Therapy (CBT)

Our CBT intervention was based on that tested previously[1] and is described here and elsewhere.[2,3] Adapted specifically for patients with DS, the intervention was based on models of fear-avoidance[4,5] and views DS as states of altered awareness and responsiveness initially occurring in the context of heightened arousal[6] which leads to emotional and behavioural avoidance. DS have typically developed as a response to a range of internal (panic, syncope) or external (life events, trauma) triggers.[3] High rates of avoidance behaviour in response to arousal, fear of symptoms and DS themselves are common, sometimes inadvertently reinforced by others. CBT specifically focuses on reducing DS and arousal and emotional/behavioural avoidance, enabling people to re-engage in activities of everyday life. The intervention was structured to be delivered over 12 sessions (each approximately one hour long) over four-to-five months with a booster session occurring nine months post randomisation (see pages 6-7 for session outlines). The treatment was manualised, formulation-based, with suggested session content provided, allowing for flexibility in treatment delivery. A booklet of written handouts was given to patients to supplement session content. Therapists were encouraged, with patients' consent, to invite patients' significant others to certain therapy sessions. Therapy was delivered by cognitive behavioural psychotherapists (clinical psychologists, nurse therapists or other professionals allied to medicine), all of whom were trained in CBT and who received specific training in delivering CBT for DS via a three-day workshop or where necessary, individualised training. They also received four-to-six weekly group supervision and where appropriate individual supervision, from therapists very experienced in applying the therapy model and in working with this patient group. Therapists kept a record of sessions attended and other details such as whether patients' DS disrupted the sessions, the extent to which homework tasks were completed and the extent to which participants adhered to the therapeutic model. They also rated patients' improvement using the Clinical Global Impression change score after the 12th session. Therapy sessions were recorded with patients' consent and uploaded to a secure online facility for supervision and evaluation of treatment fidelity.

SMC

All participants received standardised medical care (SMC). This was based on a similar model of DS occurrence to that underpinning the CBT approach used here. SMC was delivered by neurologists and psychiatrists and involved delivery of the diagnosis as well as ongoing management. Neurologists and psychiatrists were expected to provide each patient with the relevant information booklet about DS (Dissociative Seizures Factsheet (Neurology) or Dissociative Seizures Factsheet (Psychiatry) (http://www.codestrial.org/information-booklets/4579871164) and were provided with guidelines and supporting materials to aid in the delivery of the diagnosis and subsequent follow-up. Neurologists' delivery of SMC commenced with delivery of the diagnosis and subsequent management was intended to provide review, supervision of AED withdrawal where appropriate, management of concomitant physical disorders, any psychopharmacological management of

anxiety/depression before the patient was seen by a psychiatrist and any completion of reports on patients e.g., regarding driving, as required. Psychiatrists' provision of SMC began with a clinical psychiatric assessment of patients approximately three months after diagnosis by neurologists. Psychiatrists were instructed to provide support, pharmacological treatment of comorbidities and provide patients with a general review but were asked to refrain from implementing CBT techniques for DS.

In both cases referral for crisis management psychiatric care could be made if necessary; referral for other psychotherapy (including CBT) was not to be made. We did not prescribe the exact number of SMC sessions to be provided but expected there to be around two neurology SMC sessions and three-to four SMC sessions provided by psychiatrists.

Structure of DS-specific CBT intervention and suggestions of content for each session.

Note, however, the treatment is formulation-based so treatment elements may be dealt with in a different order or may not be relevant.

**Stage**	**Session number**	**Content**
Stage 1: Engagement	1	• Assess main problem, its development, family history etc.<br>• Briefly discuss DS-specific model and CBT<br>• Homework: complete self-monitoring seizure diary<br>Give patient the booklet of supporting handouts entitled "Manual for Patients Attending CBT"
	2	• Review seizure diaries and homework as previously agreed and discuss difficulties<br>• Clarify treatment rationale<br>• Agree long term targets<br>• Introduce idea of distraction techniques<br>• Agree 2 realistic and achievable goals<br>Homework tasks – Self-monitoring of seizures and two goals: 1. Practice technique for interrupting seizures 2. Something addressing patient's avoidance behaviour
Stage 2: Active Treatment	3	• Review seizure diaries and homework as previously agreed and discuss difficulties<br>• Meet with family to discuss how factors in person life may be perpetuating patient's problems<br>• Discuss re-focusing and distraction techniques if patient can identify warning signs for seizure<br>• Homework: discuss distraction and refocusing techniques to be used in response to seizure warnings and a graded exposure goal.
	4	• Review seizure diaries and homework as previously agreed and discuss difficulties<br>• Identify past or present trauma or problems that may be contributing to seizure occurrence<br>• Introduce relaxation and breathing techniques to be used to interrupt the seizure, as a stress management technique or to manage anxiety<br>• Introduce how to recognise negative thinking styles<br>• Homework: apply relaxation and breathing techniques to seizure warnings. Continue with distraction and refocusing techniques and exposure to avoided situations
	5	• Review seizure diaries and homework as previously agreed and discuss difficulties<br>• Clarify patient's thinking errors e.g. overgeneralising, catastrophising, all-or-nothing thinking etc.<br>• Review avoidant behaviour and agree more goals<br>• Consolidate distraction, refocusing, relaxation and breathing techniques<br>• Introduce problem solving strategies<br>• Homework: identify unhelpful thoughts, using thought diaries as well as exposure
	6 Mid-treatment review	• Review seizure diaries and homework as previously agreed and discuss difficulties<br>• Review progress to date<br>• Identify and agree on remaining areas to be addressed<br>• Meet with family to evaluate progress to date<br>• Homework: negotiate goals as deemed appropriate
	7-9	• Review seizure diaries and homework as previously agreed and discuss difficulties<br>• Continue with goal setting

		• Continue to discuss negative thoughts and how to tackle them<br>• Continue to discuss the role of avoidance both behavioural and cognitive<br>• Continue to use distraction and refocussing techniques<br>• Encourage to use problem solving strategies, for both actual and potential problems<br>• Facilitate emotional processing if necessary<br>• Homework: agree goals
Stage 3: Preparation for Discharge	10	• Review seizure diaries and homework as previously agreed and discuss difficulties<br>• Identify potential or ongoing problems and how they might be addressed using CBT techniques following discharge from active treatment<br>• Homework: identify potential difficulties and address these using problem solving techniques
	11	• Review seizure diaries and homework as previously agreed and discuss difficulties<br>• Review treatment to date<br>• Allow time for reflecting on treatment process<br>• Discuss remaining issues<br>• Homework: as appropriate
	12	• Review of gains made within treatment<br>• Identify goals to be reviewed at booster session<br>• Emphasise that booster session is part of treatment package so as to encourage patient to attend<br>• Reinforce positively gains made within treatment and how they were made<br>• Meet with family to review progress to date<br>• Agree date for booster session<br>• Complete CGI rating on patient
Stage 4	13 Follow up (Booster session)	

Protocol predating start of trial (dated 4 December 2013).

N. B. See Robinson et al[7] for the published update to the protocol.

1. **PROTOCOL FULL TITLE: COgnitive behavioural therapy vs standardised medical care for adults with Dissociative non Epileptic Seizures: A multicentre randomised controlled trial (CODES).**

2. **Protocol Short Title/Acronym: Comparing different treatments in reducing dissociative seizure occurrence: A randomised controlled trial. (CODES)**

Trial Identifiers

**ISRCTN:**	TBC		
**REC Number:**	13/LO/1595		
**UKCRN Number:**	TBC		
**Protocol Version Number:**	2.0	Date:	4 December 2013

(Co) Sponsor(s)

**Name:**	**King's College London**
**Address:**	**Mr Keith Brennan, Director of Research Management, Director of Administration (Health Schools) Room 1.8, Hodgkin Building, Guy's Campus, London SE1 4UL**
**Telephone:**	**0207848 6960**
**Fax:**	
**Email:**	**Keith.brennan@kcl.ac.uk**

**Name:**	**South London & Maudsley NHS Foundation Trust**
**Address:**	**Ms Jennifer Liebscher, Joint SLaM / IoP R&D Office, Institute of Psychiatry, De Crespigny Park, London SE5 8AF**
**Telephone:**	**02078480251**
**Fax:**	
**Email:**	**Jennifer.liebscher@kcl.ac.uk**

Chief Investigator

**Name:**	Prof Laura H. Goldstein
**Address:**	Dept of Psychology, PO77, Henry Wellcome Building<br>Institute of Psychiatry<br>De Crespigny Park<br>London<br>SE5 8AF
**Telephone:**	020 7848 0218
**Fax:**	020 7848 5006
**Email:**	Laura.goldstein@kcl.ac.uk

Name and address of Co-Investigator(s), Statistician, Therapy Service, Laboratories etc

**Name:**	Prof Trudie Chalder
**Position/ Role:**	Professor of Cognitive Behavioural Psychotherapy/ Co-investigator
**Address:**	Department of Psychological Medicine<br>Institute of Psychiatry, De Crespigny Park<br>London<br>SE5 8AF
**Telephone:**	020 7848 0460
**Fax:**	
**Email:**	Trudie.chalder@kcl.ac.uk
**Name:**	Dr John D C Mellers
**Position/ Role:**	Consultant Neuropsychiatrist/ Co-investigator
**Address:**	Dept of Neuropsychiatry, Maudsley Hospital, Denmark Hill, London SE5 8AZ
**Telephone:**	020 3228 2330
**Fax:**	020 7919 2012
**Email:**	John.mellers@slam.nhs.uk

**Name:**	Prof Mark Richardson
**Position/ Role:**	Paul Getty III Professor of Epilepsy/ Co-investigator
**Address:**	Department of Clinical Neuroscience, PO43, Institute of Psychiatry<br>De Crespigny Park<br>London SE5 8AF
**Telephone:**	
**Fax:**	
**Email:**	Mark.richardson@kcl.ac.uk
**Name:**	Prof Sabine Landau
**Position/ Role:**	Professor of Biostatistics/Co-investigator
**Address:**	Department of Biostatistics, PO20, Institute of Psychiatry<br>De Crespigny Park<br>London SE5 8AF
**Telephone:**	0207848 0313
**Fax:**	
**Email:**	sabine.landau@kcl.ac.uk
**Name:**	Prof Paul McCone
**Position/ Role:**	Professor of Health Economics/Co-investigator
**Address:**	Health Service and Population Research, PO20, Institute of Psychiatry<br>De Crespigny Park<br>London<br>SE5 8AF
**Telephone:**	020 7848 0874
**Fax:**	
**Email:**	Paul.mccrone@kcl.ac.uk
**Name:**	Ms Joanna Murray
**Position/ Role:**	Senior Lecturer/ Co-investigator
**Address:**	Health Service and Population Research, PO20, Institute of Psychiatry

	De Crespigny Park<br>London<br>SE5 8AF
**Telephone:**	020 7848 0025
**Fax:**	
**Email:**	Joanna.murray@kcl.ac.uk
**Name:**	Prof Markus Reuber
**Position/ Role:**	Professor of Clinical Neurology/ Co-investigator
**Address:**	University of Sheffield<br>Academic Neurology Unit, Royal Hallamshire Hospital,<br>Glossop Road<br>Sheffield<br>S10 2JF
**Telephone:**	0114 2268763
**Fax:**	01142713158
**Email:**	markus.reuber@sth.nhs.uk
**Name:**	Dr Nick Medford
**Position/ Role:**	Senior Lecturer and Consultant Neuropsychiatrist/ Co-investigator
**Address:**	Brighton and Sussex Medical School<br>Neuropsychiatry Administration Office,<br>Princess Royal Hospital,<br>Lewes Road<br>Haywards Heath<br>RH16 4EX
**Telephone:**	01444 441881 ext 8515
**Fax:**	
**Email:**	n.medford@bsms.ac.uk
**Name:**	Dr Jon Stone
**Position/ Role:**	Hon Senior Lecturer and Consultant Neurologist/Co-investigator

**Address:**	University of Edinburgh, Dept of Clinical Neurosciences<br>Western General Hospital<br>Edinburgh<br>EX4 2XU
**Telephone:**	0131 537 1167
**Fax:**	
**Email:**	Jon.Stone@ed.ac.uk
**Name:**	Dr Alan Carson
**Position/ Role:**	Consultant Neuropsychiatrist/Co-investigator
**Address:**	Robert Fergusson Unit, Royal Edinburgh Hospital<br>Morningside Terrace<br>Edinburgh
**Telephone:**	0131 537 6896 (6383)
**Fax:**	
**Email:**	alan.carson@nhslothian.scot.nhs.uk

**Name:**	Nicholas Magill
**Position/ Role:**	Trial Statistician
**Address:**	King's Clinical Trials Unit, Department of Biostatistics, Institute of Psychiatry, De Crespigny park, London SE5 8AF
**Telephone:**	020 7848 0323
**Fax:**	
**Email:**	Nicholas.magill@kcl.ac.uk

3. Study Synopsis

**TITLE OF CLINICAL TRIAL:**	COGNITIVE BEHAVIOURAL THERAPY VS STANDARDISED MEDICAL CARE FOR ADULTS WITH DISSOCIATIVE NONEPILEPTIC SEIZURES: A MULTICENTRE RANDOMISED CONTROLLED TRIAL (CODES).
**Protocol Short Title/ Acronym:**	CODES

**Study Phase If Not Mentioned In Title:**	Phase 3
**Sponsor Name:**	King's College London and South London and Maudsley NHS Foundation Trust
**Chief Investigator:**	Prof Laura H. Goldstein
**UKCRN Number:**	Tbc
**REC Number:**	13/LO/1595
**Medical Condition Or Disease Under Investigation:**	Dissociative seizures
**Purpose Of Clinical Trial:**	Pragmatic randomised controlled trial to evaluate the effectiveness and cost effectiveness of cognitive behavioural therapy to reduce seizure frequency and severity and improve psychological well-being in adults with dissociative seizures
**Primary Objective:**	The primary objective is to evaluate the effectiveness of CBT (plus SMC) compared to SMC alone in reducing DS frequency (our primary outcome) at 12 months post randomisation
**Secondary Objective(s):**	The secondary objectives are to evaluate:<br>1) the effectiveness of CBT plus SMC compared to SMC alone in reducing DS severity and promoting seizure freedom, quality of life and psychosocial well-being at 12 months post randomisation;<br>2) the effectiveness of CBT plus SMC compared to SMC alone in reducing disability and reducing health service use and informal care costs at 12 months post randomisation;<br>3) the cost-effectiveness of CBT plus SMC compared to SMC alone at 12 months post randomisation;<br>4) clinical global change as a result of treatment from the patient's and clinician's perspective and satisfaction with treatment from the patients' perspective<br>5) DS patients' subjective experiences of CBT vs. SMC from in depth interviews;<br>6) treatment fidelity of our manualised DS-specific CBT treatment across different therapists.
**Trial Design:**	Parallel group multi-centre randomised controlled trial comparing cognitive behavioural therapy in addition to standardised medical care vs. standardised medical care alone
**Endpoints:**	Change in seizure frequency and secondary clinical outcomes as well as health and societal cost effectiveness at 6 and 12 months after randomisation
**Sample Size:**	Randomisation of 298 people with dissociative seizures

**Summary Of Eligibility Criteria:**	*Our inclusion criteria applied at the initial recruitment stage will be as follows:*<br>• adults (>18yrs) with DS confirmed by video EEG telemetry or, where not achievable, clinical consensus; patients who have chronic DS can be included if they have been seen by the relevant Study Neurologist who has reviewed their diagnosis and communicated this to them according to the Study protocol;<br>• ability to complete seizure diaries and questionnaires;<br>• willingness to complete seizure diaries regularly and undergo psychiatric assessment 3 months after DS diagnosis;<br>• no documented history of intellectual disabilities;<br>• ability to give written informed consent.<br><br>*Inclusion criteria evaluated at the randomisation stage will be as follows:*<br>• adults (>18yrs) with DS initially recruited at point of diagnosis;<br>• willingness to continue to complete seizure diaries and questionnaires;<br>• having provided regular seizure frequency data to research team following receipt of DS diagnosis;<br>• willingness to attend weekly/fortnightly sessions if randomised to CBT<br>• both clinician and patient agree that randomisation is acceptable<br>• ability to give written informed consent;<br><br>***Exclusion Criteria***<br>*Our exclusion criteria applied at the initial recruitment stage will be as follows:*<br>• having a diagnosis of current epileptic seizures as well as DS;<br>• inability to keep seizure records or complete questionnaires independently;<br>• meeting DSM-IV criteria for current drug/alcohol dependence;<br>• insufficient command of English to later undergo CBT without an interpreter or to complete questionnaires independently<br><br>*Exclusion criteria evaluated at the randomisation stage will be as follows:*<br>• current epileptic seizures as well as DS, for reasons

	given above;<br>• not having had any DS in the 8 weeks prior to the psychiatric assessment, 3 months post diagnosis;<br>• having previously undergone a CBT-based treatment for dissociative seizures at a trial participating centre;<br>• currently having CBT for another disorder<br>• active psychosis;<br>• meeting DSM-IV criteria for current drug/alcohol dependence; this may exacerbate symptoms/alter psychiatric state and health service use and affect recording of seizures;<br>• current benzodiazepine use exceeding the equivalent of 10mg diazepam/day;<br>• the patient is thought to be at imminent risk of self-harm, after psychiatric assessment and structured psychiatric assessment by the Research Worker with the MINI.<br>• Known diagnosis of Factitious Disorder
**Intervention (Description, frequency, details of delivery)**	Cognitive behavioural therapy (in addition to standardised medical care-see below). CBT therapists (health professionals, i.e. clinical psychologists/nurse therapists or other professions allied to medicine-already trained in CBT) who have undergone further specific training for the trial will deliver 12 sessions of CBT +1 booster session. Therapy will be informed by a therapy manual and patient handouts and will involve setting homework tasks.
**Comparator Intervention:**	Neurologists and psychiatrists with an interest in DS will deliver standardised medical care. They will have guidelines as to the delivery of standardised medical care. Information leaflets will be given to the patients. The research team will provide this material.
**Maximum Duration Of Treatment Of A Subject:**	CBT will take place over 4 to 5 months with a further booster session approximately 9 months after randomisation.
**Version And Date Of Final Protocol:**	
**Version And Date Of Protocol Amendments:**	Version 2: December 2013

4. Revision History

**Document ID - (Document Title) revision**	**Description of changes from previous revision**	**Effective Date**
Document1	New Protocol	September 2013

Document 2	Changes to inclusion and exclusion criteria and recategorisation of measures	December 2013

5. Glossary of terms (Optional)

DS = dissociative non-epileptic seizures;

CBT= Cognitive Behavioural Therapy;

SMC= standardised medical care;

MUS= medically unexplained symptoms;

QALYs= quality-adjusted life years;

CLRN=Comprehensive Local Research Network;

IAPT= Increased Access to Psychological Therapies; RCT= Randomised Controlled Trial RW= Research Worker;

fte=full time equivalent;

CI= Chief Investigator.

6. Protocol Contents (Table of contents removed from here)

7. Background & Rationale

Overview:

We propose a pragmatic RCT to evaluate the clinical and cost-effectiveness of specifically modified CBT (plus SMC) versus SMC alone in the treatment of patients with DS. We will recruit and consent patients without current comorbid epilepsy at point of diagnosis by neurologists. They will consent to data collection and review by a psychiatrist after three months. Following this psychiatric review (by a liaison/neuropsychiatrist or other psychiatrist with interest in DS at 3 months, those who have continued to have DS in the previous 8 weeks will be consented to take part in the RCT, undergo a structured psychiatric assessment to identify comorbidities and collection of baseline measures and then randomised to receive either manualised CBT (12 sessions over 4-5 months) plus SMC or SMC alone. Allowing for loss to follow-up, clustering for therapist effects, correcting for prerandomisation DS frequency and a moderate effect size, we aim to randomise 298 patients in total. Follow-up will be at 6 and 12 months post randomisation with outcomes evaluated at 12 months. Data will be analysed using an intention to treat principle. Our primary outcome will be monthly seizure frequency. Secondary outcomes will be informants' ratings of patients' DS frequency, patients' self-rated seizure severity, numbers showing >50% reduction in seizures, maximum duration of seizure freedom in the last 6 months of the study and whether or not patients are seizure free in months 9-12 post randomisation; quality of life, psychosocial functioning, anxiety, depression and somatisation, and work/disability status. Healthcare use, costs, societal costs and cost-effectiveness will also be assessed. Findings will be relevant to DS patients and those who plan and deliver their care.

Background and rationale

DS are paroxysmal events, which superficially resemble epileptic seizures but lack any organic basis. They are a common cause of Transient Loss of Consciousness[1]. Approximately 12-20% of patients seen in epilepsy clinics may have DS[2] and such patients present a diagnostic and management challenge. Recent incidence estimates are 4.9/100,000/year[3]. Patients may previously have been misdiagnosed and treated for epilepsy; arrival at the correct diagnosis may take many years[4]. Long-term outcome (chronic disability and welfare dependence) in one study was noted to be poor in about 70% of patients[5]. The vast majority of DS patients are thought to have symptoms that are not deliberately generated. Therefore they would receive diagnoses of somatoform, conversion or dissociative disorder under current classification systems (ICD-10[6]; DSM-IV[7]). They demonstrate high rates of psychiatric comorbidity (e.g. other somatoform / conversion symptoms, anxiety, depression, accentuation of maladaptive personality traits and posttraumatic stress disorder) (e.g.[8]). DS patients also have a slightly raised risk of mortality[9]. There is renewed concern that, in the UK, patients with mental health needs are receiving inadequate service commissioning and insufficient treatment, despite the potential for recovery and reduction in NHS costs[10]. Patients with DS may undergo unnecessary, costly and potentially harmful tests and interventions and may sustain injuries during their seizures. Quality of life is lower than in epilepsy patients (e.g.[11]) and correlates with depression and somatic symptoms. Patients' lifestyles can be severely restricted through fear of having seizures, with high levels of avoidance behaviour[12]. Patients may be taking anti-epileptic drugs (AEDs) unnecessarily, with associated risks for women of childbearing age. A US study[13] evaluated the six-year cost pre-diagnosis as in excess of $25,000/patient and US lifetime costs at $110-920m. After correct diagnosis, a reduction in medical service use with attendant cost reductions may follow (e.g.[14]). Patients with DS are also vulnerable to other MUS such as chronic pain or other functional neurological symptoms[15].

Despite limited evidence to date for its effectiveness[16-18], psychotherapy is viewed as the treatment of choice[19]. Evidence for the efficacy of psychotherapy for patients with DS has come from a number of small uncontrolled studies and pilot RCTs which, in particular, suggest the potential effectiveness of CBT. Our group's manualised CBT treatment for DS has, in a recent pilot RCT[20], shown the potential to reduce DS frequency compared to SMC. Both treatments led to some improvement in psychosocial functioning. Adequately powered studies are now required to underpin evidence-based care pathways for DS patients, which currently do not exist. There is variable involvement of psychiatrists and psychologists in the assessment of patients, clarification of the diagnosis and management. A survey of UK healthcare professionals working with DS patients indicated considerable variability in the extent to which patients might be referred on for psychological therapy. and in clinicians' knowledge of what type(s) of psychotherapy might be available and where[19]. In that study, only one third of respondents indicated that they could refer all their patients for psychotherapy; 15% were unable to refer any of their patients. One third of respondents indicated that fewer than half their patients would be offered >1 psychotherapy session.

Poor economic activity / dependence on state benefits at diagnosis/the beginning of treatment predicts poor outcome[21], [22,], so early identification and treatment may improve outcome and reduce health service costs. Our study will, therefore, permit evaluation of the clinical and cost effectiveness of CBT for DS within a structured care pathway involving neurology, (neuro) psychiatry and psychotherapy and should then provide a model for future services and more rational commissioning of care

for this patient group. It will provide a basis for the wider training of therapists to work with DS patients and support the role of psychiatrists in treating this group of patients, who commonly have complex mental health care needs[23].

Interest in this patient group has grown over the past decade. NICE[24] and the Scottish Intercollegiate Guidelines Network (SIGN)[25] have recognised the need for psychiatric and psychological input for DS patients. The International League Against Epilepsy (ILAE) has produced consensus guidelines for managing DS[26], and there is an ILAE Task force on DS, of which the current Chief Investigator and Professor Reuber have been members. In 2005, the US National Institutes of Health and the National Institute for Neurological Disorders and Stroke (NINDS), together with the American Epilepsy Society ran a workshop on treatments for DS[27], attended by the current PI. The NINDS included the development of treatments for DS in its research benchmarks[28] and called for treatment-related DS research. More locally, the UK Epilepsy Research Network (UKERN) Interventions and Therapies Clinical Study Group has recently called for the development of effective methods for the treatment of DS. Health Improvement Scotland (NHS Scotland) also called for the provision of specific services for patients with DS.

Thus, national and international organisations have recognised the need for research into

treatment in this area. A number of reviews, including by us[16, 17], have discussed the increased interest and reporting of treatment studies, although these have largely been open label or small controlled studies. While there is increasing acknowledgement of the interface between neurology and psychiatry, neuropsychiatry care pathways are relatively under-developed[23]. This is despite increased recognition that neuropsychiatric disorders, such as DS, may cause distress to patients and their carers, disability, burden and loss of productivity[23]. However, care provision for DS patients in the UK is extremely variable, with currently no rational basis on which to decide the type of psychotherapy that should be recommended for this patient group. In contrast, UK care pathways have been developing more generally for patients with MUS[29]. A strong evidence base would allow policy changes to the service provision for DS patients within such frameworks.

Despite evidence from a small pilot RCT of CBT for DS by our group[20], the lack of an evidence-based care pathway and of larger systematic treatment trials means that many patients may not have been funded by their Primary Care Trusts to receive any psychotherapy, despite the likely high rates of psychiatric comorbidity. In addition, the geographic distribution of these patients means that outside of specialist centres, there may be limited knowledge or willingness to enable these patients to be seen. The limited evidence base is leading to local variations in what may be provided, resulting in marked inequalities in health care provision for these patients. Given that commissioning arrangements for neuropsychiatry services in the UK are changing, alongside general changes in commissioning procedures, it will be crucial for rigorous treatment studies to influence the nature of care provided.

Following MRC Guidelines for complex interventions[30], we have completed a proof of principle RCT and obtained preliminary evidence of efficacy[20]. We have tested patients' willingness to be randomised and undertaken randomisation, prepared a manual andhandouts for patients, devised and used measures of treatment fidelity and supervisedtherapists. Thus, the next logical step is to test clinical and cost effectiveness and generalisability in an adequately powered, multi-centre RCT.

8. Trial Objectives and Design

8.1 *Trial Objectives*

Our overall aim is to evaluate the clinical and cost effectiveness of specifically adapted CBT (plus SMC) in comparison to SMC alone for outpatients with DS, within a pragmatic, multicentre RCT.

8.1.1 *Primary endpoints*

Our primary objective is to evaluate the effectiveness of CBT (plus SMC) compared to SMC alone in reducing DS frequency (our primary outcome) at 12 months post randomisation;

Our primary outcome measure is monthly DS frequency. This is a continuous variable that comprises a count of seizures over a month and therefore will reflect all participants' outcomes, whether they improve or not during the study. Seizure frequency has been used as an outcome measure in other studies of psychological interventions for DS (e.g.[20, 313219]). This will be recorded by patients in seizure diaries (paper or electronic as preferred), as has been done in other studies of psychotherapy for DS. We will collect seizure frequency data from the patients themselves every 2 weeks by whichever means they find acceptable (diaries, phone/online) to reduce recall bias and missing data.

8.1.2 *Secondary endpoints*

Our secondary objectives are to evaluate:

1) the effectiveness of CBT plus SMC compared to SMC alone in reducing DS severity and promoting seizure freedom

and quality of life, psychosocial and psychological well-being at 12 months post randomisation;

2) the effectiveness of CBT plus SMC compared to SMC alone in reducing disability

3) the effectiveness of CBT plus SMC compared to SMC alone in reducing health service use at 12 months post randomisation;

4) the cost-effectiveness of CBT plus SMC compared to SMC alone at 12 months post randomisation;

5) patients' global change as a result of treatment (Clinical Global Impression (CGI)[33] change score) and their satisfaction with this

6) DS patients' subjective experiences of CBT vs. SMC, determined from qualitative interviews;

7) the role of therapist effects when evaluating the benefit of therapy

8) the generalisability of our manualised DS-specific CBT treatment across different therapists (treatment fidelity) and determine the implications for rollout in the NHS.

To achieve Objectives 1-5, we will collect the following measures:

Outcomes that may be affected by CBT:

a) A rating by an informant as to whether, compared to study entry (i.e. time of diagnosis) the patient's seizure frequency is worse, the same, better or whether they are seizure free. For those randomised we will collect this data at the 6- and 12-month follow-up.

b) Self-rated seizure severity: no specific measures of seizure severity for DS exist, and even in the case of epilepsy, there is concern over which scales are valid[34-36]. We will use 2 items from the Seizure Severity Scale[34], asking how severe and bothersome DS were in the past month. In seizure diaries we will also ask patients to indicate how many seizures that they have had they would consider to have been severe.

c) Seizure freedom: we will record patients' self-reported longest period of seizure freedom between the 6 and 12-month follow-up and, in line with our previous study, whether or not the patient is seizure free in the last 3 months of the trial[20].

d) The number of patients in each group who at the 6- and 12-month follow-up show >50% reduction in seizure frequency, compared to baseline. This requires no further data collection but allows the calculation of a ‘number needed to treat' metric.

e) Quality of life (QoL): the validity of epilepsy-specific QoL measures for DS patients is unclear since epilepsy-specific items may not be relevant for DS patients[37]. Thus, we will use a generic measure of health-related QoL, the SF-12v2[38] to allow more direct comparison to be made with other disorders. This will also allow us to calculate QALYs, although the principal measure for doing that in this study is the EQ-5D-5L[39], a 5-domain, 5-level, multiattribute scale.

f) Psychosocial functioning: we will use the 5-item Work and Social Adjustment Scale (WASAS)[40] to measure patients' own perceptions of the impact of DS on their functioning in terms of work, home management, social leisure and private leisure activities, family and other relationships.

g) Psychiatric symptoms and psychological distress: we will measure anxiety, depression and somatisation with the GAD7[41], PHQ9[42] and an extended PHQ15[43, 44] derived from the Patient Health Questionnaire which reflects DSM-IV diagnoses. The GAD7[41] is a 7-item anxiety scale with good internal consistency (Cronbach's alpha= 0.92), test-retest reliability (intraclass correlation=0.83), sensitivity (89%), specificity (82%) criterion, construct and factorial validity. The PHQ9[42] is a 9-item depression scale that can be used to diagnose major depression (DSM-IV). It has good internal consistency (Cronbach's alpha=0.86-0.89) and test-retest reliability (r=0.84); sensitivity and specificity and construct validity are good.

The PHQ15[43] has been shown to have high internal validity (Cronbach's alpha=0.8) and strong convergent and discriminant validity; it has been used in DS research[45] and an extended version to include common neurological symptoms has been developed[44]. We will also use a general measure of psychological distress, the CORE-10[46]; this assesses selfreported global psychological distress, and places minimal demands on patients. Comprising items drawn from the CORE-OM[47], with which it correlates highly and which has been validated in large clinical and nonclinical samples, it has been used in a study of DS[45].

h) At the 6 and 12-month follow-ups, and in line with many CBT studies, we will measure patients' self-rated global outcome and satisfaction with treatment (2 questions). The Clinical Global Impression (CGI)[33] change score yields a self-rated global measure of change and has been used in previous trials of CBT interventions.

i) The CGI change scale will be rated by CBT therapists at end of session 12 and by SMC doctor at the 12-month follow-up.

j) Health service use (including hospital attendances and admissions, GP contacts), informal care, lost work time and financial benefits (which will be used as predictors of outcome in our analysis) will be measured via the self-report Client Service Receipt Inventory[49].

k) Objective measure of health service use; we seek to obtain linkage data sets from NHS Health and Social Care Information Centre (Hospital Episode Statistics) eDRIS (NHS National Services Scotland Information Services Division (ISD)) and Wales

(NHS Wales Informatics Service) to allow quantification of objective measures of hospital attendances and admissions pre-randomisation and during follow-up using ICD-10 codes.

To measure treatment process:

l) We will employ the 12-item Beliefs About Emotions Scale[48] (Cronbach's alpha=0.88 in

healthy controls);

m) we will use a single item scale for them to measure their confidence in the treatment they have received and a further item for them to rate their confidence in their diagnosis of DS.

n) We will also administer three locally-developed questions to measure avoidance of people places and activities due to DS.

To measure baseline variables that might modify the treatment effect:

o) We will employ an item asking participant to indicate their preference for treatment arm pre-randomisation. treatment preference might be explored as a moderator as might other baseline demographic and socio-economic variables collected as above.

To achieve Objective 6 we will undertake in-depth one-to-one interviews with ~20 patients randomised to the CBT group and ~ 10 randomised to SMC alone. A purposive sample will be identified to maximise variation in key characteristics, including gender, age, ethnicity and treatment adherence. RWs blind to the outcomes will conduct semi-structured in-depth interviews. Topics for the interview guide will be informed by the literature but with ample opportunity for participants to discuss their experiences of the trial in the context of their own beliefs about their condition and the trial's impact on their lives.

To achieve Objectives 7 and 8 we will rate 10% of audio-recorded CBT sessions to determine treatment fidelity across therapists. This will be done on the basis of rating scales already devised[1]. Two independent raters will listen to the audio-recordings and will rate the extent to which specific CBT skills are used and will rate aspects of the therapeutic alliance. To describe the therapists we will also collect data on the therapists' professional background, training and years spent delivering CBT.

8.2 *Trial Design*

Our design is a parallel group, two-arm, multi-centre pragmatic RCT testing the hypothesis that CBT plus SMC will have greater clinical and cost effectiveness than SMC alone in treating adult patients with DS which had not initially ceased following diagnosis. We will include a follow-up at 6 and 12 months post randomisation. Those randomised to SMC alone will be referred for psychotherapy at the end of the study if they are deemed to require this at the end of the study but no data will then be collected on these people and this treatment course will not form part of the clinical trial.

Flowchart

	Screen by neurologist	Phone call by research nurse/ Worker	Visit by Research nurse / worker	Review by psychiatrist	Phone call by research nurse/ worker	Baseline	End of CBT	6M post randomis-ation	12M post randomis-ation	With drawal	After data collection
Patient given PIS and leaflet	X			X							
Eligibility checklist to confirm eligibility		X			X						
Seizure frequency diaries			X	X		X		X	X		
Consent to first phase of study			X								
Consent to RCT						X					
Demographics			X			X					
MINI						X					
SAPAS-SR						X					
Seizure Severity						X		X	X		
Seizure Freedom				X		X			X		
Informant rating of seizures								X	X		
SF12-v2						X		X	X		
EQ-5D-5L						X		X	X		
WASAS						X		X	X		
CORE-10						X		X	X		
PHQ9						X		X	X		
GAD7						X		X	X		
PHQ-15						X		X	X		
CSRI						X		X	X		
Clinical Global								X	X		

Change (patient)											
Clinical Global Change (CBT Therapist)							X				
Clinical Global Change (SMC doctor)									X		
Beliefs About Emotions						X		X	X		
Avoidance of people places and activities						X		X	X		
Treatment preference						X					
Expectat-ion of treatment outcome						X					
Belief in diagnosis						X		X	X		
Belief in treatment						X		X	X		
Checklist for potential adverse events								X	X		
Ratings of treatment fidelity by independ-ent raters											X
HES data for specified time points requested									X		
Qualitative interviews (topic guide to be devised) to take place just after 12 follow up									X		

Satisfaction with treatment								X	X		
Therapy group											
Treatment attendance							X				
Therapist rating of patients' treatment compliance							X				
Details of therapists							X				
SMC delivery											
Details of doctor delivering SMC	X			X							
Other forms											
Randomis-ation form						X					
Research worker treatment guess										X	X
With drawal form											X
Status in study form									X	X	

Trial Intervention

8.3 *Therapy/Intervention Details*

Cognitive Behavioural Therapy for DS:

There is no single model of CBT for use by patients with DS, since CBT permits modification for specific groups according to the model of the disorder, despite containing core principles and techniques. Elements of CBT were present in a number of the approaches applied in an early case series[32]. However, the CBT approaches described in most detail in the literature are those used by our group and by LaFrance et al[50]. To date, the approach developed by LaFrance et al. has been evaluated in an open label study[50] and a very small multi-centre pilot RCT[51] Their CBT model is based on a programme initially derived to enhance self control of epileptic seizures[52] and is described as a Beckian approach predicated on the assumption that for patients with DS life experiences and trauma result in maladaptive core beliefs (negative schemas), leading to cognitive distortions and somatic symptoms.

Standardised Medical Care

We will compare the clinical and cost-effectiveness of CBT (plus SMC) to that of SMC alone.

While the provision of standard medical care to DS patients in the UK is variable[19], different specialities contribute to standard care in specific ways. Through the development of a protocol and the development of new as well as use of existing materials, we will establish key approaches to the delivery of what is best considered here as *standardised* medical care (SMC). This will contain elements documented elsewhere and shown to be achievable, and acceptable to patients[53]. This approach, involving the provision of a crib sheet, detailed leaflet for clinicians and briefing sessions for medical staff has been shown to work in such studies[53]. The key elements of SMC are described below.

Overall plan for delivery of SMC: Key components, delivery and training

SMC will be provided to study patients by neurologists and psychiatrists. We include SMC by psychiatrists as being an important stage in the patients' care pathway, given the apparent complexity of presentation/psychiatric history of many patients, the need to consider appropriate psychopharmacological management of some comorbidities, and the likelihood that the majority of neurological examinations will not have elicited all the background factors that may be relevant to the aetiology and maintenance of the individual's DS. In this study, psychiatrists' provision of SMC of patients begins post diagnosis.

Key components of SMC

We aim to achieve a common approach to SMC. This is to ensure that the neurologists and psychiatrists with whom an individual patient interacts in the study will be more likely to use the same kind of terminology about DS, the treatment and the study. This will help optimise recruitment and engagement in therapy. These are described below

Written Materials for Patients

For the patients we have drafted two leaflets

1. What are Dissociative Seizures? - This is a short leaflet for neurologists to share with patients. It will not contain any reference to the randomised controlled trial.

2. Dissociative Seizures - Information for Service Users. This is more detailed written information to supplement SMC psychiatry treatment. This will be shared with the patient at the time of the initial psychiatry assessment.

These leaflets are in draft form and will be refined further prior to the trial. We are currently in the process of consulting with service users as to their ease of comprehension and obtaining a review by the Patient Information Officer at the South London and Maudsley NHS Foundation Trust for reading ease.

Neurologists' delivery of SMC

Key points

The following will be considered to be mandatory elements to the neurologists' delivery of

SMC. They require the neurologist to:

- Make a robust diagnosis of DS;
- Give the patient a standardised introductory leaflet - ‘What are Dissociative Seizures?';
- Explain the diagnosis to the patient in simple terms;
- Refer the patient to the study psychiatrist.

The rest of the description represents recommended care.

Assessment and Diagnosis:

Neurologists will be expected to assess the patient in the usual way to determine the nature of the patient's seizures. In some cases it will be possible to make a secure diagnosis on the basis of the history, witness history and physical assessment. In other cases mobile phone footage, EEG or video EEG may be required to make the diagnosis. Where available and practical an EEG with concurrent video is the most reliable way to make the diagnosis but it is not mandatory. Likewise, neuroimaging is not mandatory and it is anticipated that this will be carried out only according to clinical need.

As part of standard history taking the neurologist will have determined the nature of any physical comorbidities, including other physical symptoms that may not be related to a disease process.

Neurologists will not be expected to carry out a standardised psychiatric assessment. However, as with all patients attending a neurological service, if there are clearly recognisable psychiatric risks related to self-harm, harm of others or psychosis the neurologist will refer to the relevant psychiatric services, or ask the patient's GP to do so.

This referral may be to a specialist neuropsychiatry service, liaison psychiatry or it may be to a local general psychiatry service, depending on local service configuration, the nature of the psychiatric problem, and the urgency (for example if a patient is felt to be at high risk of harming themselves in the near future it would be more appropriate to refer to a local crisis team than to a specialist neuropsychiatry service).

Explanation of the Diagnosis

Neurologists will have a key role in delivering the initial diagnosis of DS.

Provision of written material

All neurologists will be expected to give patients a copy of an information leaflet about Dissociative Seizures - ‘What are Dissociative Seizures?' This will include many of the points below and direction to self-help information (e.g. www.nonepilepticattacks.info, www.neurosymptoms.org, the NEAD Trust websites).

Verbal Explanation

We will also recommend that neurologists deliver a verbal explanation of the main components of the explanation as follows:

1) Explain the disorder:

i) Give a diagnostic label - using any of the terms ‘non-epileptic attacks/seizures' ‘dissociative attacks/seizures';

ii) Explain what the patients do not have (epilepsy) and why (explanation of diagnosis, drawing particular attention to positive aspects of the diagnosis and a restatement of why tests are confirmatory of the diagnosis);

iii) Explain that their attacks are considered genuine and they are not suspected of “putting on” or ‘imagining' the attacks;

iv) Explain that the disorder is common;

v) Explain that the condition is potentially reversible.

2) Explain the mechanism

We allow for the possibility that neurologists may wish to talk about the mechanism of the attacks being a ‘trance-like' state called dissociation, similar to that seen in hypnosis.

Individual explanations will vary according to the patient's presentation. We will advise the neurologist against using any explanation that leaves the patient thinking that the doctor does not believe them or thinks they are just ‘making it up'.

3) Explain reason for referral to psychiatrist

The neurologist will need to explain why they are referring the patient to see a psychiatrist.

Some suggestions for things that the neurologist can emphasise to the patient in discussing the referral to a psychiatrist are

- The doctor may be a psychiatrist but they will not think the patient is “crazy”;
- The psychiatrist knows about DS and has successfully helped other patients with the

problem;

- Psycho-social factors are often important in understanding DS. Part of the reason for referral is to explore this further;
- To assess factors that might be maintaining the attacks. For example, many patients with DS become concerned about leaving the house in case they have an attack. Worry about leaving the house may increase seizures.

Further aspects of the initial neurological consultation

These will vary according to the patient but could involve:

i) Explaining that antiepileptic drugs do not help DS, can have serious long-term side effects and should be withdrawn gradually;

ii) Explaining that talking treatments (including cognitive behavioural therapy) may be helpful for some people but the evidence is currently uncertain as to whether it is worthwhile;
iii) Providing explanations to family and friends about the diagnosis, and what to do when the patient has an attack;

iv) Providing general information about distraction techniques. The psychiatrist may introduce these;

v) Discussing DVLA regulations.

Further neurology follow up

We will recommend at least one further neurology follow-up visit (although fewer or more are allowable) which may typically cover the following topics:
i) Overall general review of progress;
ii) Checking the patient's understanding of the diagnosis and explaining again if necessary;

iii) Supervision of AED withdrawal;

iv) Management of any comorbid physical conditions - e.g. referral to physiotherapy for a rehabilitative approach towards poor mobility, fatigue or pain management, referral to appropriate specialist for other symptoms (e.g. urology for bladder problems);

v) Reassessment for major psychiatric risk such as self-harm or psychosis

vi) Recommendations for antidepressant or anti-anxiety medication prior to first visit with psychiatrist if clinically indicated

vii) Completion of forms from DVLA or Department of Work and Pensions if requested by those agencies

Psychiatrists' delivery of SMC

Again, as with the neurologists, we will work with recruiting psychiatrists to achieve a common approach to SMC, and so that the neurologists and psychiatrists with whom an individual patient interacts in the study will be more likely to use the same kind of terminology about DS, the treatment and the study. This will help optimise recruitment and engagement in therapy.

Psychiatrists' provision of SMC of patients begins after diagnosis with an outpatient

appointment within 3 months of the neurological assessment.

The initial pre-randomisation clinical psychiatric assessment will include the following components and partly have a psychoeducational function:

i) Reiteration of all of the points covered by the neurologist at diagnosis including checking the patient has received the information leaflet from the study that was delivered by the Neurologist and direction to self help information;

ii) Provision of a more detailed leaflet “Dissociative Seizures- Information for Service Users”;

iii) Acknowledge fears about a psychiatric label;

iv) Clinical assessment of relevant axis 1 (e.g. depression, anxiety) and axis 2

(personality disorder traits) psychiatric disorders including an assessment of the risk of self- harm/suicide;

v) Explanation and treatment of any psychiatric comorbidity. This may include provision of psychopharmacological treatment (for example antidepressants) or general treatment as required;

vi) Explanation of any other functional somatic symptoms, general advice about management and referral to physiotherapy if appropriate for mobility problems;

vii) Discussion of factors emerging from the clinical history that seem to have aetiological significance: relevance of predisposing, precipitating and perpetuating factors in their case if apparent;

viii) General information provision about any warning symptoms and distraction but not specific techniques and not discussed repeatedly so that this does not become therapy;

ix) Liaison with other mental health professionals involved in the patient's case as appropriate but no referral for other psychotherapeutic input (including use of CBT

techniques) specifically for DS. The emphasis should be on psycho-education and management of comorbid psychiatric conditions in the normal way;

x) Involvement of family or friends in the above steps as required;

xi) Encouragement in social activities, return to college/work as appropriate. This may require liaison with work / school / college to explain disorder and assist in correct management of DS in these environments if appropriate;

xii) Completion of forms from DVLA or Department of Work and Pensions if requested by those agencies.

Further SMC by psychiatrists will include support, consideration of psychiatric comorbidities and any associated drug treatment and general review but no CBT techniques for DS.

8.4 *Frequency and duration of intervention*

How the CBT will be delivered:

CBT will be delivered over 12 sessions (each approximately one hour in length) over a 4-5 month period with one booster session at 9 months post randomisation.

Our CBT model:

Our model has been developed from a single case study[54], trialled in an open label study[55]

and then in a Pilot RCT[20]. Thus, based on our Pilot RCT[20] we will assess a 12-session (plus one booster session) package of CBT specifically modified for treating DS. This has been described[56] and tested by our group[20, 55] providing proof of principle and primary evidence of efficacy to underpin this effectiveness trial.

The model is based on the two-process fear escape-avoidance model[57] and conceptualises DS as dissociative responses to cues (cognitive/emotional/physiological or environmental) that may (but not in all cases) have been associated with profoundly distressing or life- threatening experiences, such as abuse or trauma, at an earlier stage in the person's life and which have previously produced intolerable feelings of fear and distress[56]. There are essentially five stages to the treatment; engagement and rationale giving; teaching and use of seizure control techniques; reducing avoidance exposure technique; dealing with seizure- related cognitions and emotions; and relapse prevention. Thus, treatment includes helping the patient to:

- Develop a more coherent understanding of their DS
- develop an understanding of the interrelationship between cognitive, emotional, physiological and behavioural aspects of their DS
- understand factors maintaining the DS occurrence
- learn how to interrupt the behavioural, cognitive or physiological responses occurring prior to or at the start of the seizures
- engage in previously avoided activities, address negative thoughts and illness attributions maintaining seizures, and
- deal with previous traumatic experiences, anxiety, low mood or low self-esteem if present.

Treatment components draw on evidence-based practice. Sessions include typical CBT techniques (the setting of session agendas, the review of previous and planning of subsequent homework activities) and here, importantly, completion of seizure diaries. The treatment is manualised, which is important for subsequent rollout. We have outlined the 12 session content in detail[20] and listed handouts to be given to patients, but the structure allows treatment to be formulation-based so that particular issues raised in therapy that might be maintaining seizure occurrence (e.g. trauma-related issues) can be addressed. Written handouts supplement the content of face-to face therapy sessions. These will be reviewed for ease of reading by Service Users and a Patient Information Officer before the trial starts.

Standardised Medical Care

Following the initial neurology assessment (30 mins) and the psychiatric assessment session (up to 90 mins), despite some local variation we then anticipate up to 2 neurology SMC sessions (to allow a maximum of 30 mins per appointment) and 3-4 psychiatry SMC sessions (assuming up to 30 minutes per appointment) after randomisation but there will be no mandatory number.

8.5 *Intervention records*

With participants' consent, all CBT sessions will be audio-recorded using high quality digital voice recorders. Recordings will be stored on password-protected computers. These recordings will be used during the course of the study for supervision of therapists. They will also be used to assess the fidelity of the intervention.

Therapists will keep records of the therapy sessions in accordance with the guidelines of the clinical service in which they work and in accordance with professional guidelines.

Standardised medical care sessions will be recorded in medical records in accordance with local Trust guidelines and clinical practice.

8.6 *Subject Compliance.*

Research workers will contact participants every two weeks to obtain DS occurrence records and will record non-availability of this data.

The SMC doctor will record how many of the offered clinic outpatient sessions were attended, and how many were not attended during the initial 3-month period and then during the 12 months post-randomisation. This will be done by reviewing the medical notes. Data will be provided to the research worker to enter.

If the participant has been receiving CBT, the therapist will record how many sessions/part sessions out of 13 (12 + 1 booster session); whether they were face-to-face or telephone consultations and the duration of each session attended. At the end of therapy, the therapist will also score how well the participant adhered to the general therapy approach, as well as rate on a session-by-session basis how well the participant adhered to homework tasks. If DS cease early in treatment we will encourage continued attendance by patients at sessions to address other significant aspects of their presentation and to focus on relapse prevention. A protocol will be written to handle missed appointments, illness, holidays etc, to ensure that treatment is completed in the allocated time (i.e. within 4-5 months).

If participants do not attend a CBT session, the CBT therapist will contact the participant by telephone in the first instance to ascertain the problem of attendance, and will discuss the appropriate solution with the participant. Choices include a telephone session or a re

arranged face-to-face session, so long as the latter is wherever possible within five working days. Alternatively, the session stays a DNA and is recorded as such.

8.7 *Study adherence*

CBT Therapists' compliance with the Treatment Manual:

CBT therapists (health professionals, i.e. clinical psychologists/nurse therapists or other professions allied to medicine- already trained in CBT) will be trained to deliver CBT for DS. We expect each therapist to treat on average 10 trial patients. Before treating any trial patients all therapists will attend a three-day workshop, (overseen by TC) specifically focusing on DS. The workshops will include DS-specific knowledge and skills. Specific skills will be repeatedly role-played. Role-plays will be assessed using a scale initially developed for chronic fatigue[58] but subsequently adapted to assess DS-specific CBT[20]. Each therapist will have to reach a pre-determined cut-off on each item of the scale before being able to treat trial patients. Therapists will receive a combination of individual face-to-face, Skype/phone and group supervision. All therapy sessions will be audio-recorded. Some recordings will be used by supervisors to provide feedback to therapists to ensure adherence to the treatment model and specific treatment approach. Any significant deviations from the manual will be noted and fed back to the therapist. By adopting such an approach our experience from other illnesses suggests we can achieve expected treatment outcomes and have very little therapist effect[59].

Standardised Medical Care:

We will provide clinicians with study-specific written materials as well as references to pre-existing information sources (including websites). As indicated above, as part of the study set-up, members of the applicant team will undertake site visits in the lead-up to the study and in months 1-2 before recruitment starts. The study and its aims will be presented to medical staff. Staff will also be provided with 90-120 minutes of face-to-face discussion and training in the content of SMC. We will develop a Treatment Guide for those providing SMC to highlight in greater detail how the points below might be dealt with for an individual patient, including “Frequently Asked Questions” that will inform the SMC doctors how to deliver SMC. They will also have available a crib sheet for clinicians, following an approach used in other DS studies to act as a prompt and guide within sessions. This approach,

involving the provision of a crib sheet, detailed leaflet and briefing sessions for medical staff, has been shown to work in such studies[53].

8.8 *Concomitant Medication*

We will record all medications taken within the Client Service Receipt Inventory.

We anticipate that as part of SMC patients may be prescribed antidepressant medication.

As one of exclusion criteria is current benzodiazepine use exceeding the equivalent of 10mg diazepam/day, only lower doses of benzodiazepines would be permitted during the Trial. Other psychotropic medication should be given as per normal clinical practice. We will convey to those delivering SMC that there is no recognised drug treatment for dissociative seizures, so medication should not be prescribed for the seizures themselves. If patients are taking medication when randomised to CBT, the possibility of tapering and withdrawing this medication following usual prescribing guidelines should be considered, but medication withdrawal is not a requirement for entering CBT. Where tapering and withdrawal of medication is undertaken, the timing of it is likely to vary between cases, as it will depend on the individual clinical picture and progress.

Withdrawal of anti-epileptic drugs:

As noted above, within SMC, neurologists will, as part of delivering the diagnosis, explain that AED withdrawal should be gradual but that AEDs are not treating DS and may make them worse. The exceptions would be for someone with a previous history of epilepsy or taking an AED for an alternative reason (e.g.Topiramate for migraine, Pregabalin /Gabapentin/Lamotrigine for chronic pain or Valproate for Bipolar disorder) Subsequent SMC by neurologists would include supervision of AED withdrawal and overall progress review. This is an approach that would also be emphasised during the psychiatric assessment and follow-up since remaining on AEDs conveys to the patient that they have epilepsy rather than a psychiatric disorder. However, it would be emphasised that AED withdrawal would be undertaken in a measured and collaborative way and that during that process side effects would be monitored. A gradual reduction in drug dose would be recommended to avoid adverse reactions (e.g. withdrawal seizures) and this would be communicated to the patient's general practitioner to ensure that AEDs are not re-prescribed. There is evidence that a measured approach to AED withdrawal, undertaken soon after diagnosis is safe and potentially beneficial in improving seizure control and reducing health service use.[60] We would document any concerns patients have about their medications at each SMC appointment. Any adverse reactions would be recorded by the research worker at the 6 and 12-month follow-ups, reported to the ethics committee and to the DMEC.

9. Research environment

Initial recruitment will be from secondary/tertiary epilepsy/neurology clinics at point of diagnosis.

Patients will be reviewed 3 months later in liaison/neuropsychiatry outpatient clinics. Eligible patients having seizures for the previous 8 weeks who give informed consent will be randomised. SMC will consist of out-patient neurology and psychiatry treatment. CBT will be delivered on an out-patient basis. We will recruit our sample from patients receiving a diagnosis of DS at secondary/tertiary epilepsy/neurology outpatient clinics in predominantly London, the South and South-East of England, Sheffield, Leeds, Cardiff and Edinburgh/South-East Scotland. These centres either possess or have access to diagnostic procedures for DS that include video EEG telemetry and/or routinely-used video EEG seizure provocation diagnostic clinics. Research workers will be based in the Institute of Psychiatry, King's College London, the University of Edinburgh and the University of Sheffield and travel to research sites.

10. Selection and Withdrawal of Subjects

The target population for this pragmatic trial is adult outpatients with DS which persist post diagnosis by neurologists. The diagnosis of DS will primarily be established by video EEG telemetry, ictal EEG or, if this is not possible, by clinical consensus of 2 collaborating neurologists. Consensus will be adopted in cases where DS are not captured during video EEG but where there is unequivocal clinical evidence (usually from a combination of history, eyewitness description, home video of seizures or seizures witnessed in medical settings) that the episodes are inconsistent with epilepsy or any other medical diagnosis. We are adopting these criteria since not all DS patients will have access or consent to telemetry, or have seizures during telemetry. Furthermore, in a small minority of cases, clinical information may be so supportive of a DS diagnosis that clinicians feel telemetry would involve an unnecessary treatment delay. We adopted similar criteria in our previous study[20] and

have long-term NHS roll-out in mind. It has been reported that approximately 15% of patients may cease having their DS in the first 3 months following diagnosis by neurologists[53]. Thus, we will test our CBT intervention (versus control condition) in those who have continued to have seizures in the 8 weeks preceding neuropsychiatric assessment that will take place 3 months after diagnosis.

Our power calculation from which we derive our sample size is described in Section 13.1 (Sample Size).

In order to achieve this randomisation sample of 298 in total (149 to each arm), we need a larger pool of patients from which to obtain this sample. From our pilot RCT we expect that having seen a psychiatrist, 30% of eligible patients may not wish to be randomised, so we require 426 people who are still having DS to be seen by psychiatrists. This number will represent 85% of those initially diagnosed with DS (15% may be seizure free at 3 months).

Allowing also for approximately 25% of all newly diagnosed eligible DS patients to decline to take part in our study from point of diagnosis, to obtain our randomisation sample we need 668 patients diagnosed with DS from which to recruit.

Recruiting sites will include, among others, King's College Hospital, Lewisham Hospital, Guy's & St Thomas', St George's Hospital, National Hospital for Neurology and Neurosurgery, East Kent Hospitals Trust, West Kent Hospitals, Barts & The London, Royal Free, Charing Cross, Addenbrooke's Hospital Cambridge, Sussex Partnership Trust, Western General Hospital Edinburgh, Royal Hallamshire Sheffield, St James University Hospital, Leeds, University of Wales Hospital. Most of these sites have twice provided data on their annual numbers of newly diagnosed DS patients, or have participated in other studies of DS[53, 61], yielding a pool of approx. 475 newly-diagnosed DS patients per annum. Our anticipated recruitment and dropout rates are shown in Figure 1.

FIGURE 1. STUDY FLOWCHART SHOWING PATIENT FLOW THROUGH STUDY SHOWING THE DIFFERENT STAGESOF ASSESSMENT AND ALSO PROVIDES INFORMATION ON THE EXPECTED RECRUITMENT AND DROPOUT RATES

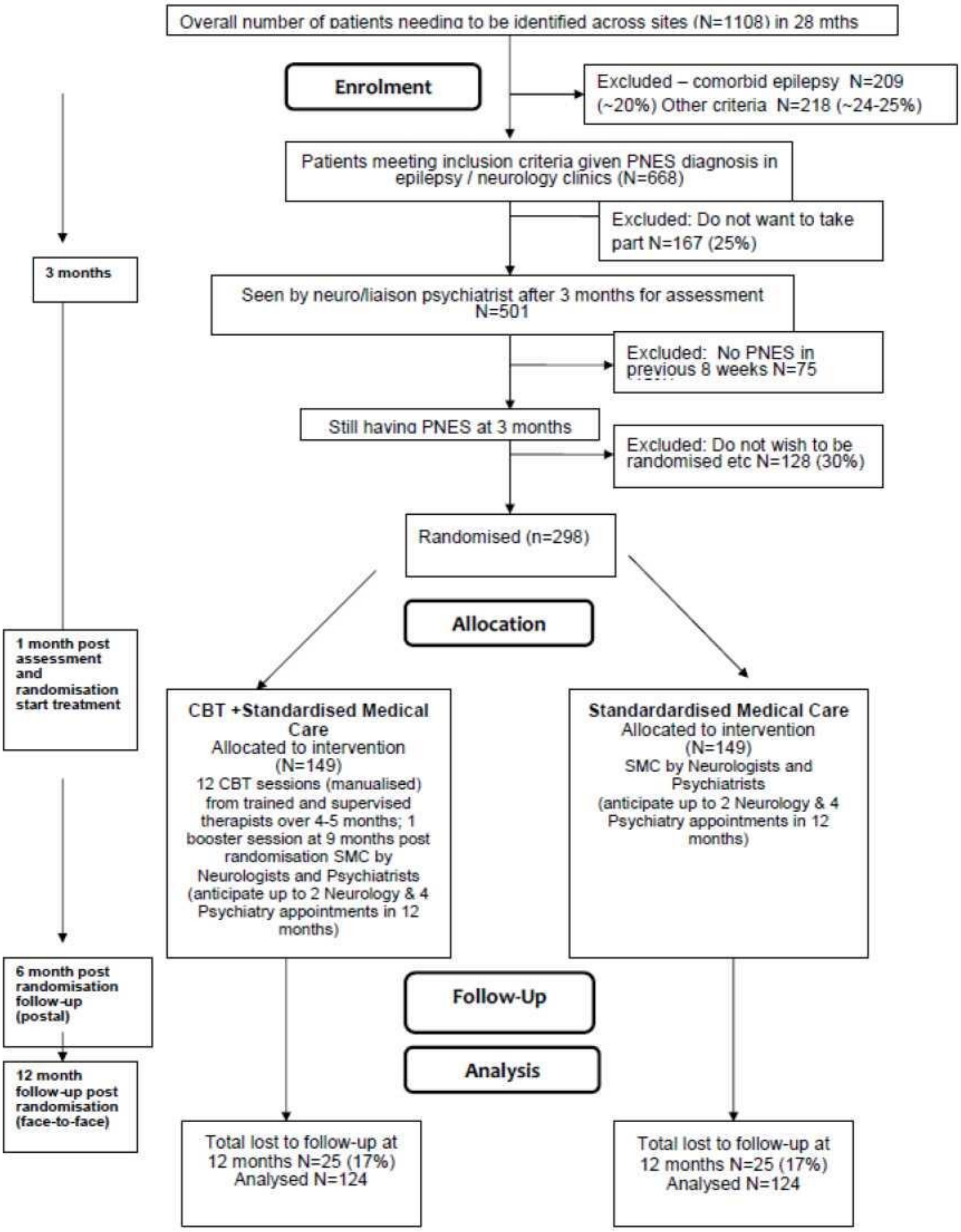

The NIHT HTA (the project funder) requires us to undertake an internal pilot during the early phase of the study with progress to be reviewed at 18 months. Essentially this will involve the same recruitment, assessment and treatment procedures as would be undertaken

throughout the study since it is recruitment and retention that is the focus of the internal pilot study rather than treatment outcomes.

We aim therefore to have all sites operating from the beginning of the recruitment phase, and to train our CBT therapists in study months 3-7. This allows for any lag by smaller or more slowly identifying sites early on, and for the opportunity to engage other sites as the study progresses and further interest in the study develops.

We will monitor our identification and recruitment rate on a month-by-month basis, and record actual numbers against targets; as indicated in our original application we will remove resources from poorly recruiting sites and increase those identifying and recruiting higher numbers.

With respect to the internal pilot, our primary assessment of feasibility for the completion of the study (on time and to target) would be at study month 18 by which time we should have identified and recruited into the study ~57% (i.e. n=~286) of our original target sample of 501 eligible PNES patients from neurology services at point of diagnosis. At this point we should also have had the opportunity to have undertaken clinical psychiatric assessments and baseline pre-randomisation assessments in ~46% (n=~232) of eligible patients. Finally we should have had the opportunity to enrol in their treatment ~39% (n=~117) of patients to be randomised to CBT or SMC.

This timing allows us to estimate all of these feasibility parameters, to evaluate our acquisition of 6-month follow-up data (of which we will have a maximum of 25% at that point) and also to have initially identified and trained CBT therapists to provide CBT across settings. This will also allow us to have previously introduced remedial measures in order to improve initially low identification / recruitment / randomisation rates.

If the numbers are slightly below expectation at this stage, then current recruitment / randomisation figures will be used to inform the Board as to whether the Trial can be completed successfully if recruitment then continues to study month 30, which is the projected point at which we need to complete recruitment. Prior to evaluating this pilot study at study month 18, we will have undertaken an internal preliminary assessment of feasibility at month 12 (10 months after data collection begins in sites). We will be judging our progress at that time on the basis of having aimed to identify a pool of ~395 newly diagnosed patients, of whom ~237 will be eligible, to have recruited ~178 and seen ~125 people in psychiatry clinics and randomised ~75. Monthly recruitment data will be made available to our DMEC. The Trial Steering Committee will also review the Trial's progress.

If only the larger sites are functioning as expected (sites seeing >10 newly diagnosed patients / year, yielding a monthly total of ~36 patients) by month 12 we should have identified a pool of ~360 patients from which we should have identified ~216 eligible patients, recruited 162 and undertaken psychiatric assessments in ~114, and randomised 68 and have started treatment (CBT +SMC or SMC alone) in ~48 patients.

If we have evidence from our existing sites that a rescue plan would from then on allow us, on an ongoing basis, to identify an average of ~41-42 newly-diagnosed patients in clinics each month, of whom ~25 would need to be eligible, ~19 could be recruited and ~11-12 /month could be randomised, then on this basis we can continue with monthly monitoring but there would be no need for alternative sites (provided that none then fail to identify/ fail to recruit satisfactorily in subsequent months). Otherwise, a rescue plan would seek to review reasons for under-recruitment etc and determine the need for additional sites within the geographical areas that could be covered by existing research staff.

However the combination of

a) an initial recruitment rate in months 3-12 of <15 eligible patients / month and

b) a subsequent failure from month 12 to increase recruitment to achieve an overall average recruitment rate of 18-19 patients / month by the end of the internal pilot at month 18 and to have achieved a resulting average randomisation rate of ~11/month, would mean that we would not have achieved our goals at month 18. It would also mean that in most likelihood, we would not be able to meet the goals for the study as a whole and we would need to plan for study closure between months 18-24.

With respect to initial identification of patients and recruitment into the study from Neurology/epilepsy clinics, the Trial Manager and Research Workers will collect site-by-site data on total numbers of DS patients screened per month, numbers consenting and reasons for exclusion according to our listed criteria. They will also collect

site-by-site data with respect to patients subsequently attending psychiatric assessment and the rates of eligibility for subsequent randomisation and reasons for exclusion.

This anonymised data will be collated centrally by the Trial Manager, entered in a specific database and plotted against targets for total monthly rates across sites. This data will be made available to the trial statistician who will report it to the DMEC on a monthly basis between meetings and will also be distributed across sites using for example online platforms such as SmartSheet.

We would suggest that monitoring data is supplied to the DMEC monthly between formal meetings and additional DMEC meetings scheduled to discuss action if recruitment falls behind target. The DMEC will be asked to advise on which sites are leading to recruitment difficulties at particular stages of the study and whether particular efforts appear to be required at such sites. They will also be asked to confirm whether certain sites appear to be more efficient than expected at recruiting participants and whether resources might be reduced in sites to concentrate on the more effective sites.

10.1 *Inclusion Criteria*

Our study comprises more than one stage and to describe our target population we need to define inclusion and exclusion criteria for each stage:

1) *Our inclusion criteria applied at the initial recruitment stage will be as follows:*

- adults (>18yrs) with DS confirmed by video EEG telemetry or, where not achievable, clinical consensus; patients who have chronic DS can be included if they have been seen by the relevant Study Neurologist who has reviewed their diagnosis and communicated this to them according to the Study protocol
- ability to complete seizure diaries and questionnaires;
- willingness to complete seizure diaries regularly and undergo psychiatric assessment 3 months after DS diagnosis;
- no documented history of intellectual disabilities;
- ability to give written informed consent.

2) Inclusion criteria evaluated at the randomisation stage will be as follows:

- adults (>18yrs) with DS initially recruited at point of diagnosis;
- willingness to continue to complete seizure diaries and questionnaires;
- provision of regular seizure frequency data following receipt of DS diagnosis;
- willingness to attend weekly/fortnightly sessions if randomised to CBT
- both clinician and patient think that randomisation is acceptable
- ability to give written informed consent.

10.2 *Exclusion Criteria*

Our exclusion criteria applied at the initial recruitment stage will be as follows:

- having a diagnosis of current epileptic seizures as well as DS. Patients with both DS and ES have been included in small studies (e.g.[32, 50]) but there is no method for verifying that patients can accurately differentiate between epileptic seizures and DS;
- inability to keep seizure records or complete questionnaires independently;
- meeting DSM-IV[7] criteria for current drug/alcohol dependence;
- insufficient command of English to later undergo CBT without an interpreter or to complete questionnaires independently. Reasons for this include the need to self-rate secondary outcomes using scales not validated for non-English speaking populations, the considerable cost and uncertainty of being able reliably to engage sufficiently competent interpreters, and the need to demonstrate the delivery of therapy in terms of quality and manual adherence.

Exclusion criteria evaluated at the randomisation stage will be as follows:

- current epileptic seizures as well as DS, for reasons given above;
- not having had any DS in the 8 weeks prior to the psychiatric assessment, 3 months post diagnosis;
- having previously undergone a CBT-based treatment for dissociative seizures at a trial participating centre
- currently having CBT for another disorder
- active psychosis;
- meeting DSM-IV[715] criteria for current drug/alcohol dependence; this may exacerbate symptoms/alter psychiatric state and health service use and affect recording of seizures;
- current benzodiazepine use exceeding the equivalent of 10mg diazepam/day;
- the patient is thought to be at imminent risk of self harm, after (neuro)psychiatric assessment and structured psychiatric assessment by the Research Worker with the

  MINI.
- known diagnosis of Factitious Disorder

10.3 *Selection of Participants*

Initial recruitment will be from secondary/tertiary epilepsy/neurology clinics at point of diagnosis.

Eligible and consenting patients will be randomised following review at 3 months in liaison/neuropsychiatry outpatient clinics.

10.4 *Randomisation Procedure / Code Break*

Randomisation will be carried out by the King's Clinical Trials Unit at the Institute of

Psychiatry using stratified randomisation. The randomisation system will be online and

completely independent of the therapy and data collection teams. Randomisation will occur after informed consent has been obtained and baseline measures have been collected. The stratification factor will be liaison/neuropsychiatry centre. The research workers and trial statistician will remain blinded.

The research workers obtaining consent will register the participant for the study by entering all the baseline data regarding the patient on the database (InformedMACRO). The system will then assign a unique identification number (PIN) to that participant which needs to be recorded on the copy of the consent form. The system will need to have the PIN, initials and DOB for the whole group entered in a sequence. Computer-generated randomisation will be conducted remotely (for more details see www.ctu.co.uk - randomisation - advanced) by the King's Clinical Trials Unit (KCTU) at the Institute of Psychiatry. We will maintain strict allocation concealment. Email confirmations will be automatically generated each time a randomisation is requested and will be sent to relevant staff with or without details of the treatment allocation included, depending on their role in the study. Specifically, the research workers will receive a confirmation of successful randomisation. The CBT therapists delivering the manualised CBT will be informed of the details of the person randomised to that intervention and will liaise with patients to arrange their attendance at appointments. Participants will be asked not to inform their research worker of their treatment allocation. Following the 6 and 12 month follow-up assessments, we will test the RWs' blinding by asking them to record when they think that allocation was revealed and record the group to which they thought patients had been allocated. The time between randomisation and the start of intervention will be kept as short as possible to minimise loss of participants prior to receiving the intervention, while allowing time to arrange attendance at CBT appointments.

The CTU database system will be set up in such a way to enable the statistician to also remain blind to treatment allocation.

10.5 *Withdrawal of Subjects*

Participants have the right to withdraw from the study at any time for any reason. The Chief Investigator also has the right to withdraw patients from the study in the event of inter-current illness, AEs, SAE's, SUSAR's, protocol

violations, cure, administrative reasons or other reasons. It is understood by all involved that an excessive rate of withdrawals can render the study uninterpretable; therefore, unnecessary withdrawal of patients will be avoided. Careful eligibility assessment is therefore essential to avoid unnecessary withdrawal of patients. Should a patient decide to withdraw from the study, all efforts will be made to report the reason for withdrawal (e.g. adverse events, inter-current illness, illness progression, inability to adhere, inability to attend regularly for treatment or assessment) as thoroughly as possible This information will be passed on to the other relevant members of the team and the Trial Manager. Should a patient withdraw from study treatment (i.e. CBT) only, efforts will be made to continue to obtain follow-up data, with the permission of the patient.

The research worker attached to the site attended by the patient wishing to withdraw will ascertain whether consent is withdrawn from further trial treatment only or from both trial treatment and follow-up and in the latter case, whether the participant has withdrawn permission to retain data collected before treatment withdrawal for use at final analysis.

Thus, randomised participants who wish to withdraw from CBT will be asked to confirm whether they are still willing to provide the following.

- study-specific data at 6 and 12 month follow-up
- seizure frequency data collected as per routine clinical practice at visits to either their neurologist/epilepsy specialist or psychiatrist

The same approach will apply to participants who withdraw from the standardised medical care arm of the study.

The Research worker attached to that centre will ensure that every effort is made to obtain the Clinical Global Impression (CGI) change score on participants who drop out of treatment as soon as this occurs, even if they are not dropping out of the trial follow-up itself.

If a participant withdraws consent for research follow-up during the trial, we will ask the clinician/therapist to notify the Trial Manager on the same day, if possible. The Trial Manager will then contact the participant to find out why the participant wishes to withdraw from research follow-up if they are happy to give a reason. They will also verify whether the participant has given permission for the research team to retain data collected before withdrawal for use at final analysis, and if not, will verify that this information should be destroyed. No data from the latter participant will be used in analysis.

With respect to a clinician/researcher's decision to withdraw a patient from the study, the reason for this must be recorded. When this occurs, the relevant clinician or nominee will need to assess the participant clinically within a week, and arrange appropriate care. Every effort will be made to obtain the primary outcome data and the CGI. Such participant's data should be included in the trial analysis. If the participant will still consent to research data collection at follow-up, this will continue as normal.

Reasons for, manner of initiation of and dates of withdrawal from the study will be recorded on a withdrawal form, which will describe the circumstances of the withdrawal.

We plan to minimise loss to follow-up in a number of ways. We plan to increase compliance with CBT attendance by encouraging patients to discuss with therapists any difficulties regarding attendance; we will assist patients' participation in the study by providing funds towards travel, and will provide appointment reminders and flexible means for patients to record seizures. We will also adopt other evidence-based procedures for recruiting and maintaining participation in the study and encouraging patients to return outcome measures, e.g. the use of incentives (vouchers at 6m and 12m follow-up), contacting people before sending out questionnaires, sending greetings cards, personalising letters, using colour printing and keeping measures short in terms of completion time[62, 63].

10.6 *Expected Duration of Trial.*

The RCT will take place during a 50-month study. It is anticipated that the initial recruitment into the study will take place from month 3 and that data collection will be completed by month 45, with database lock at month 46.

For patients identified as eligible in neurology clinics and consenting to have their seizures monitored for three months and to be referred to a psychiatrist the minimum duration in the study would be three months. Depending

on their seizure occurrence during those three months, they may or may not then be eligible for subsequent randomisation into the main trial.

For patients randomised to CBT plus SMC versus SMC alone, the maximum duration in the study would then be 12 months from randomisation.

11. Trial Procedures

11.1 *By Visit*

Stage 1

Neurologists/ epilepsy specialists will deliver the diagnosis of DS to patients attending

regular clinic appointments, give them a leaflet on Dissociative Seizures and clarify the

patients' eligibility for the study. They will discuss the diagnosis with the patient according to pre-prepared guidelines and they will have a crib sheet and series of “Frequently Asked Questions with answers to refer to. They will describe and explain that the study involves an assessment by a psychiatrist after three months (with explanations of why this may be helpful) and at the individual would be required to keep records of seizure occurrence. They will document the patient's agreement to be contacted by a member of the research team (research nurse/research worker) and give them a Participant Information Sheet and the leaflet on dissociative seizures. The neurologist/epilepsy specialist will complete a form with the patient's contact details to be passed to the research nurse/worker.

If the research nurse/worker is not present at the clinic on that day, they will telephone the patient within five working days to further explain the study and confirm eligibility criteria and the person's interest in the study. They will then arrange to meet the participant to further explain the study, obtain informed consent, collect demographic data and explain seizure monitoring forms. Participants will be contacted fortnightly by the research worker to obtain seizure frequency data by the means indicated by the participant as being preferable (phone, paper, email).

Stage 2.

At three months, the liaison/neuropsychiatrists/psychiatrist with an interest in DS will make an initial assessment of the participant during a clinic appointment. If the individual has continued to have dissociative seizures during the previous eight weeks, the psychiatrist will confirm other eligibility criteria and explain the randomised controlled trial. They will offer the patient a leaflet on dissociative seizures. Where feasible a CLRN research nurse will then support this process of explaining the study to the patient. The psychiatrist will ask for patients' agreement to be contacted by a research worker who will finalise the consenting of the patients. The psychiatrist will document the patient's agreement to be contacted by a member of the research team for this purpose.

The research worker will contact the patient within five working days to explain the study and reconfirm eligibility (i.e. by further checking inclusion and exclusion criteria) and the person's interest in the study. They will then arrange to meet the participant to further explain the study, and obtain informed consent. At this meeting, once consent has been obtained, they will undertake face-to-face administration of a structured psychiatric assessment (MINI) and administer the self-report personality measure (SAPAS-SR) as well as administer a booklet of standardised questionnaires. They will also reconfirm patients' understanding of the completion of seizure frequency data and how this should best be collected from the patient on a fortnightly basis.

In addition to recording demographics (including age, gender, age at onset of DS, duration of DS, comorbid medical diagnoses) measures completed at this stage will be:

a) monthly seizure frequency
b) Self-rated seizure severity: We will use 2 items from the Seizure Severity Scale, asking

how severe and bothersome DS were in the past month. In seizure diaries we will also ask patients to indicate how many seizures that they have had they would consider to have been severe.

c) Seizure freedom: we will record patients' self-reported previous longest period of seizure

freedom

d) Quality of life (QoL): the SF-12v2 and the EQ-5D-5L

e) Psychosocial functioning: the 5-item Work and Social Adjustment Scale (WASAS)

f) Psychiatric symptoms: GAD7, PHQ9, an extended PHQ15, and the CORE-10

g) Health service use (including hospital attendances and admissions, GP contacts), informal care, lost work time and financial benefits will be measured via the self-report Client Service Receipt Inventory.

h) We will also administer a single question measure of treatment preference asking by the patients would be preferred to be randomised to CBT plus SMC or SMC alone, a total of four questions asking them about how logical CBT and treatment by neurologists and psychiatrists seem to them and how much confidence they have that these treatments will help their condition. We will also ask them to rate how strongly they believe they have been given the correct diagnosis and how strongly they believe they have been given the correct treatment.

Seizure frequency data will then continue to be collected on a two-weekly basis by the research worker.

6-month follow-up post randomisation

This assessment will take place six months after randomisation. Participants will be sent a set of questionnaires for completion on their own and asked to return these in a prepaid envelope. We will also contact them by phone to check if they have received them and to ask if they need help with completing them. Data on potential adverse events will also be collected during this telephone contact. If questionnaires have not been returned within a two-week period of participants indicating that they have received them, the research worker will ring on two further occasions to remind participants to complete and return these, or ask the participant to complete them over the telephone

This assessment will include the following:

a) monthly seizure frequency

b) Self-rated seizure severity: We will use 2 items from the Seizure Severity Scale, asking how severe and bothersome DS were in the past month. In seizure diaries we will also ask patients to indicate how many seizures that they have had they would consider to have been severe.

c) Quality of life (QoL): the SF-12v2 and the EQ-5D-5L

d) Psychosocial functioning: the 5-item Work and Social Adjustment Scale (WASAS)

e) Psychiatric symptoms: GAD7, PHQ9 an extended PHQ15 and the CORE-10

f) Belief in Emotions, Avoidance of People, Places and Activities, belief in diagnosis and treatment

g) Health service use (including hospital attendances and admissions, GP contacts), informal care, lost work time and financial benefits, yielding estimates of ‘economic activity' will be measured via the self-report Client Service Receipt Inventory.

h) We will also ask them to rate how strongly they believe they have been given the correct diagnosis and how strongly they believe they have been given the correct treatment (2 questions).

i) Patients' self-rated global outcome (Clinical Global Impression) and satisfaction with treatment (2 questions).

If the participant has given consent for us to do this we will also seek a nominated informant's rating as to whether the person's seizures are better, same or worse than at diagnosis or whether they are seizure free.

If the questionnaire is returned to the research sites as undelivered/recipient unknown we will try to obtain the new address for the participant by writing to their last known GP provided that the participant consented to their GP being informed about their participation in the trial, or by use of their NHS or CHI number if they have consented to us having this information.

12-month follow-up post randomisation

The final follow-up will take place at 12 months post randomisation

This will be a face-to-face assessment with the research worker blind to treatment allocation and will be arranged at a location to suit the participant. The assessment will include

a) Monthly seizure frequency

b) Self-rated seizure severity: 2 items from the Seizure Severity Scale, asking how severe and bothersome DS

were in the past month. We will also ask patients to rate how many seizures that they have they would consider to have been severe.

c) Seizure freedom: we will record patients' self-reported longest period of seizure freedom between the 6 and 12-month follow-up.

e) Quality of life (QoL): the SF-12v2 and the EQ-5D-5L

f) Psychosocial functioning: the 5-item Work and Social Adjustment Scale (WASAS)

g) Psychiatric symptoms: GAD7, PHQ9. an extended PHQ15 and the CORE-10

h) Belief in Emotions, Avoidance of People Places and Activities, belief in diagnosis and treatment

i) Patients' self-rated global outcome (Clinical Global Impression) and satisfaction with treatment (2 questions).

j) Health service use (including hospital attendances and admissions, GP contacts), informal care, lost work time and financial benefits will be measured via the self-report Client Service Receipt Inventory.

k) We will also ask them to rate how strongly they believe they have been given the correct diagnosis and how strongly they believe they have been given the correct treatment (2 questions).

If the participant has given consent for us to do this we will also seek a nominated informant's rating as to whether the person's seizures are better, same or worse than at diagnosis or whether they are seizure free.

The CGI change scale will be rated by CBT therapists at end of session 12 and by SMC doctor at the 12-month follow-up, as well as by the patients themselves at 6 months and the end of the study.

12. Assessment of Safety

12.1 Specification, Timing and Recording of Safety Parameters

Adverse events will be recorded by a research worker at 6 and 12 months post

randomisation.

12.2 Procedures for Recording and Reporting Adverse Events

We will, in common with other complex intervention studies (e.g.[64]), monitor non-serious adverse events, serious adverse events, serious adverse reactions to trial treatments, serious deterioration, and active withdrawals from treatment.

We will define adverse events as 'any clinical change, disease or disorder experienced by the participant during their participation in the trial, whether or not considered related to the use of treatments being studied in the trial'. In addition, any new co-morbid medical conditions, recorded at research assessments, will be regarded as AEs if not reported at baseline. Adverse events will include any events for which the participant consulted their GP or other medical advisor or for which the participant took medication. Adverse events will also include any other events that might have affected the health status of the participant (e.g. increased work stress). Examples of non-serious adverse events might be a cold (which has not caused serious disability), an ear infection, or the experience of new pain (if not previously reported as a symptom of the participant's DS. DS themselves are not defined as adverse events.

Data on adverse events will be collected by research workers at the 6-month follow-up when they telephone participants to ensure receipt of questionnaires, and during the 12-month face-to-face follow-up.

We will require three scrutinisers (comprising neurologists or neuropsychiatrists who all specialise in working with DS patients) to review all adverse events and reactions, independently from the trial team. They will be blind to treatment group, and will be required to establish whether events reported constituted serious adverse events. The scrutinisers would then be unblinded to treatment allocation so that they can then establish whether any serious adverse events were serious adverse reactions to the main trial treatment (i.e. CBT).

We will define a serious deterioration in health as any of the following outcomes: i) a decrease by 20 points on the SF-12v2 physical function score between baseline and both the 6 and 12 month follow-up assessments, which represents a change in scores of 2 standard deviations - as for the full SF-36; ii) scores of “much worse” or “very much worse” on the participant-rated clinical global impression change in overall health scale[74] at the end

of the study; or iii) withdrawal from treatment because of a participant feeling worse (e.g. prolonged increase in DS frequency); or iv) a serious adverse reaction.

We will be guided by the following definitions.

Adverse Event (AE): Any untoward medical occurrence in a subject to whom a therapy has been administered including occurrences which are not necessarily caused by or related to that therapy.

Adverse Reaction (AR): Any untoward and unintended response in a subject to a therapy

that is related to any duration of therapy administered to that patient. An Adverse Reaction in this study can be defined as a reaction to CBT or a drug prescribed as part of SMC.

Unexpected Adverse Reaction (UAR): An adverse reaction the nature and severity of which is not consistent with the information known about the therapy in question or the underlying vulnerability to DS or other psychiatric or somatic symptoms in the view of the investigator

Serious adverse Event (SAE), Serious Adverse Reaction (SAR) or Unexpected Serious Adverse Reaction (USAR): Any adverse event, adverse reaction or unexpected adverse reaction, respectively, that

- Results in death;
- Is life-threatening (with an immediate not hypothetical risk of death at the time of the event)
- Required hospitalisation or prolongation of existing hospitalisation (but not including elective hospitalisation for pre-existing condition)
- Results in a new persistent or new significant disability or incapacity defined as: (i) severe = a significant deterioration in the participant's ability to carry out their important activities of daily living (e.g. employed person no longer able to work, caregiver no longer able to give care, ambulant participant becoming bed bound); and (ii) persistent = 4 weeks continuous duration ;
- any other important medical condition which, though not included in the above, may jeopardise the participant and may require medical or surgical intervention to prevent one of the outcomes listed,
- any new episode of deliberate self-harm

Reporting Responsibilities

All SARs and SUSARs (excepting those specified in this protocol as not requiring reporting) will be reported immediately by the Chief Investigator to the R&D office

12.2.1 Adverse events that do not require reporting

The reporting period for all events and reactions will be from randomisation to 6 month follow-up and then from 6 month follow-up to 12 month follow-up.

We will define non-serious adverse events as any health event, which was not categorised as an SAE or SAR. These will be allocated to the appropriate body system (cardiological, neurological, etc) by clinicians as defined above. Discrepancies will be resolved by consensus between the clinicians.

Examples of expected non-serious adverse events include:

- Development of new mood disorder (not leading to significant or persistent disability)
- Musculoskeletal injuries - e.g. ankle sprains etc.,
- Transient exacerbation of seizure frequency
- Development of new sleep disturbance
- Falls (e.g. due to tripping, etc.)
- Worsening of anxiety - e.g. health anxiety, exacerbated by a transient increase in symptoms

Non-serious adverse events will be reported to the DMEC via the trial statistician and will be included in the safety reporting of the completed trial.

12.3 *Stopping Rules*

The trial may be prematurely discontinued by the Sponsor or Chief Investigator on the basis of new safety information or for other reasons given by the Data Monitoring & Ethics Committee / Trial Steering Committee or ethics committee concerned.

The trial may also be prematurely discontinued due to lack of recruitment or upon advice from a Trial Steering Committee (if applicable), who will advise on whether to continue or discontinue the study and make a recommendation to the sponsor. If the study is prematurely discontinued, active participants will be informed and no further participant data will be collected. The funder (NIHR HTA) has stipulated that we include an internal pilot as part of the study (which will not use any differing procedures from those already described) and will review recruitment rates at 18 months after the commencement of the study; if it is decided that recruitment rates are insufficient then we would plan for study closure at 2 years from study start but would aim to have any CBT or SMC for patients completed although not further data would be collected on these people. This internal pilot study is described in Section 9.

13. Statistics

Bias will be avoided by the use of randomisation and blinding of outcome assessors.

All efforts will be made to avoid missing baseline data (i.e. requiring completion of baseline data before randomisation), but if this occurs, missing values will be imputed according to current recommendations (White and Thompson, 2005). Missing scale item data will be handled as per questionnaire specific recommendations.

We will aim to minimise loss to follow-up in a number of ways. We plan to increase compliance with CBT attendance by encouraging patients to discuss with therapists any difficulties regarding attendance; we will assist patients' participation in the study by providing funds towards travel, and will provide appointment reminders and flexible means for patients to record seizures. We will also adopt other evidence-based procedures for recruiting and maintaining participation in the study and encouraging patients to return outcome measures, e.g. the use of incentives (vouchers at 6m and 12m follow-up), contacting people before sending out questionnaires, sending greetings cards, personalising letters, using colour printing and keeping measures short in terms of completion time[62, 63].

13.1 Sample Size

Data from our pilot RCT study (comparing CBT and SMC on a comparable population)[1]informed sample size calculations. We based our power calculation on the effect size obtained in our pilot RCT study (comparing CBT and SMC on a comparable population)[20]. This data represents the largest study of this kind to date and importantly included a control group. Our previous pilot trial's analysis which controlled for pre-randomisation seizure frequency reported a large standardised effect size for the reduction in seizure frequency under CBT compared to SMC at the end of CBT treatment (and at a comparable time point for the SMC group) of Cohen's d=0.75 (log scale).

Importantly for the current sample size calculation, the above study also detected a more conservative and moderate effect size of Cohen's d=0.42 on the log-scale at 6 months after treatment end (median seizure frequency in the CBT group: 12 at pre-randomisation, 1.5 at 6-month follow up; median frequency in the SMC group: 8 at pre-randomisation, 5 at 6month follow-up). This follow-up time point in our previous study approximates to what will be the 12-month post randomisation follow-up in this new study, and we consider this effect clinically important. We are therefore basing our power calculation on the moderate effect size from our previous study that corresponds to the relevant time point in the proposed study.

There are a number of points that suggest that this effect size and pattern of seizure reduction are applicable to the proposed study:

1) As in the currently proposed study, patients with DS in our previous study[20] had continued to have seizures following receipt of their diagnosis from a neurologist and were seen by a psychiatrist prior to

randomisation to CBT (+ SMC) vs. SMC. Therefore, they had followed a similar care pathway.

2) Although the majority of patients in our previous study[20] were diagnosed with DS following videoEEG telemetry, as in the proposed study it was possible for patients to be diagnosed via ictal EEG or clinical consensus, again providing a comparable basis for the current study.

As in our pilot RCT[20], we will be including patients who are aged >18years of age, without documented evidence of intellectual disabilities, current drug or alcohol misuse, who do not have benzodiazepine use exceeding the equivalent of 10 mg diazepam/day and with nor current evidence of comorbid epilepsy. This again facilitates generalisability of findings from the previously studied population to the planned study.

3) Other pilot studies, albeit with smaller sample sizes than our own pilot RCT and mostly without a control condition, have reported similar patterns of reduction in seizure frequency following CBT. Thus our own open label study[55] reported a reduction in median monthly seizure frequency from 7 at baseline to 2 seizures at the end of CBT and 2 seizures at 6 month follow-up. LaFrance et al [50] reported a reduction from a median of 7 seizures/week at baseline, to 4 at one month into treatment and 0 at the end of a 12 week CBT treatment programme. Another recent small pilot RCT only published in abstract form [51], also suggests a significant reduction in seizure frequency following CBT (p=0.03) but does not provide seizure frequency data.

We are also able to consider effect sizes for other non-seizure related outcomes. In our own pilot RCT[20] the effect size for the Work and Social Adjustment Scale was d=0.9. (i.e. a large effect size). In other studies of CBT-based psychotherapy for functional symptoms, it is not uncommon to obtain moderate effect sizes. Thus, for example, a large RCT studying patients with Chronic Fatigue Syndrome that permitted a comparison between CBT and standard medical care yielded a standardised effect size of d >0.5 at 52 weeks post randomisation[64]. A RCT study of a brief guided self-help CBT approach for patients with a mixture of functional neurological symptoms (10% of whom had DS) yielded an effect size of d=0.48 at 3 months[65]. Thus, we are using an effect size comparable to that found in other CBT-based interventions with patients with functional/medically unexplained symptoms.

To detect an effect of d=0.42 with 90% power using a 2-sided t-test for log-frequencies at the 5% significance level, we need 121 participants/group. The sample size must be inflated to allow for therapist effects within the CBT group. Based on a typical therapist intraclass correlation coefficient of 0.02[66] and 15 therapists delivering CBT (average workload 10 patients/therapist), 149 participants are needed per arm to achieve 92.6% power (using the *cluspower* command in Stata allowing for clustering in only one trial arm). In our planned study, we will record pre-randomisation seizure frequencies and include this information as a covariate in the analysis model. This will increase the precision of our future intervention effect estimate. To account for this precision gain and the subsequent reduction in sample size requirement, we can apply a deflation factor to the estimated sample size. We calculated the size of this deflation factor as 0.83[67] and based on a correlation of r=0.42 between pre-randomisation and follow-up in frequencies (found in our pilot RCT). Finally, in the pilot RCT[20], 7/66 patients were lost at follow-up. We inflate the sample size allowing for a more conservative rate of 17% attrition at 12-month follow-up (i.e. we multiply by an inflation factor of 1.2). Our final randomisation target is 298 participants (149 per arm). With this sample size we will also be able to detect effects in key secondary outcomes; for example based on earlier unpublished data from our previous study[20], we will have >90% power to detect a difference in the proportion of people achieving >50% reduction of DS comparing CBT vs. standard care.

13.2 Randomisation

Randomisation will be at the level of the individual, using block randomisation with randomly varying block sizes, stratified by location of neuro/liaison psychiatry clinics from which DS patients are recruited.

13.3 *Analysis*

Statistical analyses:

Statistical analyses of the primary and secondary outcomes will adopt the intention to treat principle and will occur after database lock, with no interim analyses. For the primary frequency outcome, treatment effectiveness will be assessed by estimating the incidence rate ratio (IRR), comparing the CBT and SMC arms at the 12-month follow-up time point.

Generalised linear mixed modelling (GLMM) will provide this estimate, taking into account change at all post treatment time points. Seizure frequency at the post treatment time points (6- and 12 months post randomisation) will be the dependent variable, with baseline seizure frequency, liaison/neuropsychiatry clinics, economic activity status (i.e. whether or not on welfare benefits and employed)[21], and group, time and group x time interaction as explanatory variables. The interaction term allows for time-varying treatment effects. The model also contains subject-varying random intercepts to account for the correlation between repeated measures, doctor-varying intercepts to account for effects of the doctor delivering SMC and therapist-varying intercepts in the CBT arm to account for therapist effects. The models are estimated using maximum likelihood analysis and will allow for missing outcome data under the missing at random (MAR) assumption. The effect of departures from this assumption on results will be assessed using sensitivity analyses[68.]

Analyses of secondary outcomes will use a similar approach (mixed modelling for continuous outcome and a GLMM for the binary seizure status during the last three months of the study, and numbers showing >50% reduction in seizure frequency).

If there is considerable non-compliance with the CBT, we will also estimate the efficacy of the therapy in order to gain further insight into what drives the magnitude of the intention-to- treat effects (effectiveness evaluation).We will do this by constructing an estimate of the complier average causal effect using randomisation itself as an instrument to enable unbiased estimation of the effect of therapy receipt[69].

Health economics:

A cost-effectiveness analysis will be undertaken from a (i) health and social care and (ii) societal perspective (including lost productivity and informal care). The number and duration of CBT sessions will be centrally recorded and other service utilisation will be recorded with the Client Service Receipt Inventory[29] questionnaire at baseline, and at 6- and 12-month follow-ups. This will include primary and secondary care contacts, social care use, care from family members and medication. We will also record lost work time. As things stand, our primary analysis will be from the healthcare/social service perspective given that that is in line with NICE recommendations, with a societal perspective adopted in secondary analyses. However, it is likely that the emergence of value-based pricing will indicate a greater need to focus on societal costs. If this does occur, then we will give more weight to the latter. The unit costs of CBT sessions will be based on salaries, overheads, training and supervision. Unit costs for other services will be obtained from national sources[70, 71]. Costs of lost work and informal care will be based on average wage rates but with alternative values used in sensitivity analyses. Costs will be combined with the primary outcome measure (change in DS frequency) and also QALYs generated from the EQ-5D[39] using area-under- the-curve methods. In sensitivity analyses we will use the SF-6D, generated from the SF-12v2[38], to derive QALYs. via an algorithm developed by economists at the University of Sheffield[72]. The SF-12v2[38] contains a wider range of items than the EQ-5D and thus could reflect the impact of DS more appropriately. (A comparison of health-related quality of life has demonstrated that QALYs derived from the SF-6D performed well in discriminating between people with epilepsy with and without seizures over a two-year follow-up period[73] although there is no relevant follow-up data for DS patients.)If the intervention is less expensive and more effective than SMC then it will be ‘dominant'. If it is more expensive and more effective, incremental cost-effectiveness ratios will be constructed to show the extra cost incurred to achieve a one-unit reduction in DS frequency or one extra QALY.

Uncertainty around cost-effectiveness estimates will be explored using cost-effectiveness planes (derived from incremental cost-outcome pairs from 1000 bootstrapped resamples) and cost-effectiveness acceptability curves (CEACs derived using the net benefit approach). We have previously used 1000 resamples in the construction of CEACs. However, we are not aware of recommendations regarding this. It has though been suggested that 1000 resamples is sufficient for producing bootstrapped percentile and bias-corrected confidence intervals[74]. Given that there is virtually no cost involved in increasing the number of resamples to say 10,000 we may do this to reduce uncertainty. The CEACs will be generated using bootstrapped regression models with the group variable as the key independent variable. We will adjust for the baseline costs and utility scores. In secondary analyses, we will identify predictors of cost-effectiveness using these models and demographic and clinical baseline variables as independent variables.

In addition to the use of the SF-6D, sensitivity analyses will also be conducted around the costs of the intervention, informal care and lost employment. There has been limited previous research in this area and this trial will provide evidence on the impact of CBT over a one-year follow-up. It is the research team's view that at present there is insufficient data to populate a decision model to extrapolate the findings beyond the period of the trial but this exercise should be considered in future projects.

Qualitative analysis:

We will undertake a qualitative study to investigate the illness attributions, treatment preferences and experiences of trial participants. Interviews with all participants will be digitally recorded and transcribed. Thematic Framework Analysis[75] will be carried out by the RWs under the supervision of an experienced qualitative researcher; rigour will be increased by independent coding by at least two RWs followed by discussion meetings to agree a coding framework, to reduce bias in the interpretation of themes. Triangulation of the findings from the qualitative analysis with the results of the quantitative outcome measures will increase understanding of the trial process and may assist in understanding anomalies in outcomes.

Assessing treatment fidelity:

The recordings of CBT sessions will be used to assess the fidelity of the intervention at the level of the therapist. This will be done on the basis of rating scales already devised[1]. Two independent raters will listen to the audio-recordings and will rate the extent to which specific CBT skills are used and aspects of the therapeutic alliance. The scale will be piloted on four randomly selected sessions to determine initial inter-rater reliability and clarify the meaning of individual items to improve the clarity of coding rules. We will then undertake treatment fidelity ratings on the same two sessions selected from each therapist. The actual sessions selected for treatment integrity ratings will be selected on a pseudorandom basis (using computer-generated numbers) stratified by whether they occur early or late in the trial.

Therapists will be unaware of the sessions to be selected. Ratings for content will be made independently. Inter-rater reliability will be checked after every 10 ratings to prevent inter rater drift.

Treatment compliance and clinician characteristics

We will measure the proportion of the target population of DS patients who participated in the intervention and its components, as well as their initial treatment preferences. We will monitor the actual frequency of CBT sessions attended, whether missed sessions were due to therapists or patients, and whether any sessions needed to be conducted by phone (and if so the number). At the beginning of every session the therapist will also rate the extent to which patients engaged in homework and at the end of every session will rate their adherence with treatment overall. We will also record reasons for non-attendance such as illness or seizures.

We will also collect data on therapists (gender, age, professional backgrounds, years of experience delivering CBT) and on the doctors delivering SMC (gender, age, specialty, grade, years of clinical experience) to characterise those providing clinical care within the RCT.

14. Trial Steering Committee

We will follow HTA guidelines on appointments to the TSC and its functions. The trial steering committee will meet twice yearly. In addition, the trial management group will meet monthly to six-weekly in the first year and every 2-3 months thereafter.

15. Data Monitoring Committee

We will follow HTA guidelines on appointments to the DMEC and its functions. Members will be approved by the HTA.

The DMEC will meet twice in the first and second years of the project, once in the third and twice in the fourth year of the study. It will function according to standard operating procedures stipulated by the HTA. We will appoint a chair and a statistician who meet the HTA's requirements of independence. Following a stipulation by

the HTA, in addition to focusing on randomised participants, we will ask the DMEC to take on the role of monitoring initial patient recruitment numbers (i.e. numbers recruited at the sites). This anonymised data will be made available to the trial statistician who will report it to the DMEC on a monthly basis between meetings. Our CTU has standard operating procedures that guide the trial statistician's reporting to the DMEC. The DMEC will be asked to advise on which sites are giving rise to recruitment difficulties at particular stages of the study and whether particular efforts appear to be required at such sites. They will also be asked to confirm whether certain sites appear to be more efficient than expected at recruiting participants and whether resources might be reduced in some sites to concentrate on the more effective sites.

16. Direct Access to Source Data and Documents

The Investigator(s) will permit trial-related monitoring, audits and REC review by providing the Sponsor(s), and REC direct access to source data and other documents (e.g. patients' case sheets; consent forms; and questionnaires).

17. Ethics & Regulatory Approvals

The trial will be conducted in compliance with the principles of the Declaration of Helsinki

(1996), the principles of GCP and in accordance with all applicable regulatory requirements including but not limited to the Research Governance Framework and the Mental Capacity Act 2005.

This protocol and related documents will be submitted for review to London - Camberwell St Giles Research Ethics Committee (REC).

The Chief Investigator will submit a final report at conclusion of the trial to the funder, the REC and the Sponsor.

18. Quality Assurance

Monitoring of this trial will be to ensure compliance with Good Clinical Practice and scientific integrity will be managed by the study team

The study will be compliant with the research governance framework and MRC Good Clinical Practice Guidelines. We will institute a rigorous programme of quality control. The Trial Manager (TM) will be based at the Institute of Psychiatry, KCL and will be co-supervised by C. Murphy, CTU Manager, and line-managed and co-supervised by LG. The TM will be based within the CTU one day/week to ensure a close working relationship with the trial statistician and to support them in their role. The TM will prepare study specific Standard Operating Procedures (SOPs) for the trial, which will undergo review by the CTU Manager, and will have access to relevant CTU SOPs. The TM will supervise a designated RW employed on the study to undertake data management/cleaning, so that they can provide regular reports on data quality to LG and the other co-applicants. Quality assurance checks will be undertaken to ensure the integrity of randomisation, to monitor the level of missing data and the timeliness of data entry and check for illogical or inconsistent data. The TM will monitor data collection procedures, ensure that study data entry procedures are followed and undertake source data verification against the paper data collection forms. The trial statistician (Nick Magill) will be based in the CTU, supervised by SL, and will be responsible for DMEC reports, contribute to the Statistical Analysis Plan and carry out primary analyses.

We will ask the DMEC to take on this role of monitoring patients at recruitment. Our CTU has Standard operating procedures that guide the trial statistician's reporting to the DMEC.

19. Data Handling

The Chief Investigator will act as custodian for the trial data. The following guidelines will be strictly adhered to. Thus:

Patient data will be anonymised.

All anonymised data will be stored on a password-protected computer. All trial data will be stored in line with the Data Protection Act and archived in line with Sponsor requirements.

Consent forms and other paper records will be stored in swipe-card accessed offices in locked filing cabinets.

Access to patient data will be restricted to named individuals, members of the research team conducting the study.

Research staff with access to patient data:

Trial manager and Research Workers (to be appointed) CLRN/MHRN staff (to be identified at local sites)

CBT Therapists (to be confirmed)

20. Data Management

Data will be collected on paper source data worksheets. Data will then be entered onto the InferMed MACRO online data entry system, on a study specific database designed and hosted at the MH&N CTU. The system is compliant with Good Clinical Practice and FDA 21 CFR Part 11. Two databases will be created in order to maintain blinding. The first will be for the baseline and outcome measures (with data collected and entered by blinded RWs who will complete the baseline and follow-up assessments), the second for data on the intervention (with data relating to the CBT sessions entered by the CBT therapists who will be unblinded to treatment allocation). Randomisation and post- randomisation information will accessed directly by the trial statistician using CTU systems. The web-based randomisation system will maintain an accurate record of randomisations against targets and data can be exported directly from this system and reported to the DMEC as frequently as they wish. Post-randomisation data (follow-up rates) can be readily extracted from the MACRO trial database, which is linked to the randomisation system.

Central data cleaning will be undertaken by a designated RW. Major issues in staff training or data quality will be raised with the TM. The TM will perform source data checking against the data collection forms.

21. Publication Policy

We anticipate that there will be different target audiences for our dissemination activities:

a) Professionals: we will disseminate findings to professionals (e.g. neurologists, psychiatrists, GPs, nurses, psychologists, CBT Therapists) via papers in high impact peer reviewed journals (following extended CONSORT reporting guidelines[76]) and presentations at local, national and international scientific meetings (e.g. Association of British Neurologists, British Neuropsychiatry Association, international epilepsy conferences, a UK l ILAE meeting). We will also disseminate findings via the recently established UK Functional Neurological Symptoms (UKFNS) group and via the UK Epilepsy Research Network (UKERN). With training and support from Epilepsy Action, we will encourage our Trial Management Group lay members to present findings at a UK ILAE meeting. Findings will also be presented at the British Association of Behavioural and Cognitive Psychotherapies (BABCP) conference. We will make available our treatment manual and offer training workshops for other NHS clinical services, and at meetings (e.g. training workshops for therapists at the BABCP meetings) to disseminate good clinical practice.

b) Service planners and commissioners: if our study is successful, we anticipate that our findings will have relevance for the provision of CBT for patients with DS and therefore we will disseminate our findings to those who plan and commission care for people with neuropsychiatric disorders.

c) Voluntary sector: we will make our findings available to epilepsy charities (Epilepsy Society; Epilepsy Action) which already disseminate information on DS. While mainly supporting people with epilepsy, Epilepsy Action has regular publications (e.g. 'Epilepsy Professional' and 'Epilepsy Today' - members' magazine). We will offer summaries of our findings to websites for the public which already provide information on DS (e.g. www.neurosymptoms.org, the NEAD Trust) and to charities offering information on other dissociative disorders but not currently DS (e.g. http://www.mind.org.uk).

d) We will also publicise our work to the wider public through local and national media.

22. Insurance / Indemnity

Standard procedures for insurance of University and NHS employees and sites, and NHS patients will apply.

23. Financial Aspects

Funding to conduct the trial is provided by the NIHR HTA Programme reference number 12_26_01. The HTA has awarded £2,010,330.

24. Signatures

To be signed by Chief Investigator minimum and statistician if applicable.

4 December 2013

Chief Investigator

Date

PROFESSOR LAURA GOLDSTEIN

Appendices

Final approved documentation will be appended.

25. References

1. NICE. Transient loss of consciousness ('blackouts') management in adults and young people. London: National Institute for Health and Clinical Excellence; 2010.

2. Angus-Leppan H. Diagnosing epilepsy in neurology clinics: A prospective study. *Seizure-European Journal of Epilepsy* 2008; 17(5): 431-6.

3. Duncan R, Razvi S, Mulhern S. Newly presenting psychogenic nonepileptic seizures: Incidence, population characteristics, and early outcome from a prospective audit of a first seizure clinic. *Epilepsy and Behavior* 2011; 20(2): 308-11.

4. Reuber M, Fernandez G, Bauer J, Helmstaedter C, Elger CE. Diagnostic delay in psychogenic nonepileptic seizures *Neurology* 2002; 58: 493-5.

5. Reuber M, Pukrop R, Bauer J, Helmstaedter C, Tessendorf N, Elger CE. Outcome in psychogenic nonepileptic seizures: 1 to 10-year follow-up in 164 patients. *Annals of Neurology* 2003; 53(3): 305-11.

6. World Health Organisation. The ICD-10 Classification of Mental and Behavioural Disorders. Clinical Description and Diagnostic Guidelines. Geneva: World Health Organisation; 1992.

7. American Psychiatric Association. Diagnostic and Statistical Manual of Mental Disorders (4th Edition-DSM-IV). Washington DC American Psychiatric Association; 1994.

8. Mellers JDC. The approach to patients with 'non-epileptic seizures'. *Postgraduate Medical Journal* 2005; 81(958): 498-504.

9. Duncan R, Oto M, Wainman-Lefley J. Mortality in a cohort of patients with psychogenic non-epileptic seizures. *Journal of Neurology, Neurosurgery & Psychiatry* 2012; 83(7): 761-2.

10. Centre for Health Economics Group How Mental Illness Loses out in the NHS. In: The Centre for Economic Performance. London: The London School of Economics and Political Science 2012.

11. Al Marzooqi SM, Baker GA, Reilly J, Salmon P. The perceived health status of people with psychologically derived non-epileptic attack disorder and epilepsy: a comparative study. *Seizure* 2004; 13: 71-5.

12. Goldstein LH, Mellers JD. Ictal symptoms of anxiety, avoidance behaviour, and dissociation in patients with dissociative seizures. *Journal of Neurology, Neurosurgery & Psychiatry* 2006; 77(5): 616-21.

13. Martin RC, Gilliam FG, Kilgore M, Faught E, Kuzniecky R. Improved health care resource utilization following video-EEG-confirmed diagnosis of nonepileptic psychogenic seizures. *Seizure* 1998; 7(5): 385-90.

14. Razvi S, Mulhern S, Duncan R. Newly diagnosed psychogenic nonepileptic seizures: Health care demand prior to and following diagnosis at a first seizure clinic. *Epilepsy and Behavior* 2012; 23(1): 7-9.

15. McKenzie PS, Oto M, Graham CD, Duncan R. Do patients whose psychogenic non-epileptic seizures resolve, 'replace' them with other medically unexplained symptoms? Medically unexplained symptoms arising after a diagnosis of psychogenic non-epileptic seizures. *Journal of Neurology Neurosurgery and Psychiatry* 2011; 82(9): 967-9.

16. Goldstein LH, Mellers JDC. Recent developments in our understanding of the semiology and treatment of psychogenic nonepileptic seizures. *Curr Neurol Neurosci Rep* 2012; 12(4): 436-44.

17. Reuber M, Mayor R. Recent progress in the understanding and treatment of nonepileptic seizures. *Curr Opin Psychiatry* 2012; 25(3): 244-50.

18. Martlew JB, Gus A; Goodfellow, Laura; Bodde, Nynke; Alendkamp, Albert. Behavioural treatments for non-epileptic attack disorder. *Cochrane Epilepsy Group* 2009.

19. Mayor R, Smith PE, Reuber M. Management of patients with nonepileptic attack disorder in the United Kingdom: A survey of health care professionals. *Epilepsy & Behavior* 2011; 21(4): 402-6.

20. Goldstein LH, Chalder T, Chigwedere C, et al. Cognitive-behavioral therapy for psychogenic nonepileptic seizures A pilot RCT. *Neurology* 2010; 74(24): 1986-94.

21. Mayor R, Howlett S, Gruenewald R, Reuber M. Long-term outcome of brief augmented psychodynamic interpersonal therapy for psychogenic nonepileptic seizures: Seizure control and health care utilization. *Epilepsia* 2010; 51(7): 1169-76.

22. McKenzie P, Oto M, Russell A, Pelosi A, Duncan R. Early outcomes and predictors in 260 patients with psychogenic nonepileptic attacks. *Neurology* 2010; 74(1): 64-9.

23. Agrawal N, Fleminger S, Ring H, Deb S. Neuropsychiatry in the UK: National survey of existing service provision. *Psychiatric Bulletin* 2008; 32(8): 288-91.

24. Nice. The Epilepsies: The Diagnosis and Management of the Epilepsies in Adults and Children in Primary and Secondary Care. London, UK: National Institute for Clinical Excellence; 2004.

25. Network SIG. Diagnosis and management of epilepsy in adults. Guidelines 70. . Edinburgh: Healthcare Improvement Scotland; 2005.

26. Kerr MP, Mensah S, Besag F, et al. International consensus clinical practice statements for the treatment of neuropsychiatric conditions associated with epilepsy. *Epilepsia* 2011; 52(11): 2133-8.

27. LaFrance WCJ, Alper K, Babcock D, et al. Nonepileptic seizures treatment workshop summary. *Epilepsy and Behavior* 2006; 8(3): 451-61.

28. Kelley MS, Jacobs MP, Lowenstein DH, Stewards NEB. The NINDS epilepsy research benchmarks. *Epilepsia* 2009; 50(3): 579-82.

29. CSL. Medically Unexplained Symptoms (MUS) - A whole systems approach. In: NHS, editor. London: Commissioning Support for London; 2009 - 2010.

30. Craig P, Dieppe P, Macintyre S, Michie S, Nazareth I, Petticrew M. Developing and evaluating complex interventions: the new Medical Research Council guidance. *British Medical Journal* 2008; 337(7676).

31. Kuyk J, Siffels MC, Bakvis P, Swinkels WAM. Psychological treatment of patients with psychogenic non-epileptic seizures: An outcome study. *Seizure- European Journal of Epilepsy* 2008; 17(7): 595-603.

32. Rusch MD, Morris GL, Allen L, Lathrop LA. Psychological treatment of nonepileptic events. *Epilepsy & Behavior* 2001; 2(3,Part1): 277-83.

33. Guy W. ECDEU assessment manual for psychopharmacology. National Institute of Mental Health, Psychopharmacology Research Branch, Division of Extramural Research Programs; 1976.

34. Cramer JA, Baker GA, Jacoby A. Development of a new seizure severity questionnaire: initial reliability and validity testing. *Epilepsy Research* 2002; 48(3): 187-97.

35. Cramer JA. Assessing the severity of seizures and epilepsy: Which scales are valid? *Current Opinion in Neurology* 2001; 14(2): 225-9.

36. Cramer JA, French J. Quantitative assessment of seizure severity for clinical trials: A review of approaches to seizure components. *Epilepsia* 2001; 42(1): 119-29.

37. Birbeck GL, Vickrey BG. Determinants of health-related quality of life in adults with psychogenic nonepileptic seizures: Are there implications for clinical practice? *Epilepsia* 2003; 44(2): 141-2.

38. Ware J Jr KM, Keller SD. A 12-Item Short-Form Health Survey: construction of scales and preliminary

tests of reliability and validity. *Medical Care* 1996; 34(3): 22033.

39. Economics CfH. EuroQol--a new facility for the measurement of health-related quality of life. The EuroQol Group. York, UK: University of York; 1990.

40. Mundt JC, Marks IM, Shear MK, Greist JH. The Work and Social Adjustment Scale: a simple measure of impairment in functioning. *British Journal of Psychiatry180:461-4* 2002; 180: 461-4.

41. Spitzer RL, Kroenke K, Williams JBW, Lowe B. A brief measure for assessing generalized anxiety disorder: The GAD-7. *Archives of Internal Medicine* 2006; 166(1092-1097).

42. Kroenke K, Spitzer RL, Williams JBW. The PHQ-9: Validity of a brief depression severity measure. *Journal of General Internal Medicine* 2001; 16(9): 606-13.

43. Kroenke K, Spitzer RL, Williams JBW. The PHQ-15: Validity of a new measure for evaluating the severity of somatic symptoms. *Psychosomatic Medicine* 2002; 64: 258-66.

44. Sharpe M SJ, Hibberd C, Warlow C, Duncan R, Coleman R, Roberts R, Cull R, Pelosi A, Cavanagh J, Matthews K, Goldbeck R, Smyth R, Walker A, Walker J, MacMahon A, Murray G, Carson A. Neurology out-patients with symptoms unexplained by disease: illness beliefs and financial benefits predict 1-year outcome. *Psychological Medicine* 2010; 40(4): 689-98.

45. Lawton G, Mayor RJ, Howlett S, Reuber M. Psychogenic nonepileptic seizures and health-related quality of life: The relationship with psychological distress and other physical symptoms. *Epilepsy and Behavior* 2009; 14(1): 167-71.

46. Connell, J.C., Barkham, M. CORE-10 User Manual, version 1.1. CORE System Trust & CORE Information Management System Ltd; 2007.

47. The Core System Group The CORE System Trust & CORE Information Management System Ltd.; 2007.

48. Rimes KA, Chalder T. The Beliefs about Emotions Scale: validity, reliability and sensitivity to change. *Journal of Psychosomatic Research* 2010; 68(3): 285-92.

49. Beecham J, Knapp M. Costing psychiatric interventions. In: Thornicroft G, ed. Measuring Mental Health Needs. London: Gaskell; 2001.

50. LaFrance WC, Jr., Miller IW, Ryan CE, et al. Cognitive behavioral therapy for psychogenic nonepileptic seizures. *Epilepsy & Behavior14(4):591-6* 2009; 14(4): 591-6.

51. LaFrance Jr WC, FA W, AS B, Keitner G, J B, Szaflarski JP. Mulit-center treatment pilot for psychogenic nonepileptic seizures. Abstract No.1.218. American Epilepsy Society Annual Meeting wwwaesnetcom; 2012; 2012.

52. Reiter JM, Andrews DJ. Taking Control of Your Epilepsy. A Workbook for Patients and Professionals. Santa Rosa, CA: The BASICS Book Company; 1987.

53. Hall-Patch L, Brown R, House A, et al. Acceptability and effectiveness of a strategy for the communication of the diagnosis of psychogenic nonepileptic seizures. *Epilepsia* 2010; 51(1): 70-8.

54. Chalder T. Non-epileptic attacks: A cognitive behavioural approach in a single case approach with a four-year follow-up. *Clinical Psychology and Psychotherapy* 1996; 3(4): 291-7.

55. Goldstein LH, Deale AC, Mitchell-O'Malley SJ, Toone BK, Mellers JD. An evaluation of cognitive behavioral therapy as a treatment for dissociative seizures: a pilot study. *Cognitive & Behavioral Neurology* 2004; 17(1): 41-9.

56. Goldstein LH, LaFrance Jr WC, Chigwedere C, Mellers JDC, Chalder T. Cognitive Behavioral

Treatments. In: Schachter SC, LaFrance Jr WC, eds. Gates and Rowan's Non-Epileptic Seizures: Cambridge University Press; 2010.

57. Lang PJ. Fear reduction and fear behaviour: problems in treating a construct. In: Shilen JM, ed. Research in Psychotherapy (Vol III). Washington DC: American Psychological Association; 1968.

58. Godfrey E, Chalder T, Ridsdale L, Seed P, Ogden J. Investigating the 'active ingredients' of cognitive behaviour therapy and counselling for patients with chronic fatigue in primary care: Developing a new process measure to assess treatment fidelity and predict outcome. *British Journal of Clinical Psychology* 2007; 46(3): 253-72.

59. Cella M, Stahl D, Reme SE, Chalder T. Therapist effects in routine psychotherapy practice: An account from chronic fatigue syndrome. *Psychotherapy Research* 2011; 21(2): 168-78.

60. Oto M, Espie CA, Duncan R. An exploratory randomized controlled trial of immediate versus delayed withdrawal of antiepileptic drugs in patients with psychogenic nonepileptic attacks (PNEAs). *Epilepsia* 2010; 51(10): 1994-9.

61. Mayor R, Brown RJ, Cock H, et al. Short-term outcome of psychogenic non-epileptic seizures after communication of the diagnosis. *Epilepsy & Behavior* 2012;25(4): 676-81.

62. Edwards PR, Clarke M, DiGuiseppi, C, Pratap, S, Wentz, R, Kwan, I Increasing response rates to postal questionnaires: systematic review. *British Medical Journal* 2002; 324(1183).

63. Treweek S, Pitkethly M, Cook J, et al. Strategies to improve recruitment to randomised controlled trials. *Cochrane Database of Systematic Reviews* 2010; (4).

64. White PD, Goldsmith KA, Johnson AL, et al. Comparison of adaptive pacing therapy, cognitive behaviour therapy, graded exercise therapy, and specialist medical care for chronic fatigue syndrome (PACE): a randomised trial. *Lancet* 2011; 377(9768): 823-36.

65. Sharpe M, Walker J, Williams C, et al. Guided self-help for functional (psychogenic) symptoms A randomized controlled efficacy trial. *Neurology* 2011; 77(6): 564-72.

66. Baldwin SA, Murray DM, Shadish WR, et al. Intraclass correlation associated with therapists: estimates and applications in planning psychotherapy research. *Cognitive Behav Ther* 2011; 40(1): 15-33.

67. Borm GF FJ, Lemmens WA. A simple sample size formula for analysis of covariance in randomized clinical trials. *Journal of Clinical Epidemiology* 2007; 60(12): 1234-8.

68. White IR, Horton NJ, Carpenter J, Pocock SJ. Strategy for intention to treat analysis in randomised trials with missing outcome data. *BMJ* 2011; 342: d40.

69. Dunn G, Maracy M, Dowrick C, et al. Estimating psychological treatment effects from a randomised controlled trial with both non-compliance and loss to follow-up. *British Journal of Psychiatry* 2003; 183: 323-31.

70. Curtis L. Unit Costs of Health and Social Care 2011; 2011.

71. Department of Health. NHS Reference Costs. London: Department of Health; 2012.

72. Brazier JE, Roberts J. The estimation of a preference-based measure of health from the SF-12. *Medical Care* 2004; 42(9): 851-9.

73. Langfitt JT, Vickrey BG, McDermott MP, et al. Validity and responsiveness of generic preference-based HRQOL instruments in chronic epilepsy. *Quality of Life Research* 2006; 15(5): 899-914.

74. Briggs AH, Wonderling DE, Mooney CZ. Pulling cost-effectiveness analysis up by its bootstraps: a non-parametric approach to confidence interval estimation.*Health Econ* 1997; 6(4): 327-40.

75. Furber, C. Framework analysis: a method for analysing qualitative data. *African Journal of Midwifery and Women's Health* 2010; 4(2): 97-100.

76. Boutron I, Moher D, Altman DG, Schulz KF, Ravaud P. Extending the CONSORT statement to randomized trials of nonpharmacologic treatment: Explanation and elaboration. *Annals of Internal Medicine* 2008; 148(4): 295-309.

Changes to protocol and registration

The original RCT sample size target of N=298 from our power calculation[2,7] was revised to N=356 after the first year's recruitment, based on the observation of a potentially higher attrition rate of 35% at 6 months than the anticipated 17% but without changing other assumptions in the sample size calculation; the phase 1 target was changed from n=501 to n=698 to accommodate this new requirement and also because only 51% of those initially recruited in neurology/epilepsy clinics were continuing on to the trial, rather than the anticipated 60%. Both target revisions were agreed by the Trial Management Group, Data Monitoring and Ethics Committee, the Trial Steering Committee, the funder and the Research Ethics Committee.

Table 1: Number (%) of participants recruited at phase 1 (screening) consent and phase 2 (trial) consent per neurology site*

	Phase 1 (N=698)	Phase 2 (N=368)
1	113 (16.2)	77 (20.9)
2	55 (7.9)	15 (4.1)
3	52 (7.4)	31 (8.4)
4	51 (7.3)	30 (8.2)
5	45 (6.4)	25 (6.8)
6	42 (6.0)	18 (4.9)
7	32 (4.6)	14 (3.8)
8	29 (4.2)	16 (4.3)
9	27 (3.9)	11 (3.0)
10	26 (3.7)	9 (2.4)
11	25 (3.6)	18 (4.9)
12	21 (3.0)	12 (3.3)
13	17 (2.4)	9 (2.4)
14	17 (2.4)	7 (1.9)
15	17 (2.4)	6 (1.6)
16	17 (2.4)	4 (1.1)
17	16 (2.3)	7 (1.9)
18	16 (2.3)	7 (1.9)
19	15 (2.1)	8 (2.2)
20	13 (1.9)	10 (2.7)
21	13 (1.9)	8 (2.2)
22	12 (1.7)	8 (2.2)
23	7 (1.0)	3 (0.8)
24	7 (1.0)	3 (0.8)
25	6 (0.9)	6 (1.6)
26	6 (0.9)	5 (1.4)
27	1 (0.1)	1 (0.3)

*The 27 neurology/specialist epilepsy sites have been listed in order of total number of participants recruited rather than by name to help anonymise data from participants from sites with small numbers (<5);
N: sample size; SMC: standardised medical care; CBT: cognitive behavioural therapy.

In alphabetical order, the neurology/specialist epilepsy sites are as follows: Barts Health NHS Trust; Birmingham and Solihull Mental Health NHS Foundation Trust; Brighton and Sussex University Hospitals NHS Trust; Cambridge University Hospitals NHS Foundation Trust; Cardiff and Vale University Health Board; Chesterfield Royal Hospital NHS Foundation Trust; Croydon Health Services NHS Trust; Dartford and Gravesham NHS Trust; East Kent Hospitals University NHS Foundation Trust; East Sussex Healthcare NHS Trust; Guy's and St Thomas' NHS Foundation Trust; Imperial College Healthcare NHS Trust; King's College Hospital NHS Foundation Trust; Leeds Teaching Hospitals NHS Trust; Maidstone and Tunbridge Wells NHS Trust; Medway NHS Foundation Trust; Lewisham & Greenwich NHS Trust; Newcastle Upon Tyne Hospitals NHS Foundation Trust; NHS Lothian; Royal Berkshire NHS Foundation Trust; Royal Free London NHS Foundation Trust; Sheffield Teaching Hospitals NHS Foundation Trust; St George's University Hospitals NHS Foundation Trust; University College London Hospitals NHS Foundation Trust; University Hospital

Southampton NHS Foundation Trust; University Hospitals Birmingham NHS Foundation Trust; Western Sussex Hospitals NHS Foundation Trust.

Table 2: Number (%) of participants recruited at phase 2 (trial) consent per psychiatry site*, by trial arm and overall

	SMC (N=182)	CBT+SMC (N=186)	Overall (N=368)
1	39 (21.4)	38 (20.4)	77 (20.9)
2	29 (15.9)	29 (15.6)	58 (15.8)
3	23 (12.6)	25 (13.4)	48 (13.0)
4	15 (8.2)	15 (8.1)	30 (8.2)
5	12 (6.6)	12 (6.5)	24 (6.5)
6	9 (4.9)	10 (5.4)	19 (5.2)
7	9 (4.9)	9 (4.8)	18 (4.9)
8	8 (4.4)	8 (4.3)	16 (4.3)
9	8 (4.4)	7 (3.8)	15 (4.1)
10	7 (3.8)	8 (4.3)	15 (4.1)
11	6 (3.3)	6 (3.2)	12 (3.3)
12	4 (2.2)	6 (3.2)	10 (2.7)
13	4 (2.2)	5 (2.7)	9 (2.4)
14	4 (2.2)	3 (1.6)	7 (1.9)
15	3 (1.6)	3 (1.6)	6 (1.6)
16	2 (1.1)	1 (0.5)	3 (0.8)
17	NA	1 (0.5)	1 (0.3)

*The 17 psychiatry sites have been listed in order of total number of participants recruited (randomised) rather than by name to help anonymise data of participants from sites with small numbers (<5);
N: sample size; SMC: standardised medical care; CBT: cognitive behavioural therapy.

In alphabetical order, the psychiatry sites are as follows: Berkshire Healthcare NHS Foundation Trust; Birmingham and Solihull Mental Health Foundation Trust; Cambridgeshire and Peterborough NHS Foundation Trust; Cardiff and Vale University Health Board; Derbyshire Healthcare NHS Foundation Trust; East London NHS Foundation Trust; Kent and Medway NHS and Social Care Partnership Trust; Leeds and York Partnership NHS Foundation Trust; NHS Lothian; Northumberland Tyne and Wear NHS Foundation Trust; Sheffield Health and Social Care NHS Foundation Trust; South London and Maudsley NHS Foundation Trust; Southwest London and St George's Mental Health NHS Trust; Sussex Partnership NHS Foundation Trust; University College London Hospitals NHS Foundation Trust; University Hospital Southampton NHS Foundation Trust; West London NHS Trust.

Table 3: Baseline demographic and clinical characteristics recorded after phase 1 (screening) consent of all participants who were eligible for phase 2 (N=426), split by whether they were randomised to the trial or not

	n (%)	Not randomised (N=58)	Randomised (N=368)	Total (N=426)	P-value†
Age (years)	mean (SD)	35·8 (14·7)	37·2 (14·3)	37·0 (14·4)	0·47
	median (IQR) [range]	30 (24, 47) [18, 73]	35 (24, 47·5) [18, 78]	34 (24, 47) [18, 78]	
Gender	Female	48 (82·8)	266 (72·3)	314 (73·7)	0·11
	Male	10 (17·2)	102 (27·7)	112 (26·3)	
Ethnicity		n=57	n=368	n=425	0·053
	White	44 (77·2)	330 (89·7)	374 (88·0)	
	Asian	2 (3·5)	6 (1·6)	8 (1·9)	
	Black	2 (3·5)	6 (1·6)	8 (1·9)	
	Mixed	6 (10·5)	17 (4·6)	23 (5·4)	
	Other	3 (5·3)	9 (2·4)	12 (2·8)	
Relationship status	Single/separated/widowed	36 (62·1)	177 (48·1)	213 (50·0)	0·066
	Married/living with partner	22 (37·9)	191 (51·9)	213 (50·0)	
Living with others	Yes	47 (81·0)	315 (85·6)	362 (85·0)	0·43
	No	11 (19·0)	53 (14·4)	64 (15·0)	
Has dependents	Yes	22 (37·9)	108 (29·3)	130 (30·5)	0·22
	No	36 (62·1)	260 (70·7)	296 (69·5)	
Has a carer		n=56	n=367	n=423	1·0
	Yes	21 (37·5)	140 (38·1)	161 (38·1)	
	No	35 (62·5)	227 (61·9)	262 (61·9)	
Qualifications		n=55	n=366	n=421	0·82
	None	8 (14·5)	49 (13·4)	57 (13·5)	
	Secondary	15 (27·3)	94 (25·7)	109 (25·9)	
	Vocational	14 (25·5)	111 (30·3)	125 (29·7)	
	Further	7 (12·7)	57 (15·6)	64 (15·2)	
	Higher	11 (20·0)	55 (15·0)	66 (15·7)	
Currently employed or in education		n=58	n=365	n=423	0·55
	Yes	17 (29·3)	123 (33·7)	140 (33·1)	
	No	41 (70·7)	242 (66·3)	283 (66·9)	
Receiving disability benefits if working age (<65) and not working		n=40	n=233	n=273	0·71
	Yes	30 (75·0)	165 (70·8)	195 (71·4)	
	No	10 (25·0)	68 (29·2)	78 (28·6)	

Receiving disability benefits if working age (<65) and working		n=15	n=110	n=125	1·0
	Yes	2 (13·3)	18 (16·4)	20 (16·0)	
	No	13 (86·7)	92 (83·6)	105 (84·0)	
Dissociative seizures diagnosed by EEG and video	Yes	38 (65·5)	195 (53·0)	233 (54·7)	0·089
	No	20 (34·5)	173 (47·0)	193 (45·3)	
Age at first dissociative seizure		n=55	n=365	n=420	0·77
	mean (SD)	30·4 (13·7)	30·9 (14·1)	30·9 (14·0)	
	median (IQR) [range]	26 (20, 41) [9, 64]	29 (19, 42) [1, 76]	28 (19, 41) [1, 76]	
Years with dissociative seizures		n=55	n=365	n=420	0·65
	mean (SD)	5·6 (9·7)	6·2 (8·8)	6·1 (8·9)	
	median (IQR) [range]	2 (1, 6) [0, 64]	3 (1, 8) [0, 65]	3 (1, 7) [0, 65]	
Predominant dissociative seizure type		n=58	n=366	n=424	0·56
	Hypokinetic	18 (31·0)	130 (35·5)	148 (34·9)	
	Hyperkinetic	40 (69·0)	236 (64·5)	276 (65·1)	
Belief in diagnosis score (0=not at all; 10=extremely strongly)		n=57	n=366	n=423	0·071
	mean (SD)	7·3 (2·6)	8·0 (2·2)	7·9 (2·3)	
	median (IQR) [range]	8 (5, 10) [0, 10]	8 (7, 10) [0, 10]	8 (7, 10) [0, 10]	
Previous diagnosis of epilepsy (patient reported)	Yes	15 (25·9)	102 (27·7)	117 (27·5)	0·88
	No	43 (74·1)	266 (72·3)	309 (72·5)	
Previous diagnosis of epilepsy (doctor reported)	Yes	10 (17·2)	89 (24·2)	99 (23·2)	0·32
	No	48 (82·8)	279 (75·8)	327 (76·8)	
Currently prescribed any anti-epileptic drugs (patient reported)	Yes	17 (29·3)	96 (26·1)	113 (26·5)	0·63
	No	41 (70·7)	272 (73·9)	313 (73·5)	
Ever sought medical help for a mental health problem	Yes	39 (67·2)	234 (63·6)	273 (64·1)	0·66
	No	19 (32.8)	134 (36.4)	153 (35.9)	

†To test for an association between the variable shown in the left column of this table and whether eligible participants were randomised or not, Fisher's exact tests and Wilcoxon rank-sum tests were used for categorical and continuous variables, respectively;
N: sample size; SD: standard deviation; IQR: inter-quartile range; EEG: Electroencephalogram. All data are complete unless otherwise specified.

Table 4: Other baseline demographics and clinical characteristics of the trial sample recorded after phase 2 (trial) consent (unless otherwise specified[†])

	n (%)	SMC (N=182)	CBT+SMC (N=186)	Overall (N=368)
IMD score England (quintiles) (n=284)	1. Least deprived	15 (10·8)	16 (11·0)	31 (10·9)
	2	18 (12·9)	19 (13·1)	37 (13·0)
	3	22 (15·8)	32 (22·1)	54 (19·0)
	4	40 (28·8)	40 (27·6)	80 (28·2)
	5. Most deprived	44 (31·7)	38 (26·2)	82 (28·9)
IMD score Scotland (quintiles) (n=77)	1. Least deprived	6 (15·4)	4 (10·5)	10 (13·0)
	2	6 (15·4)	9 (23·7)	15 (19·5)
	3	6 (15·4)	7 (18·4)	13 (16·9)
	4	5 (12·8)	11 (28·9)	16 (20·8)
	5. Most deprived	16 (41·0)	7 (18·4)	23 (29·9)
IMD score Wales (quintiles) (n=7)	1. Least deprived	1 (25·0)	0 (0·0)	1 (14·3)
	2	0 (0·0)	0 (0·0)	0 (0·0)
	3	0 (0·0)	0 (0·0)	0 (0·0)
	4	0 (0·0)	1 (33·3)	1 (14·3)
	5. Most deprived	3 (75·0)	2 (66·7)	5 (71·4)
Relationship to participant if they have dependents*		n=57	n=64	n=121
	Partner	2 (3·5)	4 (6·3)	6 (5·0)
	Child	55 (96·5)	59 (92·2)	114 (94·2)
	Parent	0 (0·0)	3 (4·7)	3 (2·5)
	Other	3 (5·3)	3 (4·7)	6 (5·0)
Relationship to participant if they have a carer*		n=69	n=80	n=149
	Partner	33 (47·8)	45 (56·3)	78 (52·3)
	Child	10 (14·5)	7 (8·8)	17 (11·4)
	Parent	17 (24·6)	18 (22·5)	35 (23·5)
	Friend	5 (7·2)	8 (10·0)	13 (8·7)
	Paid	10 (14·5)	8 (10·0)	18 (12·1)
	Other	11 (15·9)	10 (12·5)	21 (14·1)
Previous CBT for disorder[†]	Yes	3 (1·6)	3 (1·6)	6 (1·6)
	No	179 (98·4)	183 (98·4)	362 (98·4)
If participant has a previous diagnosis of epilepsy, what is the status of this diagnosis[†] (doctor reported)		n=51	n=34	n=85
	Patient still has epilepsy (but no epileptic seizure in past year)	4 (7·8)	3 (8·8)	7 (8·2)
	Patient had epilepsy, but now only DS	4 (7·8)	5 (14·7)	9 (10·6)
	Patient previously misdiagnosed	25 (49·0)	18 (52·9)	43 (50·6)
	Not possible to determine validity of earlier diagnosis	18 (35·3)	8 (23·5)	26 (30·6)
		n=179	n=180	n=359

Expectation of Treatment Outcome (CBT) (0=negative; 8=positive)	mean (SD) [range]	5·7 (1·5) [2, 8]	5·8 (1·5) [1, 8]	5·8 (1·5) [1, 8]
Expectation of Treatment Outcome (Neurologist) (0=negative; 8=positive)		n=179	n=180	n=359
	mean (SD) [range]	5·4 (2·0) [0, 8]	5·4 (2·0) [0, 8]	5·4 (2·0) [0, 8]
Expectation of Treatment Outcome (Psychiatrist) (0=negative; 8=positive)		n=179	n=181	n=360
	mean (SD) [range]	5·8 (1·9) [1, 8]	5·9 (1·7) [1, 8]	5·9 (1·8) [1, 8]
Preferred Treatment Group		n=182	n=185	n=367
	CBT	108 (59·3)	120 (64·9)	228 (62·1)
	SMC	6 (3·3)	9 (4·9)	15 (4·1)
	Don't know	68 (37·4)	56 (30·3)	124 (33·8)

*Participants can respond to more than one option, so totals may add up to >100%; †Recorded after phase 1 (screening) consent;
N: sample size; SMC: standardised medical care; CBT: cognitive behavioural therapy; IMD: index of multiple deprivation; DS: dissociative seizures; SD: standard deviation. All data are complete unless otherwise specified.

Table 5: MINI-International Neuropsychiatric Interview (MINI) recorded after phase 2 (trial) consent

	n (%)	SMC (N=182)	CBT+SMC (N=186)	Overall (N=368)
Major depressive episode (Current)	No	116 (63·7)	114 (61·3)	230 (62·5)
	Yes	66 (36·3)	72 (38·7)	138 (37·5)
Major Depressive Episode (Past)	No	76 (41·8)	59 (31·7)	135 (36·7)
	Yes	106 (58·2)	127 (68·3)	233 (63·3)
Major Depressive Episode (Recurrent)	No	137 (75·3)	114 (61·3)	251 (68·2)
	Yes	45 (24·7)	72 (38·7)	117 (31·8)
Major Depressive Disorder (current)	No	129 (70·9)	125 (67·2)	254 (69·0)
	Yes	53 (29·1)	61 (32·8)	114 (31·0)
Major Depressive Disorder (Past)	No	95 (52·2)	80 (43·0)	175 (47·6)
	Yes	87 (47·8)	106 (57·0)	193 (52·4)
Suicidality	No	77 (42·3)	59 (31·7)	136 (37·0)
	Yes	105 (57·7)	127 (68·3)	232 (63·0)
Suicidality Risk Level		n=105	n=127	n=232
	Low (1-8)	71 (67·6)	80 (63·0)	151 (65·1)
	Moderate (9-16)	14 (13·3)	15 (11·8)	29 (12·5)
	High (≥17)	20 (19·0)	32 (25·2)	52 (22·4)
Manic Episode (Current)	No	175 (96·2)	182 (97·8)	357 (97·0)
	Yes	7 (3·8)	4 (2·2)	11 (3·0)
Manic Episode (Past)	No	172 (94·5)	167 (89·8)	339 (92·1)
	Yes	10 (5·5)	19 (10·2)	29 (7·9)
Hypomanic Episode (Current)	No	180 (98·9)	183 (98·4)	363 (98·6)
	Yes	2 (1·1)	3 (1·6)	5 (1·4)
Hypomanic Episode (Past)	No	174 (95·6)	177 (95·2)	351 (95·4)
	Yes	8 (4·4)	9 (4·8)	17 (4·6)
Bipolar I Disorder (current)	No	180 (98·9)	184 (98·9)	364 (98·9)
	Yes	2 (1·1)	2 (1·1)	4 (1·1)
Bipolar I Disorder (past)		n=182	n=185	n=367
	No	174 (95·6)	171 (92·4)	345 (94·0)
	Yes	8 (4·4)	14 (7·6)	22 (6·0)
Bipolar II Disorder (current)	No	181 (99·5)	184 (98·9)	365 (99·2)
	Yes	1 (0·5)	2 (1·1)	3 (0·8)
Bipolar II Disorder (past)	No	176 (96·7)	182 (97·8)	358 (97·3)
	Yes	6 (3·3)	4 (2·2)	10 (2·7)
Most Recent Episode		n=21	n=24	n=45
	Hypomanic	17 (81·0)	22 (91·7)	39 (86·7)
	Depressed	4 (19·0)	2 (8·3)	6 (13·3)
Bipolar Disorder NOS (current)		n=182	n=185	n=367
	No	180 (98·9)	184 (99·5)	364 (99·2)
	Yes	2 (1·1)	1 (0·5)	3 (0·8)

Bipolar Disorder NOS (past)		n=182	n=184	n=366
	No	178 (97·8)	182 (98·9)	360 (98·4)
	Yes	4 (2·2)	2 (1·1)	6 (1·6)
Panic Disorder (Lifetime)	No	127 (69·8)	135 (72·6)	262 (71·2)
	Yes	55 (30·2)	51 (27·4)	106 (28·8)
Panic Disorder (Limited Symptom Attacks Lifetime)	No	159 (87·4)	158 (84·9)	317 (86·1)
	Yes	23 (12·6)	28 (15·1)	51 (13·9)
Panic Disorder (Current)	No	155 (85·2)	156 (83·9)	311 (84·5)
	Yes	27 (14·8)	30 (16·1)	57 (15·5)
Agoraphobia (Current)	No	99 (54·4)	104 (55·9)	203 (55·2)
	Yes	83 (45·6)	82 (44·1)	165 (44·8)
Social Phobia [Social Anxiety Disorder] (Current)	No	148 (81·3)	145 (78·0)	293 (79·6)
	Yes	34 (18·7)	41 (22·0)	75 (20·4)
Subtype		n=34	n=41	n=75
	Generalized	26 (76·5)	38 (92·7)	64 (85·3)
	Non-Generalized	8 (23·5)	3 (7·3)	11 (14·7)
Obsessive Compulsive Disorder (Current)		n=181	n=186	n=367
	No	165 (91·2)	168 (90·3)	333 (90·7)
	Yes	16 (8·8)	18 (9·7)	34 (9·3)
Posttraumatic Stress Disorder (Current)	No	141 (77·5)	141 (75·8)	282 (76·6)
	Yes	41 (22·5)	45 (24·2)	86 (23·4)
Alcohol Dependence (Current)	No	180 (98·9)	184 (98·9)	364 (98·9)
	Yes	2 (1·1)	2 (1·1)	4 (1·1)
Alcohol Abuse (Current)	No	179 (98·4)	184 (98·9)	363 (98·6)
	Yes	3 (1·6)	2 (1·1)	5 (1·4)
Substance Dependence (Current)	No	182 (100·0)	184 (98·9)	366 (99·5)
	Yes	0 (0·0)	2 (1·1)	2 (0·5)
Substance Abuse (Current)	No	181 (99·5)	186 (100·0)	367 (99·7)
	Yes	1 (0·5)	0 (0·0)	1 (0·3)
Mood Disorder with Psychotic Features (Lifetime)	No	178 (97·8)	171 (91·9)	349 (94·8)
	Yes	4 (2·2)	15 (8·1)	19 (5·2)
Mood Disorder with Psychotic Features (Current)	No	178 (97·8)	179 (96·2)	357 (97·0)
	Yes	4 (2·2)	7 (3·8)	11 (3·0)
Psychotic Disorder (Current)	No	178 (97·8)	180 (96·8)	358 (97·3)
	Yes	4 (2·2)	6 (3·2)	10 (2·7)
Psychotic Disorder (Lifetime)	No	176 (96·7)	171 (91·9)	347 (94·3)
	Yes	6 (3·3)	15 (8·1)	21 (5·7)
Major Depressive Disorder with psychotic features (current)	No	178 (97·8)	180 (96·8)	358 (97·3)
	Yes	4 (2·2)	6 (3·2)	10 (2·7)
Major Depressive Disorder with psychotic features (past)	No	179 (98·4)	178 (95·7)	357 (97·0)
	Yes	3 (1·6)	8 (4·3)	11 (3·0)
Anorexia Nervosa (Current)	No	182 (100·0)	186 (100·0)	368 (100·0)
	Yes	0 (0·0)	0 (0·0)	0 (0·0)
Bulimia Nervosa (Current)	No	175 (96·2)	180 (96·8)	355 (96·5)

	Yes	7 (3·8)	6 (3·2)	13 (3·5)
Anorexia Nervosa (Binge Eating/ Purging)	No	182 (100·0)	186 (100·0)	368 (100·0)
	Yes	0 (0·0)	0 (0·0)	0 (0·0)
Generalized Anxiety Disorder (Current)	No	133 (73·1)	127 (68·3)	260 (70·7)
	Yes	49 (26·9)	59 (31·7)	108 (29·3)
Has an organic cause been ruled out?		n=155	n=165	n=320
	No	18 (11·6)	15 (9·1)	33 (10·3)
	Yes	87 (56·1)	86 (52·1)	173 (54·1)
	Uncertain	50 (32·3)	64 (38·8)	114 (35·6)
Antisocial Personality Disorder (Lifetime)	No	175 (96·2)	177 (95·2)	352 (95·7)
	Yes	7 (3·8)	9 (4·8)	16 (4·3)

N: sample size; SMC: standardised medical care; CBT: cognitive behavioural therapy; NOS: not otherwise specified. All data are complete unless otherwise specified.

Table 6: Demographics of all clinicians seeing participants in the trial

**Neurologists (N=91)***		
Age range	n (%)	n=89
	21-30	0 (0·0)
	31-40	21 (23·6)
	41-50	34 (38·2)
	51-60	29 (32·6)
	61-70	5 (5·6)
Gender	n (%)	n=89
	Female	34 (38·2)
	Male	55 (61·8)
Current main appointment	n (%)	n=89
	Consultant	78 (87·6)
	Specialist Registrar	4 (4·5)
	Other	7 (7·8)
Years of experience in neurology following GMC registration	mean (SD) [range]	16·3 (7·6) [4, 43] n=88
Subspecialist interest in epilepsy	n (%)	n=89
	Yes	62 (69·7)
	No	27 (30·3)
**Psychiatrists (N=29)**		
Age range	n (%)	n=29
	21-30	0 (0·0)
	31-40	4 (13·8)
	41-50	18 (62·1)
	51-60	7 (24·1)
	61-70	0 (0·0)
Gender	n (%)	n=29
	Female	8 (27·6)
	Male	21 (72·4)
Current main appointment	n (%)	n=29
	Consultant	29 (100·0)
	Specialist Registrar	0 (0·0)
	Other	0 (0·0)
Subspecialist accreditation	n (%)	n=29
	Liaison psychiatry	12 (41·4)
	Neuropsychiatry	8 (27·6)
	Both	9 (31·0)
Years of experience in psychiatry following GMC registration	mean (SD) [range]	17·8 (6·1) [4, 31] n=29
Previous experience working with patients with Dissociative Seizures (DS)	n (%)	n=29
	Yes	28 (96·6)
	No	1 (3·4)
Previous experience working with patients with Medically Unexplained Symptoms (MUS)	n (%)	n=29
	Yes	28 (96·6)
	No	1 (3·4)

Therapists (N=39)		
Age range	n (%)	n=39
	21-30	1 (2·6)
	31-40	13 (33·3)
	41-50	20 (51·3)
	51-60	5 (12·8)
	61-70	0 (0·0)
Gender	n (%)	n=39
	Female	31 (79·5)
	Male	8 (20·5)
Accredited with the BABCP	n (%)	n=39
	Yes	10 (25·6)
	No	29 (74·4)
Professional background	n (%)	n=39
	Nursing	8 (20·5)
	Clinical Psychology	20 (51·3)
	Counselling Psychology	2 (5·1)
	Occupational Therapy	3 (7·7)
	High Intensity Therapy	1 (2·6)
	Other	5 (12·8)
Highest level of post-core professional training in CBT	n (%)	n=37
	Certificate	2 (5·4)
	Diploma	10 (27·0)
	BSc	4 (10·8)
	MSc	5 (13·5)
	Doctorate	9 (24·3)
	Other	7 (18·9)
Length of CBT training (months)	mean (SD) [range]	20·2 (12·5) [0, 40] n=23
Hours of monthly CBT-specific clinical supervision	mean (SD) [range]	2·0 (0·9) [0, 5] n=31
Years of experience practicing as CBT therapist	mean (SD) [range]	10·8 (7·3) [0, 24] n=33
Previous experience working with patients with Dissociative Seizures (DS)	n (%)	n=36
	Yes	29 (80·6)
	No	7 (19·4)
Previous experience working with patients with Medically Unexplained Symptoms (MUS)	n (%)	n=36
	Yes	33 (91·7)
	No	3 (8·3)
Agenda for Change (National Health Service) Job banding (Band 7=least senior; Band 8d=most senior)	n (%)	n=36
	Band 7	15 (41·7)
	Band 8a	13 (36·1)
	Band 8b	5 (13·9)
	Band 8c	1 (2·8)
	Band 8d	2 (5·6)

*Demographic data was only collected for 89/91 neurologists: one was not registered on the database (in error); and one was registered as a psychiatrist (their main discipline) and acted as the neurologist for their site, given their epilepsy specialism;

N: sample size; GMC: General Medical Council; SD: standard deviation; BABCP: British Association for Behavioural and Cognitive Psychotherapies; CBT: cognitive behavioural therapy; BSc: Bachelor of Science degree; MSc: Master of Science degree. All data are complete unless otherwise specified.

Table 7: Participants' treatment experience

	n (%)	SMC (N=182)	CBT+SMC (N=186)	Overall (N=368)
Neurology SMC appointments offered	median (IQR) [range]	1 (0, 2) [0, 6] n=129	1 (0, 1) [0, 16] n=129	1 (0, 2) [0, 16] n=258
Neurology SMC appointments attended	median (IQR) [range]	1 (0, 2) [0, 6] n=129	1 (0, 1) [0, 14] n=129	1 (0, 1) [0, 14] n=258
Psychiatry SMC appointments offered	median (IQR) [range]	4 (3, 5) [0, 13]	3 (2, 5) [0, 10]	4 (2, 5) [0, 13]
Psychiatry SMC appointments attended	median (IQR) [range]	3 (2, 4) [0, 12]	3 (1, 4) [0, 8]	3 (1, 4) [0, 12]
Total SMC appointments offered	median (IQR) [range]	5 (3, 6) [0, 13]	4 (2, 6) [0, 20]	4·5 (3, 6) [0, 20]
Total SMC appointments attended	median (IQR) [range]	4 (2, 5) [0, 12]	3 (2, 5) [0, 19]	3 (2, 5) [0, 19]
Number of CBT sessions attended	median (IQR) [range]	NA[Θ]	13 (9, 13) [0, 13]*	NA
Distribution of number of CBT sessions attended	0	NA	8 (4·3)	NA
	1	NA	2 (1·1)	NA
	2	NA	8 (4·3)	NA
	3	NA	2 (1·1)	NA
	4	NA	7 (3·8)	NA
	5	NA	8 (4·3)	NA
	6	NA	4 (2·2)	NA
	7	NA	4 (2·2)	NA
	8	NA	3 (1·6)	NA
	9	NA	5 (2·7)	NA
	10	NA	5 (2·7)	NA
	11	NA	12 (6·5)	NA
	12	NA	14 (7·5)	NA
	13	NA	104 (55·9)	NA
**Clinical global change (therapist-reported) for those randomised to CBT+SMC**				
How well has the participant adhered to the treatment?	n (%)			n=151
	Not at all			3 (2·0)
	Slightly			32 (21·2)
	Moderately well			35 (23·2)
	Very well			42 (27·8)
	Completely			39 (25·8)
To what extent did the participant accept the model of therapy?	n (%)			n=151
	Not at all			5 (3·3)
	Slightly			24 (15·9)
	Moderately well			32 (21·2)
	Very well			32 (21·2)
	Completely			58 (38·4)
Therapist-rated global impression of change [after Session 12[†]] (0=very much worse; 6=very much better)	mean (SD) [range]			4·6 (1·0) [2, 6] n=152

*One participant received extra sessions that went beyond the trial scope: there was no gap between Session 12 and the booster, and an extra three sessions then took place because participant was deemed to be high risk by their therapist; [†]If the participant did not attend Session 12, this measure was collected from the therapist after the last session that was attended; [Θ]One participant who was allocated to SMC received 13 sessions of CODES CBT;
N: sample size; SMC: standardised medical care; CBT: cognitive behavioural therapy; IQR: inter-quartile range; NA: not applicable; SD: standard deviation. All data are complete unless otherwise specified.

The table shows that only 8 (4·3%) patients did not attend any CBT sessions. In addition (and not shown), only 42/1883 (2·2%) of attended CBT treatment sessions were disrupted due to in-session DS. Regarding attended CBT appointments the table shows therapists' ratings of their patients indicated that 59·6% of patients accepted the therapy model 'very well' or 'completely' while 21·2% accepted the model 'moderately well', 15·9% accepted the model 'slightly' and only 3·3% did not accept the model at all. In addition, 53·6% adhered to treatment between sessions 'very well' or 'completely'. Although not shown 52% completed their homework tasks with ratings of 80-100%.

Table 8: Standardised fidelity rating scores across all 36 rated sessions (100 indicated optimal rating)

Therapy fidelity ratings for all 36 rated sessions were transformed to scores out of 100. The table shows that 'Overall Therapist Adherence' achieved a median score of 85·7, 'Therapeutic Alliance' achieved a score of 92·9 and 'Overall Delivery of CBT' achieved a score of 78·6. The overall median rating across the four DS-specific skills was 58·9.

	**median (IQR) [range]**
Overall Therapist adherence (Was the therapy delivered as described in the CODES therapy manual?)	85·71 (51·79, 91·07) [21·43, 100·00]
Therapeutic Alliance (Overall, how would you rate the therapeutic alliance (supportive encouragement, understanding, warmth, empathy)?)	92·86 (78·57, 100·00) [42·86, 100·00]
Overall Delivery of CBT (Was the therapist delivering CBT?)	78·57 (50·00, 85·71) [21·43, 100·00]
Did the therapist help the client develop some strategies for controlling the seizures, which should be implemented at the first sign of a seizure?	50·00 (28·57, 85·71) [14·20, 100·00]
Did the therapist help the client make links between specific traumas or stressors and dissociative seizures?	78·57 (57·14, 92·86) [28·57, 100·00]
Did the therapist help the client challenge unhelpful beliefs related to dissociative seizures and other problems?	57·14 (42·86, 75·00) [14·29, 100·00]
Did the therapist help the client 'reclaim' areas of their life previously avoided?	64·29 (37·50, 83·93) [14·29, 100·00]
Average across DS Specific skills	58·93 (43·30, 75·89) [25·00, 100·00]

IQR: inter-quartile range; CBT: cognitive behavioural therapy; DS: dissociative seizures.

Table 9: Clinical Global Impression scores (self-, doctor- and therapist-rated), and treatment satisfaction scores at the relevant timepoints

	n (%)	6-months			12-months		
		SMC (N=182)	CBT+SMC (N=186)	Overall (N=368)	SMC (N=182)	CBT+SMC (N=186)	Overall (N=368)
Overall, how much do you feel your health has changed since the start of the study? [Self-reported CGI change score]		n=140	n=135	n=275	n=145	n=148	n=293
	Very much worse	8 (5·7)	1 (0·7)	9 (3·3)	6 (4·1)	2 (1·4)	8 (2·7)
	Much worse	13 (9·3)	4 (3·0)	17 (6·2)	19 (13·1)	11 (7·4)	30 (10·2)
	A little worse	15 (10·7)	12 (8·9)	27 (9·8)	16 (11·0)	3 (2·0)	19 (6·5)
	No change	34 (24·3)	18 (13·3)	52 (18·9)	23 (15·9)	16 (10·8)	39 (13·3)
	A little better	39 (27·9)	41 (30·4)	80 (29·1)	30 (20·7)	38 (25·7)	68 (23·2)
	Much better	16 (11·4)	38 (28·1)	54 (19·6)	29 (20·0)	42 (28·4)	71 (24·2)
	Very much better	15 (10·7)	21 (15·6)	36 (13·1)	22 (15·2)	36 (24·3)	58 (19·8)
Overall, how satisfied are you with the treatment you received? [Patient-reported satisfaction with treatment]		n=140	n=135	n=275	n=145	n=148	n=293
	Very dissatisfied	16 (11·4)	2 (1·5)	18 (6·5)	15 (10·3)	3 (2·0)	18 (6·1)
	Mod. dissatisfied	9 (6·4)	1 (0·7)	10 (3·6)	8 (5·5)	2 (1·4)	10 (3·4)
	Slightly dissatisfied	7 (5·0)	4 (3·0)	11 (4·0)	7 (4·8)	5 (3·4)	12 (4·1)
	Neither	25 (17·9)	10 (7·4)	35 (12·7)	13 (9·0)	6 (4·1)	19 (6·5)
	Slightly satisfied	15 (10·7)	12 (8·9)	27 (9·8)	20 (13·8)	9 (6·1)	29 (9·9)
	Mod. satisfied	39 (27·9)	31 (23·0)	70 (25·5)	26 (17·9)	30 (20·3)	56 (19·1)
	Very satisfied	29 (20·7)	75 (55·6)	104 (37·8)	56 (38·6)	93 (62·8)	149 (50·9)
Overall, how much has the participant changed since the start of the study? [SMC doctor (clinician) rated CGI change score]		NA	NA	NA	n=162	n=161	n=323
	Very much worse	NA	NA	NA	1 (0·6)	1 (0·6)	2 (0·6)
	Much worse	NA	NA	NA	7 (4·3)	2 (1·2)	9 (2·8)
	A little worse	NA	NA	NA	11 (6·8)	8 (5·0)	19 (5·9)
	No change	NA	NA	NA	44 (27·2)	24 (14·9)	68 (21·1)
	A little better	NA	NA	NA	49 (30·2)	38 (23·6)	87 (26·9)
	Much better	NA	NA	NA	34 (21·0)	60 (37·3)	94 (29·1)
	Very much better	NA	NA	NA	16 (9·9)	28 (17·4)	44 (13·6)
Overall, how much has the participant changed since the start of the study? [Therapist rated CGI change score after Session 12]		NA	NA	NA	NA	n=152	NA
	Very much worse	NA	NA	NA	NA	0 (0·0)	NA
	Much worse	NA	NA	NA	NA	0 (0·0)	NA
	A little worse	NA	NA	NA	NA	3 (2·0)	NA
	No change	NA	NA	NA	NA	18 (11·8)	NA
	A little better	NA	NA	NA	NA	49 (32·2)	NA
	Much better	NA	NA	NA	NA	45 (29·6)	NA
	Very much better	NA	NA	NA	NA	37 (24·3)	NA

N: sample size; SMC: standardised medical care; CBT: cognitive behavioural therapy; NA: not applicable.

Table 10: Estimated trial arm difference between CBT+SMC and SMC only at 12 months post randomisation using complete case analysis instead of multiple imputation

	Estimated trial arm difference (original scale)	(95% CI)	P-value
**Primary outcome**			
Monthly seizure frequency in last 4 weeks	†0·80	(0·58, 1·09)	0·16
**Secondary outcomes**			
Seizure severity	-0·20	(-0·61, 0·22)	0·36
Seizure bothersomeness	-0·65	(-1·10, -0·19)	*0·005
Longest period of seizure freedom in last 6 months (days)	†1·63	(1·20, 2·22)	*0·002
Seizure freedom in last 3 months of trial	ϴ1·72	(0·90, 3·29)	0·098
>50% reduction in monthly seizure frequency relative to baseline	ϴ1·29	(0·81, 2·06)	0·28
Physical Component Summary score (SF-12v2)	2·55	(0·52, 4·59)	*0·014
Mental Component Summary score (SF-12v2)	2·22	(-0·21, 4·66)	0·074
Health today (EQ-5D-5L VAS)	6·81	(2·15, 11·47)	*0·004
Impact of DS on functioning (WSAS)	-4·16	(-6·42, -1·90)	*<0·001
Anxiety (GAD-7)	-1·06	(-2·18, 0·07)	0·066
Depression (PHQ-9)	-1·22	(-2·45, 0·16)	0·053
Distress (CORE-10)	-1·61	(-2·89, -0·33)	*0·014
Other somatic symptoms (Modified PHQ-15)	-1·80	(-2·96, -0·63)	*0·003
Self-reported change (CGI)	0·78	(0·41, 1·15)	*<0·001
Clinician-rated change (CGI)	0·54	(0·28, 0·80)	*<0·001
Satisfaction with treatment (patient-reported)	1·04	(0·65, 1·44)	*<0·001

† Treatment effects for count outcomes are incidence rate ratios (IRRs); ϴ Treatment effects for binary outcomes are odds ratios (ORs); *statistically significant at 5% level (not accounting for multiple testing);
SMC: standardised medical care; CBT: cognitive behavioural therapy; CI: confidence interval; SF-12v2: Short Form 12-item (version 2) Health Survey; EQ-5D-5L: EuroQol five-dimension five-level; VAS: visual analogue scale; WSAS: Work and Social Adjustment Scale; GAD-7: Generalised Anxiety Disorder Assessment seven-item; PHQ-9: Patient Health Questionnaire nine-item; CORE-10: Clinical Outcomes in Routine Evaluation ten-item; PHQ-15: Patient Health Questionnaire fifteen-item; CGI: clinical global impression.

Table 11: Summary of all serious adverse events reported during the trial by body system

	Number of SAEs (number of participants)	Related to dissociative seizures	New or worsened health problem	Intensity	Duration (days) min-max	Required hospital admission (<24hr; >24hr)	CBT+SMC arm (%)	Relatedness to CODES CBT (CBT+SMC arm only)
Respiratory	2 (2)	0	2	1xMild<br>1xMod	1	1 (0; 1)	1 (50·0)	1xNone
Gastro-intestinal	2 (2)	0	2	1xMild<br>1xMod	5	2 (0; 2)	1 (50·0)	1xNone
Genito-urinary	4 (4)	0	4	1xMild<br>2xMod<br>1xSev	0-1	3 (1; 2)	3 (75·0)	3xNone
Haematological	3 (2)	0	3	1xMild<br>2xMod	3-4	3 (0; 3)	0 (0·0)	NA
Musculo-skeletal	6 (6)	1	6	3xMild<br>2xMod<br>1xSev	0-3	4 (1; 2)	3 (50·0)	1xRemote<br>2xNone
Neoplasia	2 (2)	0	2	1xMod<br>1xSev	0	1 (0; 1)	0 (0·0)	NA
Neurological	2 (2)	2	2	2xMod	2	2 (0; 1)	1 (50·0)	1xNone
Psychological	21 (18)	1	21	4xMild<br>12xMod<br>3xSev	0-327	6 (2; 4)	13 (61·9)	11xRemote<br>2xNone
Immunological	2 (2)	0	2	2xMod		1 (0; 1)	1 (50·0)	1xNone
Allergies	1 (1)	0	1	1xMod	1	1 (0; 1)	1 (100·0)	1xNone
Eyes, ear, nose, throat	2 (2)	0	2	1xMild<br>1xSev	0-1	2 (1; 1)	1 (50·0)	1xNone
Other	11 (11)	6	9	5xMild<br>5xMod<br>1xSev	0-68	9 (1; 8)	6 (54·5)	2xRemote<br>4xNone

CBT: cognitive behavioural therapy; SMC: standardised medical care; SAE: serious adverse event; min: minimum; max: maximum; 24hr: twenty-four hours; Mod: moderate; Sev: severe; NA: not applicable.

Overall total: 58 SAEs (49 participants); CBT+SMC arm: 31 SAEs (25 participants); SMC only arm: 27 SAEs (24 participants).

Table 12: Summary of all non-serious adverse events reported during the trial by body system

	Number of AEs (number of participants)	Related to dissociative seizures	New or worsened health problem	Intensity	Duration (days) min-max	Required hospital admission (<24hr; >24hr)	CBT+SMC arm (%)	Relatedness to CODES CBT (CBT+SMC arm only)
Respiratory	11 (11)	0	11	8xMild<br>3xMod	0-84	0	8 (72·7)	8xNone
Gastro-intestinal	12 (12)	1	11	6xMild<br>6xMod	0-12	1 (0; 1)	9 (75·0)	1xRemote<br>8xNone
Genito-urinary	9 (9)	0	9	7xMild<br>2xMod	0-21	3 (2; 0)	7 (77·8)	1xRemote<br>6xNone
Haematological	1 (1)	0	1	1xMild	-	0	0 (0·0)	NA
Musculo-skeletal	23 (22)	9	21	13xMild<br>10xMod	0-61	3 (2; 1)	9 (39·1)	2xRemote<br>7xNone
Neurological	9 (5)	6	9	4xMild<br>5xMod	0-19	0	2 (22·2)	2xRemote
Psychological	19 (14)	6	16	10xMild<br>9xMod	0-75	1 (0; 1)	11 (57·9)	9xRemote<br>2xNone
Immunological	3 (3)	0	3	3xMild	21	0	2 (66·7)	3xNone
Dermatological	4 (4)	2	4	2xMild<br>2xMod	0	0	3 (75·0)	2xRemote<br>1xNone
Allergies	1 (1)	0	1	1xMild		0	1 (100·0)	1xNone
Eyes, ear, nose, throat	10 (10)	1	9	9xMild<br>1xMod	0-15	0	8 (80·0)	2xRemote<br>6xNone
Other	16 (14)	5	15	13xMild<br>3xMod	0-61	0	6 (37·5)	6xNone

CBT: cognitive behavioural therapy; SMC: standardised medical care; AE: adverse event; min: minimum; max: maximum; 24hr: twenty-four hours; Mod: moderate; Sev: severe; NA: not applicable.

Overall total: 118 AEs (73 participants); CBT+SMC arm: 66 AEs (39 participants); SMC only arm: 52 AEs (34 participants).

Detailed Description of Statistical Analysis

Construction of the primary outcome

As described in the Statistical Analysis Plan,[7] there were two methods of collecting the primary outcome measure of monthly (four-weekly) seizure frequency, at each time point. Firstly, seizure diaries were used, which were recorded in the database as weekly counts. These were the gold-standard measure; and ideally the primary outcome at 12-months post randomisation should be calculated as the sum of four seizure diary entries for weeks 49-52.

If a diary week between 49-52 was missing, week 48 was used if available. Similarly, if a diary week between 48-52 was missing, and the total number of weeks complete was less than four, week 47 was used if available. Seizure data for weeks 53 or 54 was not available because the CODES database only recorded diary entries up to 52 weeks. Therefore, participants were recorded as having completed follow-up if they had provided at least one seizure diary entry between weeks 47-52. The type of variable that this results in is a count outcome with an observation (exposure) period. Depending on the number of completed diary weeks between 47-52, the observation period was 7, 14, 21 or 28 days.

Participants were also asked about seizure frequency at 12-months as part of a selection of self-report questions. Information from these self-report questions was used to estimate diary seizure frequency for participants who had not provided seizure diary entries between weeks 47-52 but had completed the self-report questions about seizures at 12 months. One of these questions (Question 3) asked: 'In the last 3 months, have you had a seizure?' If this was answered yes, the next question (Question 4) was opened: 'How many seizures have you had in the past 4 weeks?' Therefore, if a participant had completed question 3 (and 4 if needed), but not completed any relevant diary weeks (47-52), this auxiliary measure was used to approximate the gold-standard measure, and the participant was recorded as having completed follow-up. For these participants, their observation period was set at 28 days (4 weeks).

The same method was used when calculating monthly seizure frequency at 6-months post-randomisation, where the gold-standard should be the sum of four seizure diary entries for weeks 23-26.

If a diary week between 23-26 was missing, week 22 was used if available; if a week between 22-26 was missing and the number of weeks complete was less than four, week 21 was used if available. Similarly, if a diary week between 21-26 was missing and fewer than four weeks were complete, week 27 was used; and if a diary week between 21-27 was missing with fewer than four complete, week 28 was used if available. This meant that if a participant provided at least one weekly diary entry between weeks 21-28 inclusive, they were treated as having completed 6-month follow-up.

Self-report questions about seizures were also collected at 6-months and used to approximate diary seizure frequency in the same circumstances as the 12-months equivalent.

A pre-randomisation measure for monthly seizure frequency was collected in Phase 1, in between the participants' neurology and psychiatry appointments. This was used as the baseline measure in the analysis. The gold-standard was the sum of four weekly diary entries, over the four weeks prior to the participant's psychiatry assessment. This was when participants were reassessed for their eligibility into the trial, which included having had dissociative seizures in the 8 weeks prior to psychiatric assessment. This was also when they consented to take part in the trial and were randomised.

Phase 1 diary weeks that commenced after the date of randomisation or were inclusive of the date of randomisation were disregarded and could not be used for the pre-randomisation measure. The most recent diary week was defined as -1; the second most recent diary week was defined as -2, third most recent diary week was -3, et cetera. If a diary week was missing between -1 and -4, -5 was used if available; if a week was missing between -1 and -5 and the number of weeks complete was less than four, -6 was used if available, and so on. Diary weeks up to 56 days (-8 weeks) prior to randomisation could be used if available and needed; which meant participants were treated as having completed baseline seizure diary if they provided at least one weekly

diary entry between weeks -1 and -8 inclusive. Consecutive week numbers did not necessarily include all consecutive days, depending on how the participants had completed their diaries. However, this was allowed due to the self-report nature of the diary.

Participants were also asked to provide an estimate of seizure frequency at time of consent to take part in the trial (recorded on a baseline patient demographics form). Question 28 asked ‘How many seizures have you had in the past four weeks?’ Similar to the follow-up measures, this single measure was used to approximate baseline diary seizure frequency for participants who had not provided the relevant diary weeks prior to randomisation.

For reporting summary statistics of the primary outcome measure at any time point, if only 1-3 weeks of seizure diary had been provided, a pro-rata monthly seizure frequency was calculated. For example, if a participant had recorded a total of 6 seizures in weeks 49-51, and not provided any data for weeks 47, 48, or 52, their pro-rata monthly seizure frequency would be (6 / 3) * 4 = 8. This system of pro-rating monthly seizure frequency was also employed when calculating the secondary outcome of >50% reduction in monthly seizure frequency relative to baseline.

Details of the multiple imputation approach used to analyse primary and secondary outcome measures at 12 months

Prior to unblinding the Trial Statisticians, a binary variable was created to indicate whether participants completed 12-month follow-up data for the primary outcome or not (1=provided primary outcome data; 0=did not provide primary outcome data). This binary variable was sent to an independent King’s Clinical Trials Unit-affiliated statistician along with the CODES therapy database; the independent statistician then created a second binary variable to indicate treatment compliance. As per the SAP,[7] this was defined as attending at least nine sessions of CBT if allocated to the CBT+SMC arm (1=9 or more CBT sessions attended; 0=less than 9 CBT sessions attended). The independent statistician then ran a chi-squared test to assess whether treatment compliance within the intervention arm was predictive of missing 12-month primary outcome data in the intervention arm.

Table 13: Compliance with treatment vs. completion of 12-month monthly seizure frequency n (%)

	**Did not provide primary outcome data**	**Provided primary outcome data**
**Non-compliant**	21 (45·7)	25 (54·3)
**Compliant**	9 (6·4)	131 (93·6)
Total	30	156

The results are reported in Table 13 above: being non-compliant with the CODES CBT sessions was associated with loss to follow-up at 12-months post-randomisation in the intervention arm. The chi-squared test confirmed this association to be statistically significant ($p<0{\cdot}001$). Therefore, as described in the SAP, multiple imputation (MI), specifically multivariate imputation by chained equations (MICE[8]) was used to formally compare the 12 months primary and secondary outcome variables between the trial arms. This procedure multiply imputed missing values in order to produce inferences that are valid under the detected missing at random (MAR) data generating process. It operates by first imputing missing values in variables specified in the imputation step of the procedure, fitting an analysis model to each imputed data set, and then combining analysis results using Rubin’s rules. The procedure provides inferences that are valid under a missing data generating mechanism where the variables included in the imputation step are allowed to drive missingness.

To inform the choice of variables for the imputation model, univariate and stepwise logistic regression methods were used to detect which baseline variables were associated with loss to follow-up at 12-months, within sites (randomisation stratification factor). Fifty variables were included in this process, which consisted of all baseline variables (Table 1(main paper) and Table 3 in this document), with a few exceptions: (1) IMD quintile Wales, relationship of dependent, relationship of carer, and status of previous epilepsy diagnosis were not included because they were only measured in small sub-groups; (2) ethnicity was grouped into white, black or other, to merge small sub-groups and reduce the chance of perfect prediction; (3) the binary version of the SAPAS measure was not included (only the continuous score was used); and (4) given the number of possible diagnoses from the MINI interview, and many small sub-group numbers, only the binary variables 'any current MINI diagnosis' and 'any previous MINI diagnosis' were used.

The binary follow-up measure (as in Table 13 above) was used as the dependent variable, and all 50 baseline variables were initially tested using unadjusted logistic regression to find which ones were univariately associated with drop-out at the $\alpha<0{\cdot}05$ level. The following seven variables were significantly associated with providing primary outcome follow-up data:

1. Relationship status $p=0{\cdot}023$
2. Carer $p=0{\cdot}007$
3. Previously sought help for a mental health problem $p=0{\cdot}035$
4. Number of days seizure free in last 6-months $p=0{\cdot}005$
5. Modified PHQ-15 score $p=0{\cdot}002$
6. At least one current MINI diagnosis $p=0{\cdot}003$
7. SF-12v2 mental health composite score $p=0{\cdot}047$

These seven variables were then considered in a manual forward stepwise regression, with liberal inclusion threshold of 10%. Psychiatrist site was always included as a fixed covariate because the randomisation stratified on this. The method used was as follows:

Step 1 For each of the 7 variables, fit a logistic regression on binary follow-up with site as fixed explanatory variable; compare all the p-values and pick the variable with the lowest p-value (*var1*) if $p<0{\cdot}1$.

Step 2 For each of the remaining 6 variables, fit a logistic regression on binary follow-up with site and *var1* as explanatory variables; compare all the p-values and pick the variable with the lowest p-value (*var2*) if $p<0{\cdot}1$.

Repeat step 2 until cannot find any more variables with $p<0{\cdot}1$.

The baseline variables that were found to be associated with loss to follow-up within sites using stepwise regression (in this order) were:

1. Number of somatic symptoms on Modified PHQ-15 – those with fewer symptoms were more likely to provide primary outcome data (17 median symptoms vs 19·7, $p=0{\cdot}0030$)
2. Carer – those with a carer were more likely to provide primary outcome data than those without a carer (91·3% vs 80·8%, $p=0{\cdot}0021$)
3. Relationship status – those who were single were more likely to provide primary outcome data than those married/living with partner (89·6% vs 81·0%, $p=0{\cdot}0078$)
4. Number of days seizure free in last 6-months – those with longer periods of time without a seizure were more likely to provide primary outcome data (9·5 median days vs 7, $p=0{\cdot}0078$)
5. Previously sought help for a mental health problem – those who had never sought help were more likely to provide primary outcome data than those who had (90·6% vs 82·2%, $p=0{\cdot}037$)

As mentioned, MI consists of an imputation step and an analysis step. For each outcome variable we used the MICE procedure to impute missing values, and analysed each imputed dataset according to the chosen analysis model. As a rule, the analysis model for a 12-month outcome variable included the outcome variable as the dependent variable, and trial arm and baseline values of the outcome measure (if applicable) as the explanatory variables. For this purpose, 'baseline' was defined as the measure recorded after trial consent, except for the primary outcome, for which pre-randomisation values were recorded during the phase 1 observation period, within eight weeks prior to the psychiatry appointment. Differences between psychiatry sites (stratifier) were

accounted for by further including site-varying random intercepts in the model. Other random effects were considered but were not included in the end (see below). In terms of distributional assumptions: over-dispersed count variables (seizure frequency and seizure freedom) were modelled by a negative binomial distribution, binary variables by a binomial distribution and all other secondary outcome variables by a normal distribution. The corresponding imputation model included all the variables of the analysis model with psychiatry sites included as dummy variables. The imputation model also included treatment non-compliance in the CBT+SMC arm and the five baseline predictors found to predict primary outcome missingness at 12 months. Six-month values of the outcome measure (if applicable) were also included to ensure that all observed values of the outcome measure could contribute to the prediction of missing values.

Other random effects were also considered in the analysis model: SMC doctor and CBT therapist (in the CBT+SMC arm), but neither of these were included. Firstly, SMC doctors (most commonly psychiatrists) were nested within psychiatry sites, such that there were only one or two SMC doctors per site in over half of the sites. Therefore, SMC doctor effects could not be distinguished from site effects.

Secondly, therapist effects were assessed empirically and no evidence for their existence was found: based on complete cases, a likelihood ratio (LR) test was used for each outcome to compare the log-likelihoods of the model including therapist effects in the SMC+CBT arm with a model that did not allow for such effects. As shown in Supplementary Table 14 below, none of the likelihood-ratio tests were significant at the liberal 10% level; many of the tests reported p=1·0 (LR $\chi^2(1)=0$), and for some outcomes the model with therapist effects could not converge and so the LR test could not be run (p-value=N/A). In these instances, we interpreted the lack of convergence as therapist effects not helping to explain the variability in the outcome. Overall, we put the results down to the fact that most therapists were also nested within psychiatry sites (in the CBT+SMC arm only), and again there was only one or two therapists per site for the majority of sites.

Table 14 Results of likelihood-ratio tests for each outcome comparing models with and without random effects for therapists in the CBT+SMC arm

	**P-value of LR test**
Monthly seizure frequency	NA
Seizure severity	1.0
Seizure bothersomeness	1.0
Longest period seizure free in last 6m (days)	0.31
Seizure freedom in last 3m	NA
>50% reduction in monthly seizure frequency relative to baseline	0.53
Physical health (SF-12v2 PCS)	0.91
Mental health (SF-12v2 MCS)	0.67
Health today rating (EQ-5D VAS)	1.0
Impact of DS on functioning (WSAS)	1.0
Anxiety (GAD7)	0.77
Depression (PHQ9)	1.0
Other somatic symptoms (Modified PHQ-15)	NA
Distress (CORE10)	1.0
Self-reported change (CGI patient)	1.0
Clinician rated change (CGI doctor)	0.27
Satisfaction with treatment (CGI patient)	1.0

LR: likelihood-ratio; NA: not applicable

Therefore, the analysis model for the primary outcome seizure frequency at 12 months was a mixed effects negative binomial model, fitted using Stata command ‘menbreg’. It consisted of the dependent variable, monthly seizure frequency at 12-months (constructed as a count and exposure period as described above; with two explanatory variables: trial arm (CBT+SMC vs. SMC) and baseline monthly seizure frequency (log-transformed as in Supplementary Table 15 below); and random intercepts for psychiatry site to account for common site

experiences. The inbuilt Stata options ‘irr’ and ‘cmdok’ were used to report incidence-rate ratios and to allow estimation of the mixed effects negative binomial model with multiply imputed data.

For the primary outcome, both the imputation and analysis models assumed a negative binomial distribution. This type of model was appropriate because monthly seizure frequency was an over-dispersed count variable (variance greater than mean). Otherwise, a Poisson model would have been appropriate. Count outcomes are constructed using the number of events (count) over an exposure period (time): for our outcome this corresponded to number of seizures over number of days (7, 14, 21 or 28 days due to weekly diaries). Therefore, exposure was set to the number of days using Stata options in both.

The imputation model includes treatment non-compliance in the CMT+SMC arm and the five baseline predictors of drop-out, to allow them to drive missingness at 12 months. In addition, the imputation model included the variables of the analysis model: Trial arm, baseline values of the outcome measure and dummy variables for psychiatry sites. Previous measures of the primary outcome (baseline and 6-months) were also included. This ensured that observed values of the same outcome measured at preceding or succeeding time points could contribute to the prediction of missing values. For example, primary outcome data in a participant who only provided monthly seizure frequency at baseline and 6-months is imputed based on: Baseline and 6-months seizure frequency, and all other predictors of missingness in participants with complete data, as well as trial arm and psychiatry site.

Table 15 Variables included in the imputation step for the primary outcome

	Type of measure	Reason	Method used to impute in Chained equations approach
12-month seizure frequency	Count = number of seizures;<br>Exposure = number of days	Dependent variable of analysis model	Negative Binomial model
Baseline seizure frequency	Monthly seizure frequency (+1) log-transformed	Previous measure to contribute to prediction / explanatory variable of analysis model	NA (complete)
6-month seizure frequency			Predictive Mean Matching
Longest period of seizure freedom in last 6-months at baseline	Number of days (+1) and log-transformed	Baseline predictor of missingness	Predictive Mean Matching (only missing for 1 participant)
Relationship status	Binary dummy variable:<br>0 = Single/separated/widowed<br>1 = Married/living with partner	Baseline predictor of missingness	NA (complete)
Carer	Binary dummy variable:<br>0 = No<br>1 = Yes	Baseline predictor of missingness	NA (complete)
Previously sought help for mental health problem	Binary dummy variable:<br>0 = No<br>1 = Yes	Baseline predictor of missingness	NA (complete)
Modified PHQ-15 total	Number of somatic symptoms (continuous 0-30)	Baseline predictor of missingness	Linear regression model
Treatment non-compliance	Binary dummy variable:<br>0 = SMC arm or CBT+SMC arm with 9+ CBT sessions<br>1 = CBT+SMC arm with <9 CBT sessions	Post-randomisation predictor of missingness	NA (complete)
Trial arm	Binary dummy variable:<br>0 = SMC arm<br>1 = CBT+SMC arm	Explanatory variable of interest from analysis model	NA (complete)
Psychiatry site	15 X binary:<br>Dummy variable 1 = Site 2<br>Dummy variable 2 = Site 3<br>Dummy variable 3 = Site 4<br>Dummy variable 4 = Site 5<br>Dummy variable 5 = Site 6<br>Dummy variable 6 = Site 7<br>Dummy variable 7 = Site 8<br>Dummy variable 8 = Site 10<br>Dummy variable 9 = Site 11<br>Dummy variable 10 = Site 12<br>Dummy variable 11 = Site 13<br>Dummy variable 12 = Site 14<br>Dummy variable 13 = Site 15<br>Dummy variable 14 = Site 17<br>Dummy variable 15 = Sites 1 and 16<br>(Since site 9 had the largest number of participants, it was used as the reference site and was therefore left out here)	Randomisation stratification factor as fixed effects to ensure the imputation model was more general than analysis model (included as random intercepts in analysis model)	NA (complete)

NA: not applicable.

Table 15 above details all auxiliary variables included in the imputation step of the MICE procedure for the primary outcome. Unless complete (N=368), all auxiliary variables were imputed simultaneously (registered as imputed variables within Stata mi commands). For the primary outcome, incomplete auxiliary variables comprised of the 6-month measure for seizure frequency, longest period of time seizure free in last 6-months, and baseline modified PHQ-15 score. All other auxiliary variables were complete (registered as regular variables within Stata mi commands).

The auxiliary count variable longest period seizure free days was log-transformed before inclusion in the imputation step (one unit was added first to avoid logging zero). The reason for including longest period of time (days) seizure free in last 6-months on the log-scale was because it is inversely related to the primary outcome. Note that although the measure refers to a 6 months period it is self-report and there is no maximum value.

Baseline and 6-month seizure frequency were also log-transformed because the relationships between both of them and 12-month seizure frequency are multiplicative, which relates into an additive effect of log-counts on

the linear prediction scale. Predictive mean matching was used to impute both incomplete count variables because of zero-inflated distributions.

The total number of symptoms on the Modified PHQ-15 scale is a continuous outcome with a range of 0-22 and was therefore imputed using linear regression.

To avoid perfect prediction or over-parameterisation, relationship status was dichotomised into a binary measure, and the two smallest psychiatry sites were merged (Site 1 had one participant and Site 16 had three). All dummy variables were coded 0 and 1: relationship status, carer, previously sought help for a mental health problem, treatment compliance, trial arm, and 15 psychiatry sites.

Multiple imputation theory requires all variables of the analysis model to be included in the imputation model. Psychiatry sites were included as random intercepts in the analysis model, therefore, we included psychiatry site dummy variables as fixed effects in the imputation model. This was permissible because a fixed effects model is known to be a more general model than a random effects formulation.

Secondary outcomes were analysed following the same approach: using multiple imputation to allow for the detected missing at random process. For all outcomes, the imputation step included: the five identified predictors of loss to follow-up at 12-months; treatment compliance in the CBT+SMC arm, trial arm, psychiatry site dummy variables, and any previous measures (baseline or 6-months) of the same variable.

Some secondary outcomes were imputed simultaneously (i.e. imputations for both outcome variables were generated from a single larger imputation model) if they were strongly correlated and had different missing patterns. These were: seizure severity and bothersomeness; CGI change score self-report and doctor-rated.

Mixed effects regression with site-varying random intercepts was used for the secondary continuous outcomes: seizure severity; seizure bothersomeness; physical health (SF-12v2 PCS); mental health (SF-12v2 MCS); health today (EQ-5D-5L VAS); impact on functioning (WSAS); anxiety (GAD-7); depression (PHQ-9); somatic symptoms (modified PHQ-15); distress (CORE-10); CGI change self-report; CGI change doctor-rated; and satisfaction with treatment.

A mixed effects negative binomial model with site-varying random intercepts was used for the secondary count outcome: Self-reported longest period of time (days) seizure free in last 6-months.

Mixed-effects logistic regression with site-varying random intercepts was used for the secondary binary outcomes: seizure freedom in last 3-months of study; and >50% reduction in seizure frequency.

Extended discussion of SMC

In the Discussion section we raise the possibility that SMC may have been an intervention in its own right, even though it was conceived of as a control condition. We suggested that although we designated our control condition "Standardised Medical Care", it might be better conceptualised as Standardised Specialist Care.

In hindsight we are considering this for a number of reasons. As part of SMC, patients received what was conceived of as optimised medical care involving both neurology and psychiatry which included several treatment components developed for the trial. Thus, clinicians received guidance on diagnosis delivery involving a suggested script and patients received two detailed booklets about DS. Clinicians could describe (but not practise with patients) distraction techniques to avert seizures and directed them to self-help websites containing suggestions for seizure control techniques. Elsewhere,[9] we have reported participating neurologists' satisfaction with our treatment components. While this study does not provide evidence to support the effectiveness of our control treatment pathway, our findings in relation to CBT are clear. While we were not able to demonstrate an advantage of CBT+SMC over SMC-alone in terms of the primary outcome, our findings suggest that targeted CBT is beneficial as an adjunct to the care provided by neurologists and psychiatrists delivering this specialist care when considering other seizure-related and clinically-relevant secondary outcomes.

Raw data plots to illustrate observed changes over time, by trial arm.

Baseline was recorded pre-randomisation, and 6- and 12-months were recorded post randomisation. Trial endpoints were variables measured at 12 months. Six-month measures were not formally assessed in this study but are included here for information.

Figure 1 Plot over time of seizure severity by trial arm

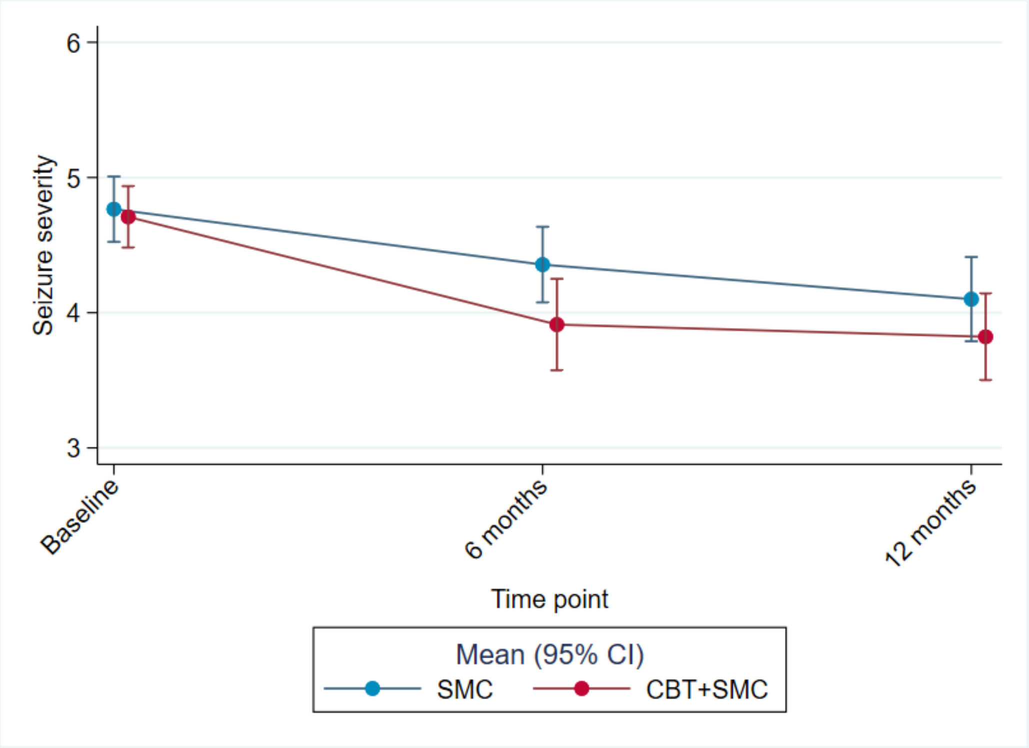

Figure 2 Plot over time of seizure bothersomeness by trial arm

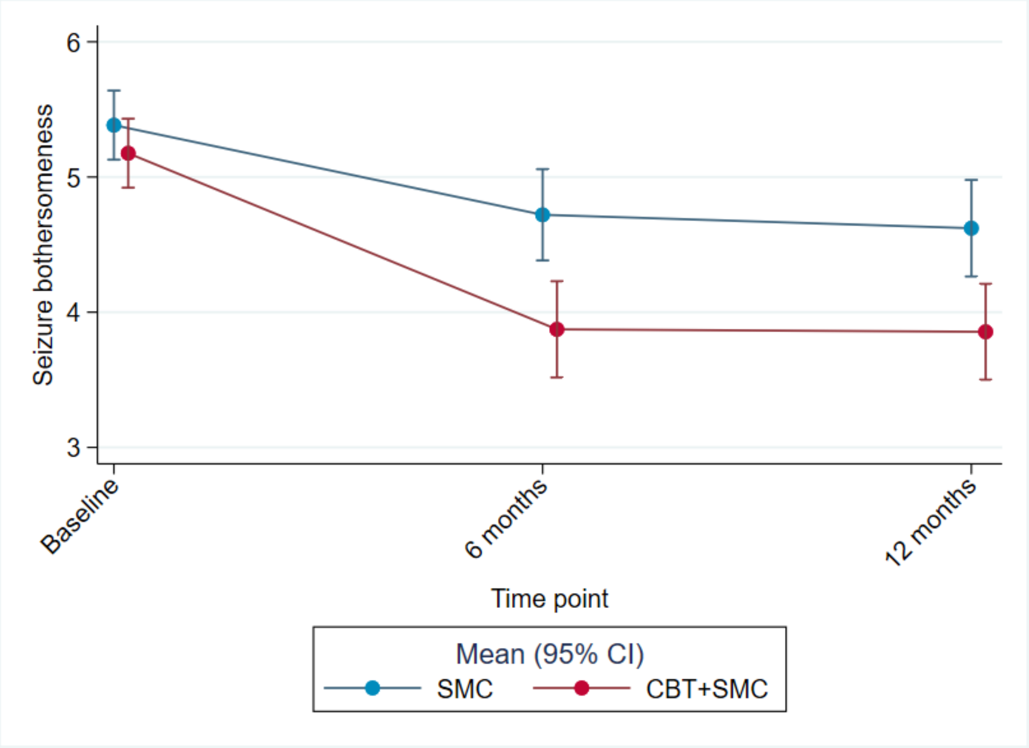

Figure 3 Plot over time of longest period of seizure freedom in last 6 months (days) by trial arm

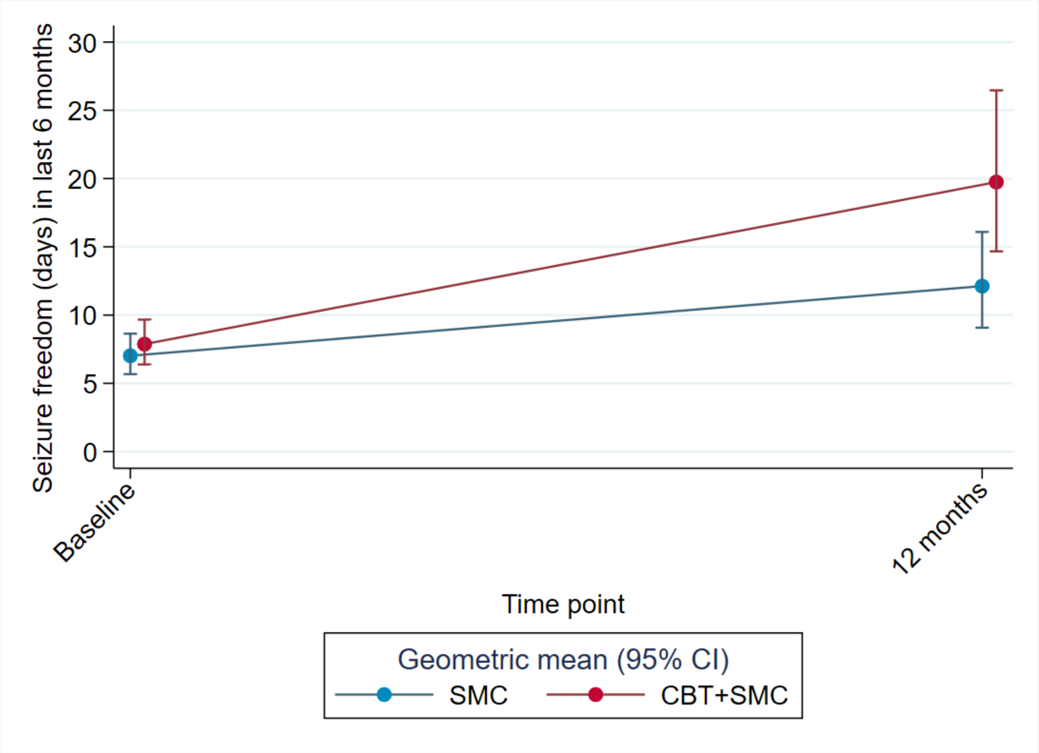

Figure 4 Seizure freedom in the last 3 months of trial (%) by trial arm

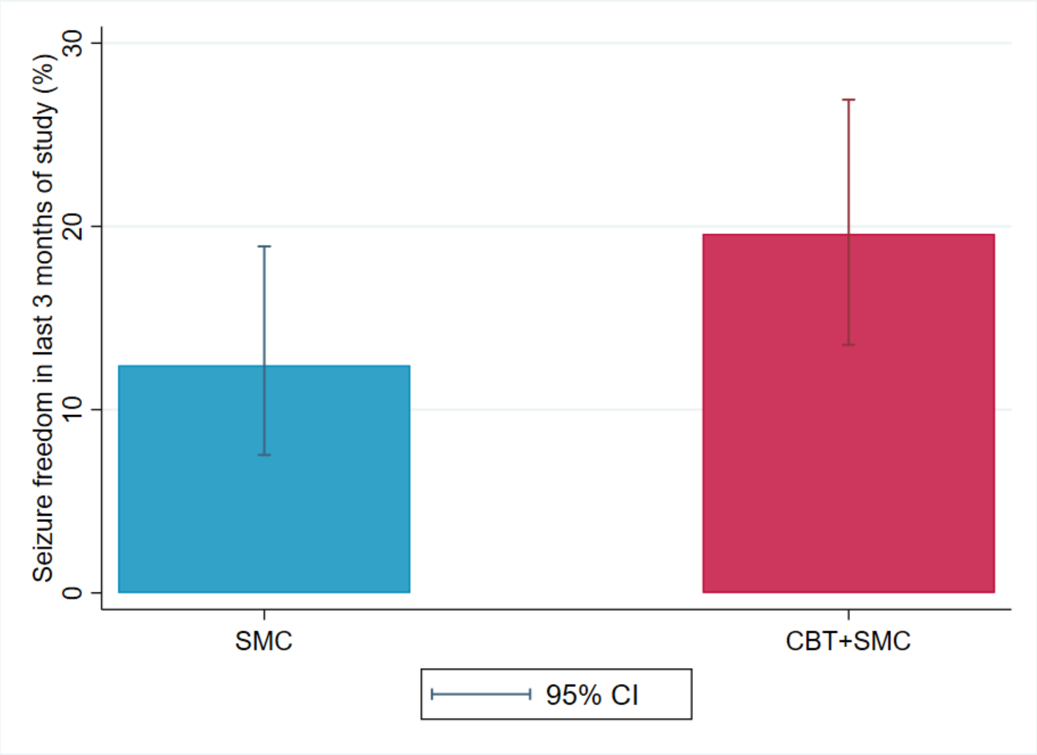

Figure 5 Greater than 50% reduction in monthly seizure frequency relative to baseline (%) by trial arm

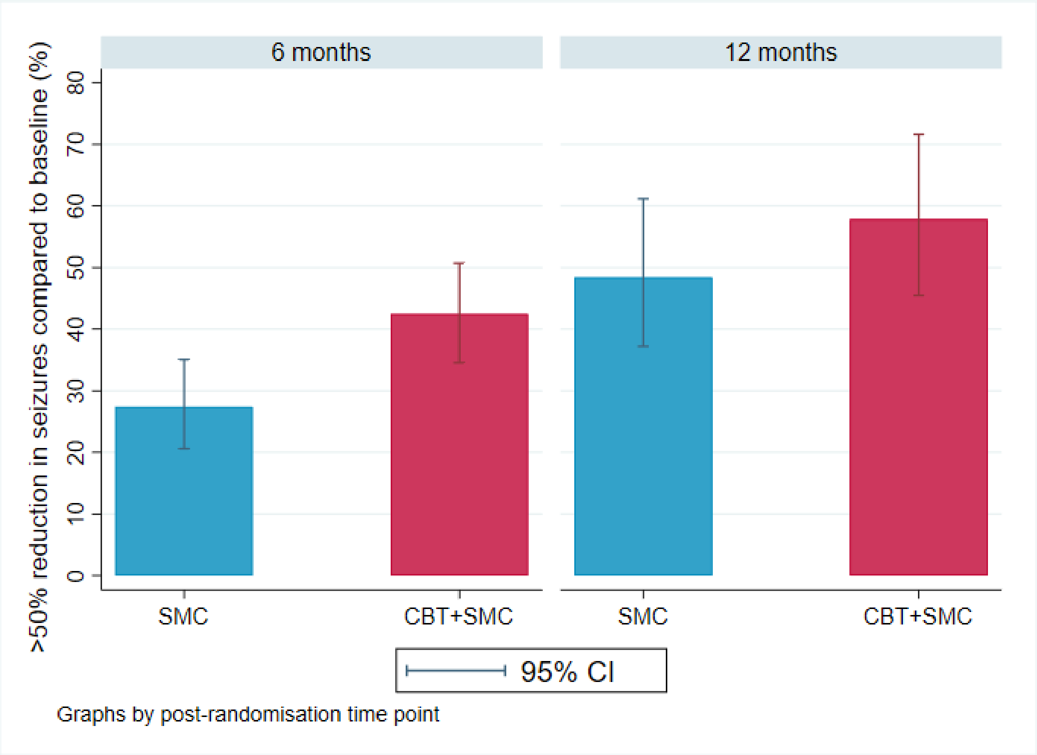

Figure 6 Plot over time of SF-12v2 PCS score (physical health) by trial arm

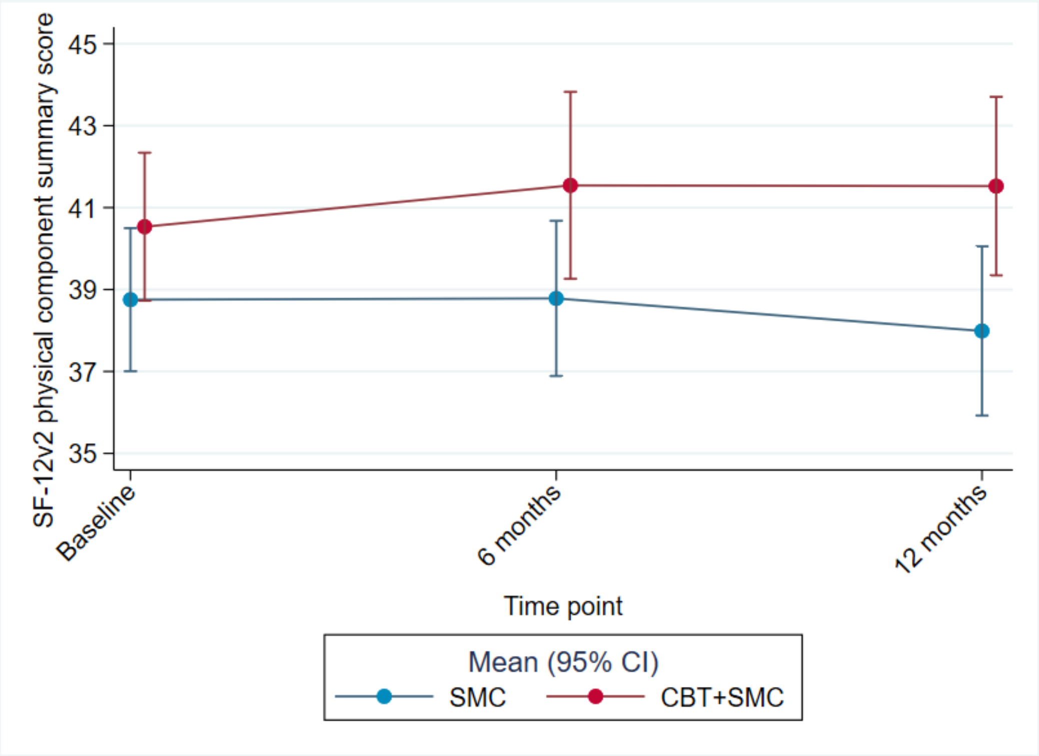

Figure 7 Plot over time of SF-12v2 MCS score (mental health) by trial arm

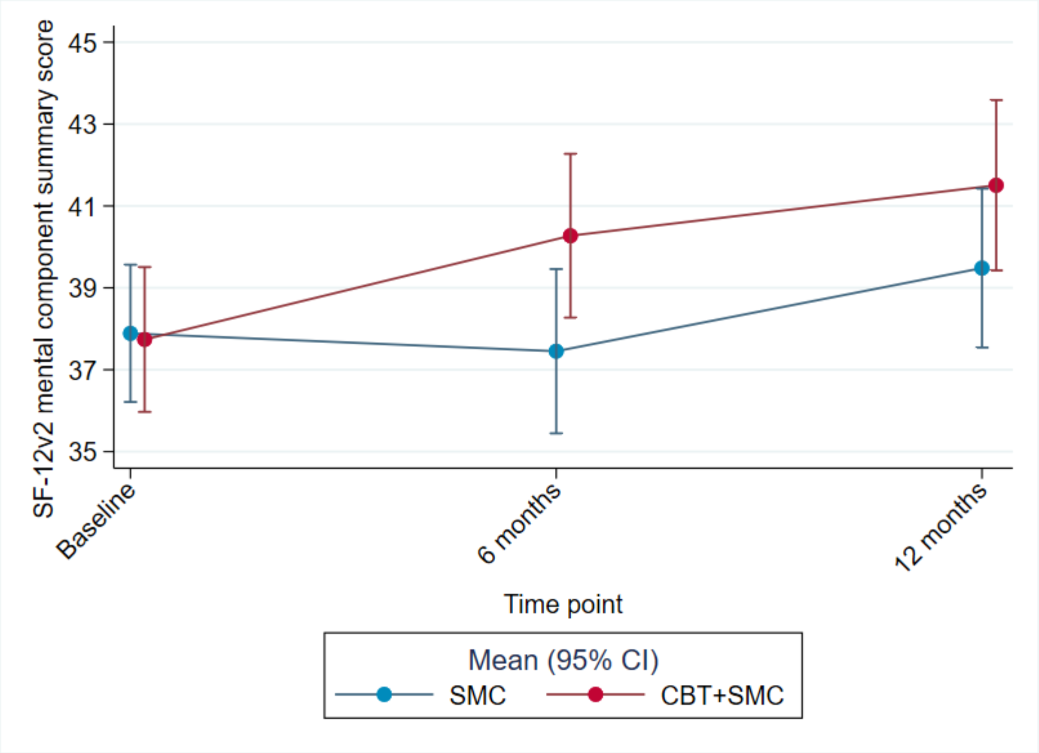

Figure 8 Plot over time of EQ-5D-5L VAS score (health today) by trial arm

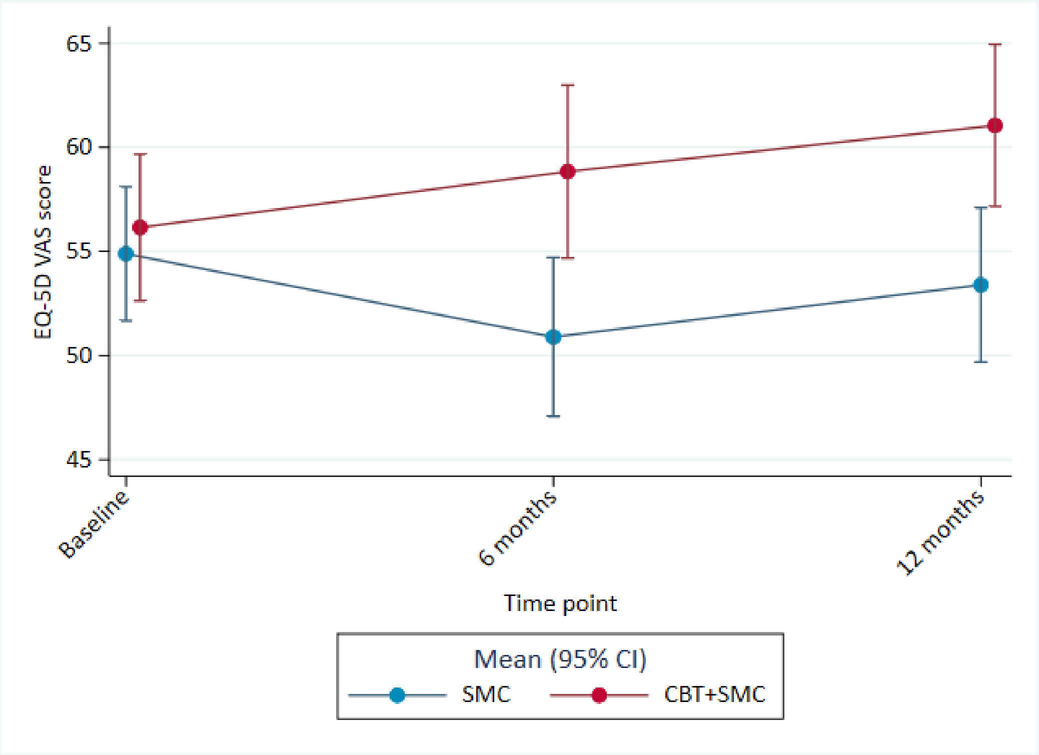

Figure 9 Plot over time of WSAS score (impact on functioning) by trial arm

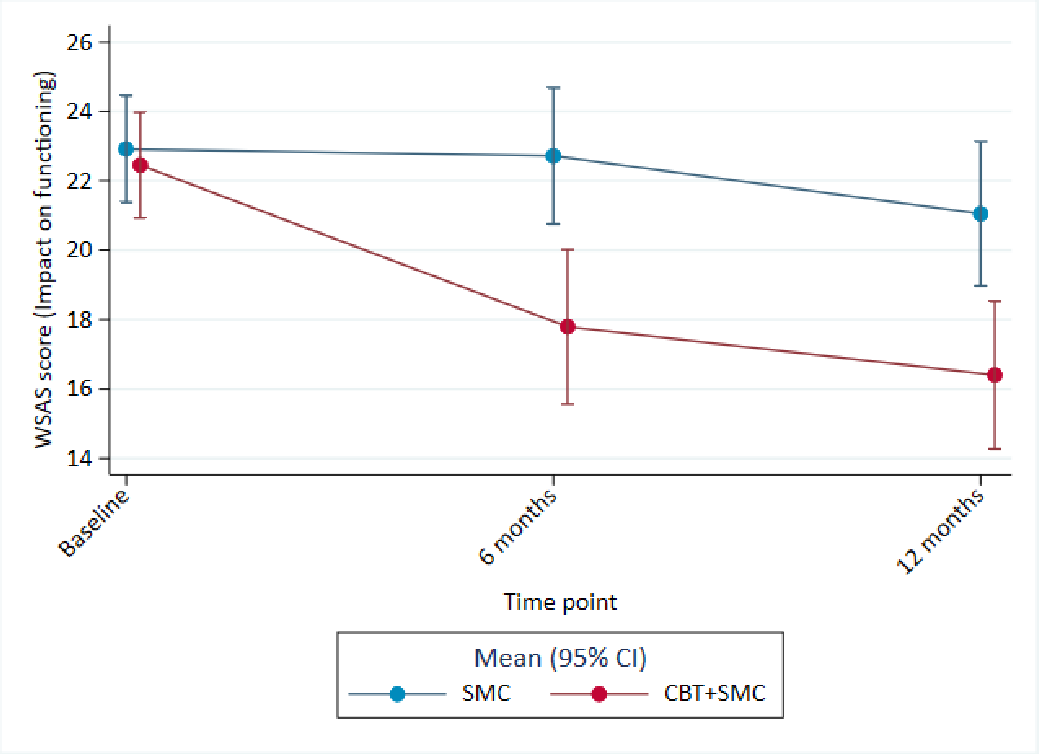

Figure 10 Plot over time of GAD-7 score (anxiety) by trial arm

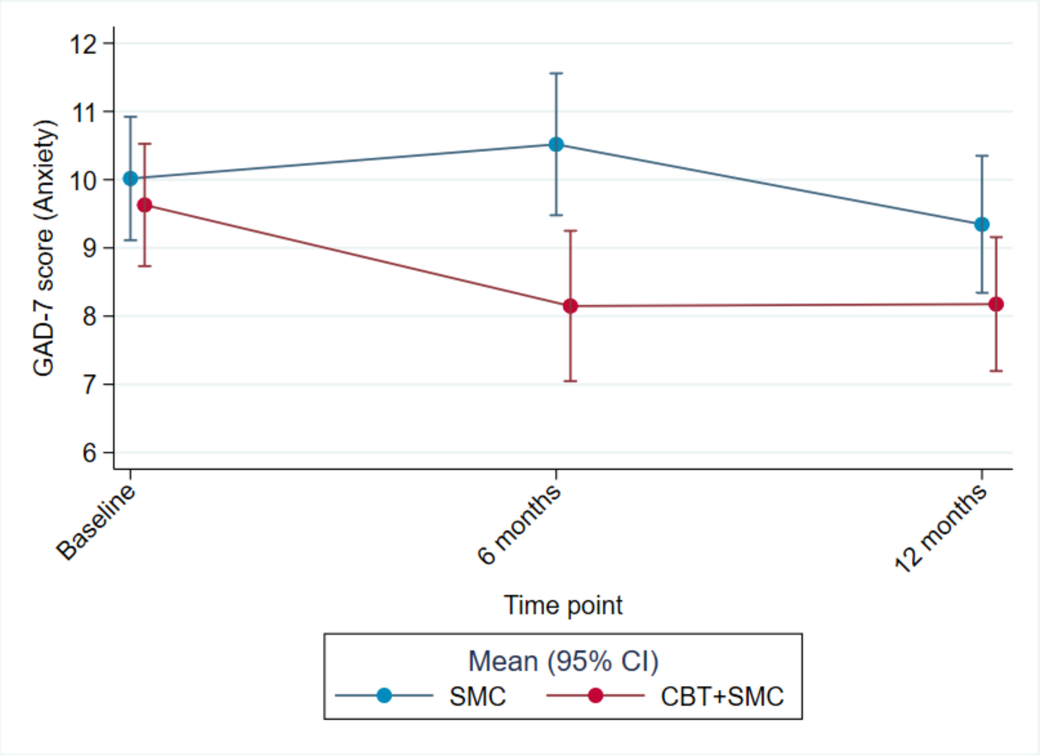

Figure 11 Plot over time of PHQ-9 score (depression) by trial arm

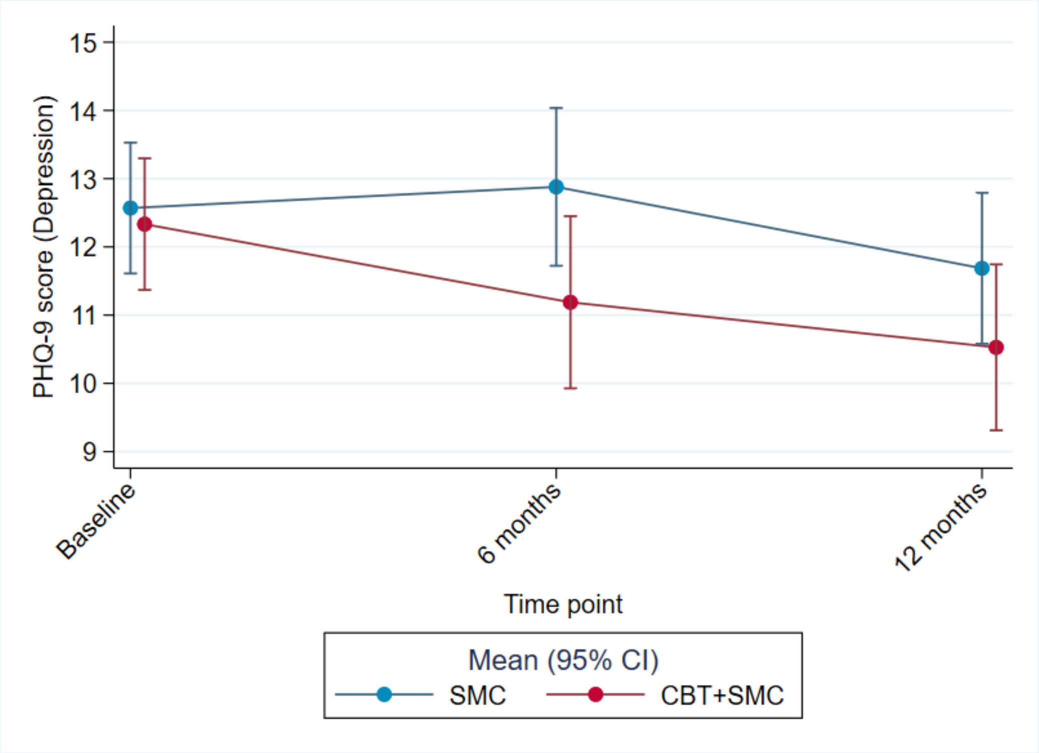

Figure 12 Plot over time of CORE-10 score (psychological distress) by trial arm

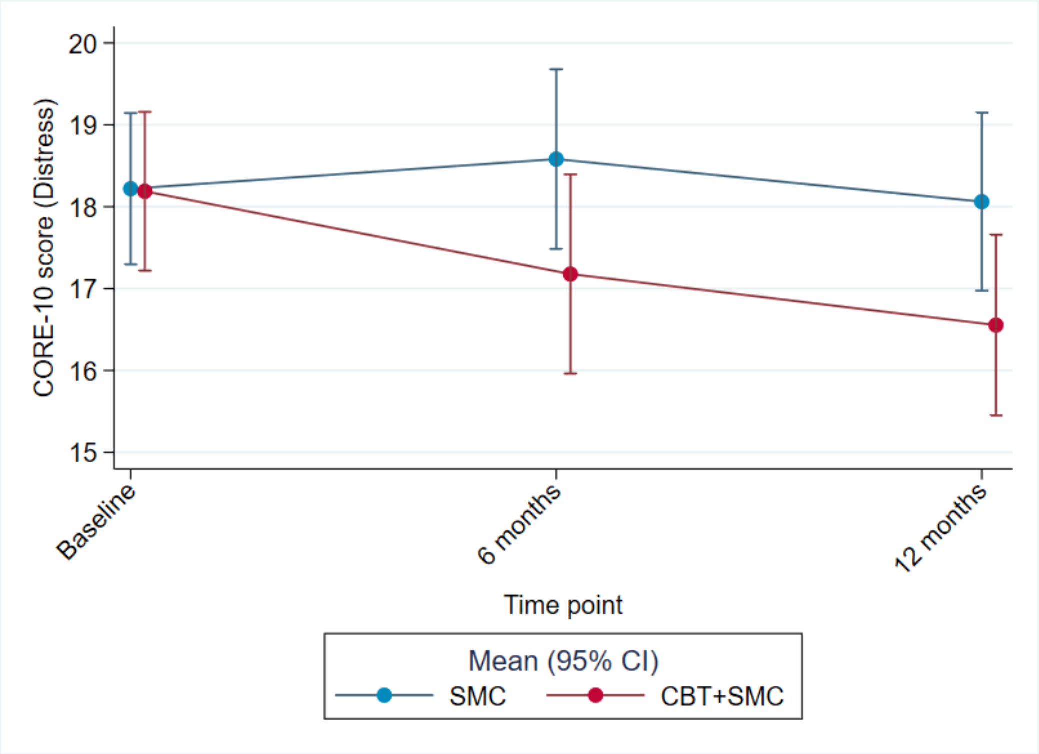

Figure 13 Plot over time of Modified PHQ-15 score (other somatic symptoms) by trial arm

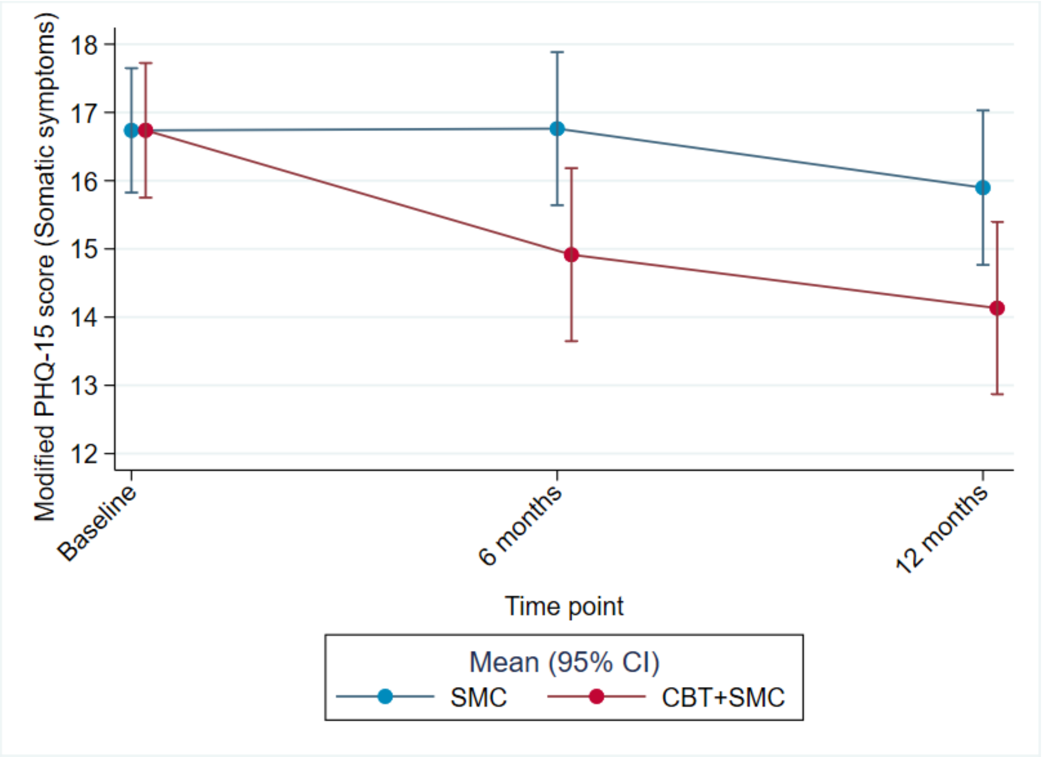

Figure 14 Plot over time of participant-rated clinical global impression (CGI) of change score by trial arm

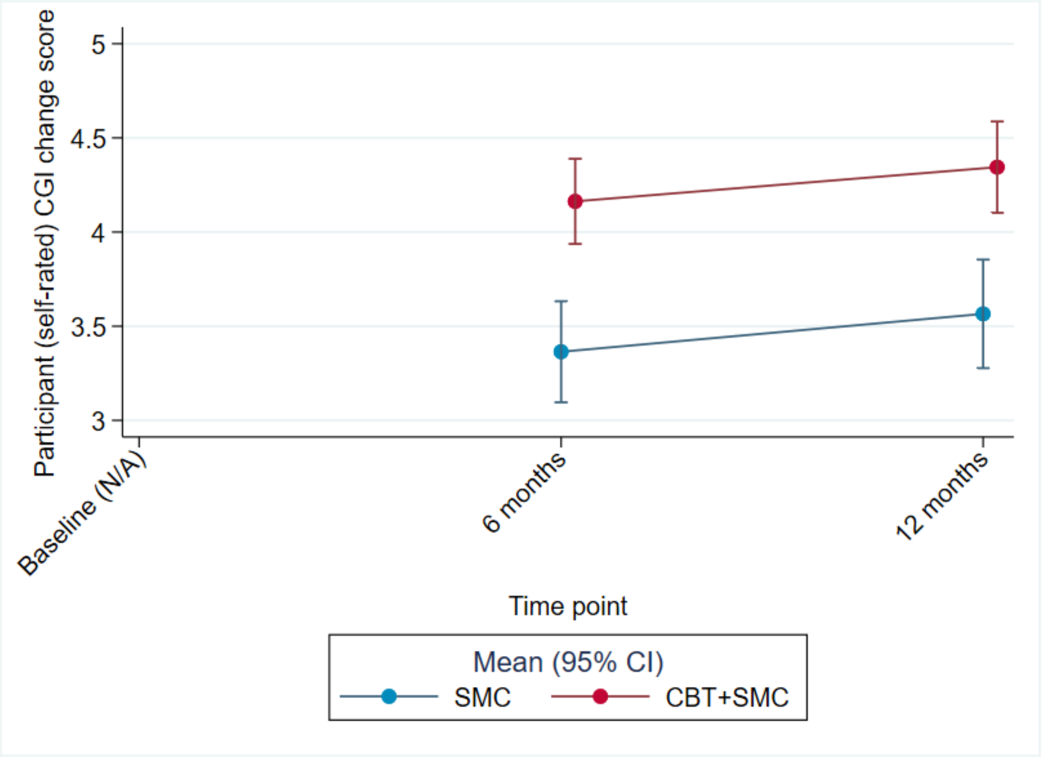

Figure 15 Plot of doctor-rated clinical global impression (CGI) of change score at 12 months by trial arm

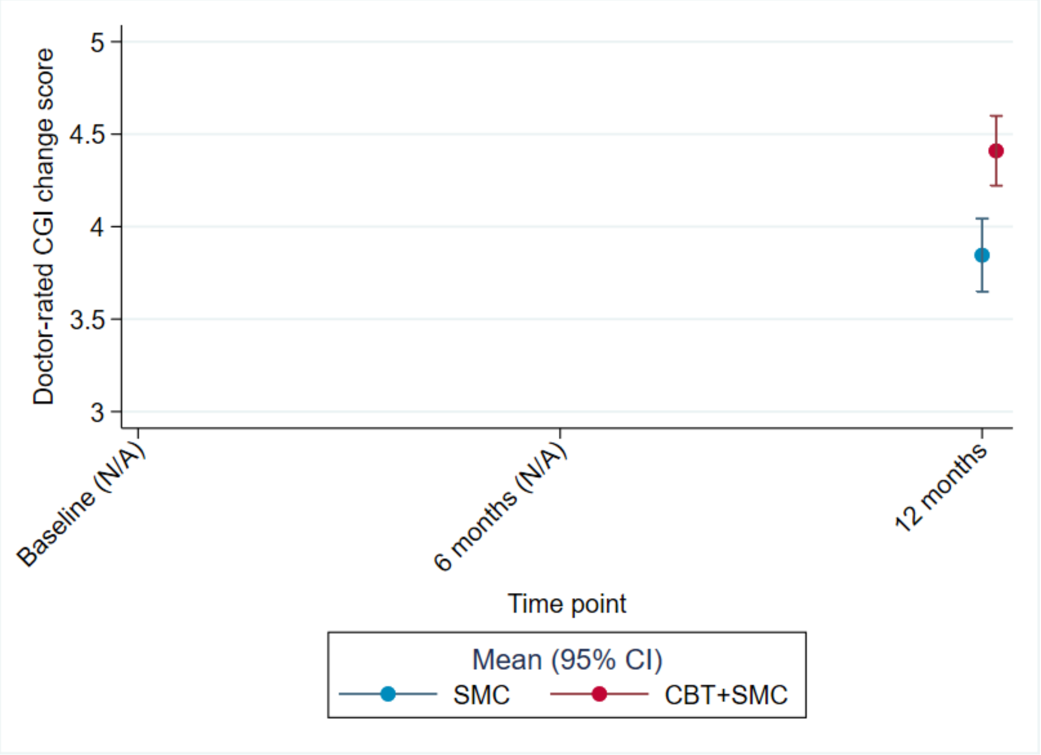

Figure 16 Plot over time of participant-reported satisfaction with treatment by trial arm

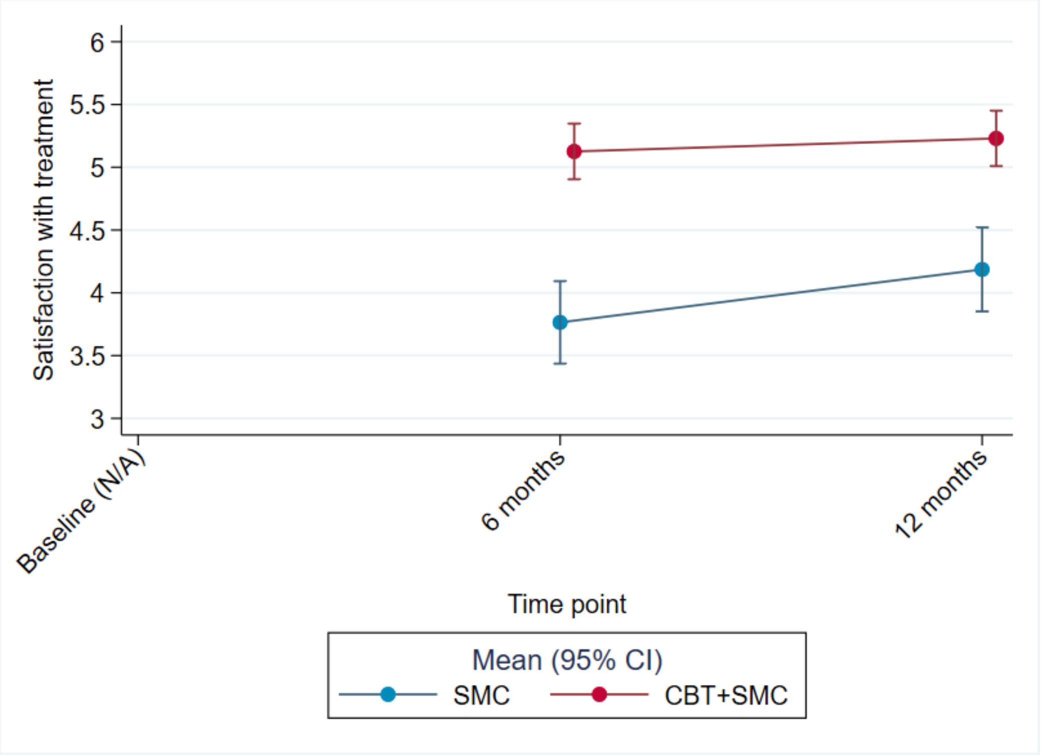

References for citations in the Appendix other than for the protocol document

1 Goldstein LH, Chalder T, Chigwedere C, et al. Cognitive-behavioral therapy for psychogenic nonepileptic seizures: a pilot RCT. *Neurology* 2010; **74**: 1986–94.

2 Goldstein LH, Mellers JDC, Landau S, et al. COgnitive behavioural therapy vs standardised medical care for adults with Dissociative non-Epileptic Seizures (CODES): A multicentre randomised controlled trial protocol. *BMC Neurol* 2015; **15:**98. DOI 10.1186/s12883-015-0350-0

3 Goldstein LH, LaFrance Jnr WC, Mellers JDC, Chalder T. Cognitive Behavioral Based Treatments for Psychogenic Nonepileptic Seizures. In: LaFrance Jnr WC, Schachter SC, eds. Gates and Rowan's Nonepileptic Seizures Cambridge, UK: Cambridge University Press; 2018: 300–9.

4 Lang PJ. Fear reduction and fear behaviour: problems in treating a construct. In: Shilen JM, ed. Research in Psychotherapy (Vol III). Washington DC: American Psychological Association; 1968.

5 Mowrer OH. Learning theory and behaviour. New York: Wiley; 1960.

6 Goldstein LH, Mellers JD. Ictal symptoms of anxiety, avoidance behaviour, and dissociation in patients with dissociative seizures. *J Neurol Neurosurg Psychiatry* 2006; **77**: 616–21.

7 Robinson EJ, Goldstein LH, McCrone P, et al. COgnitive behavioural therapy versus standardised medical care for adults with Dissociative non-Epileptic Seizures (CODES): statistical and economic analysis plan for a randomised controlled trial. *Trials* 2017; **18**: 258.

8 van Buuren S. Multiple imputation of discrete and continuous data by fully conditional specification. *Stat Meth Med Res* 2007; **16**: 219–42.

9 Rawlings GH, Perdue I, Goldstein LH, Carson AJ, Stone J, Reuber M. Neurologists' experiences of participating in the CODES study-A multicentre randomised controlled trial comparing cognitive behavioural therapy vs standardised medical care for dissociative seizures. *Seizure* 2019; **71**: 8–12.

CODES Study Group

Anne-Mary Abe, Naghme Adab, Niruj Agrawal, Holger Allroggen, Debie Alvares, Thomasin Andrews, Heather Angus-Leppan, Julia Aram, Richard Armstrong, Antonio Atalaia, Manny Bagary, Maria Baldellou Lopez, Masha Bennett, Tamsin Black, Daniel Blackburn, Mayur Bodani, Mark Broadhurst, Alice Brockington, Elisa Bruno, Mary Buckley, Christine Burness, Hannah Callaghan, Richard Chalmers, Sam Chong, Muhammad Chowdhury, Fahmida Chowdury, Katia Cikurel, Giovanni Cocco, Hannah Cock, Sarah Cooper, Sarah Cope, Amy Copping, Elana Day, Robert Delamont, Gary Dennis, Christopher Derry, Rebecca Devlin, Jon M. Dickson, Beate Diehl, Clare Donnelly, Susan Duncan, Mark Edwards, Shan Ellawella, Cathy Ellis, Jennifer Elvish, Robert Elwes, Sandra Eriemo, Sofia Eriksson, Kerry Evans, Rafey Faruqui, Sarah Feehan, Gerald Finnerty, Lorena Flores, Nick Firth, Robert Fung, Paula Gardiner, Christopher Graham, Zayn Green-Thompson, Richard Grunewald, Robert Hadden, Khalid Hamandi, Rachel Harding, Sreedharan Harikrishnan, Simon Harrison, Helen Healy, Channa Hewamadduma, Stephen Higgins, Stephen Howell, Heloise Hunt, Abrar Hussain, Motchila Innocente, Graham Jensch, Michael Johnson, Harriet Jordan, Joanna Karlsson, Andrew Kelso, Steven Kemp, Jonathan Knibb, Norman Kock, Michael Koutroumanidis, Stjepana Kovac, Guru Kumar, Andy Laker, Guy Leschziner, Rebecca Liu, Dora Lozsadi, Lea Ludwig, Bridget MacDonald, Lindsey MacGregor, Melissa Maguire, Mark Manford, Davide Martino, Dougall McCorry, Annalisa McGorlick, Kenneth McKeown, Fiona McKevitt, Anna Meadow, Shafqat Memon, Ana Miorelli, Clinton Mitchell, Tejal N. Mitchell, Virginia Moffitt, Nicholas Moran, Aimee Morgan-Boon, John Moriarty, Marco Mula, Nandini Mullatti, Lina Nashef, Daniel O'Hara, Louise Oakley, Suzanne O'Sullivan, Lisa Page, Darshna Patel, Panayiota Petrochilos, Danielle Phoenix, William Pickerell, Thirza Pieters, Norman Poole, Gary Price, David Protheroe, Patrick Pullicino, James Purnell, Jennifer Quirk, Sanjeev Rajakulendran, Julie Read, Basil Ridha, Claire Rockliffe-Fidler, Carrie Rowbottom, Fergus Rugg-Gunn, Amrit Sachar, Romi Saha, Gerard Saldanha, Shanika Samarasekera, Violeta Sanchez Sanchez, Alastair Santhouse, Karen Scholes, Abhijeeth Shetty, Paul Shotbolt, Rachel Simkiss, Jasvinder Singh, Jananee Sivagnanasundaram, Sean Slaght, Philip Smith, Dilraj Sokhi, Biba Stanton, Liya Suvorova, Tayyeb Tahir, Ruth Taylor, Lara Teare, Lorenza Tedesco, James Teo, John Thorpe, Laura Toplis, Myrto Tsakopoulou, Ivona Tylova, Tracey Vick, Jane Vinnicombe , Matthew Walker, Cathy Walsh, Gillian Watson, Thomas Webb, Tim Wehner, Killian Welch, Kirstin Weyrich, Margaret Whittaker, Mirdhu Wickremaratchi, Louise Wicks, Mahinda Yogarajah.
